# ﻿New genera and species of coniferous twig-inhabiting *Rhytismatales* from China

**DOI:** 10.3897/imafungus.16.138790

**Published:** 2025-02-17

**Authors:** Lan Zhuo, Hai-Qi Wang, Peng Zhang, Xiao-Nan Sui, Mei-Jun Guo, Shi-Juan Wang, Cheng-Lin Hou

**Affiliations:** 1 College of Life Science, Capital Normal University, Xisanhuanbeilu 105, Haidian, Beijing 100048, China Capital Normal University Beijing China; 2 Anhui Provincial Key Laboratory for Microbial Control & School of Forestry & Landscape Architecture, Anhui Agricultural University, West Changjiang Road 130, Hefei, Anhui 230036, China Anhui Agricultural University Anhui China

**Keywords:** 23 new taxa, Host organ specificity, Phylogeny, Taxonomy

## Abstract

Species in the order *Rhytismatales* M.E. Barr ex Minter (*Leotiomycetes*, *Ascomycota*) develop on a wide range of host plants, but prefer conifers, such as species of *Cupressaceae* and *Pinaceae*. Conifers, the largest group of gymnosperms, show a high diversity in China. In this study, the species diversity of *Rhytismatales* on twigs of conifers is investigated based on specimens newly collected in China. Morphological characteristics combined with multi-gene phylogenetic analysis (ITS, nrLSU, and mtSSU rDNA) revealed 18 new species, belonging to six new genera (*Abiomyces*, *Cryptococcomyces*, *Labivalidus*, *Neotherrya*, *Pseudococcomyces*, and *Stipamyces*) and three known genera (*Hypoderma*, *Hypohelion*, and *Tryblidiopsis*). Additionally, seven new combinations are proposed. The findings underscore the complexity of fungal taxonomy within *Rhytismatales* and the importance of considering multiple criteria for accurate classification. The study also explores the importance of host specificity for genus and species delimitation within the order. A key to genera and species of *Rhytismatales* on twigs of conifers worldwide is provided.

## ﻿Introduction

The order *Rhytismatales* belongs to Leotiomycetes (Ascomycota). Species within *Rhytismatales* are widely distributed and have been recorded in Asia, Europe, North America, South America, and even the Arctic, with few reports from Africa (Powell 1974; Sherwood 1980; Cannon and Minter 1986; Johnston 1992; Lin et al. 1995a, 1995b; Hou 2004; Hernández et al. 2015; Masumoto 2023). Rhytismatalean fungi are commonly found on the branches or leaves of conifers, *Ericaceae*, herbaceous plants, and some other vascular plants. Conifers are among the most preferred hosts of *Rhytismatales*, with approximately 25% of the existing genera and species—more than 200 species across 23 genera—occurring on coniferous trees. Among these genera, 18 have been established with type specimens from conifers (Darker 1932, 1967; Johnston 1989; Hou 2004; Guo et al. 2024).

Members of *Rhytismatales* on twigs of conifers are usually recorded as endophytes, for example *Tryblidiopsispinastri* (Pers.) P. Karst. (Livsey and Minter 1994), while some are plant pathogens, such as *Coccomyceslijiangensis* C.L. Hou & M. Piepenbr. and *Co.guizhouensis* Y.R. Lin & B.F. Hu cause branch blight in *Pinusarmandii* (Hou and Piepenbring 2007; Lin et al. 1994, 2012).

With approximately 615 extant species, conifers represent roughly two-thirds of the total gymnosperm species and constitute about 39% of the world’s forest cover (Farjon 2010; Christenhusz et al. 2011; Armenise et al. 2012; Wang and Ran 2014). China, especially the Hengduan Mountains in the Yunnan Province, has a high diversity of conifers (Mutke and Barthlott 2005; Li et al. 2009; Liu et al. 2019). Most of the specimens of *Rhytismatales* for the present study were collected in the Hengduan Mountains and its neighboring areas.

For the present study, species diversity, taxonomy and phylogeny of species of *Rhytismatales* on twigs of conifers were investigated based on specimens recently collected in China. The phylogeny is reconstructed based on DNA sequences of multiple loci including the internal transcribed spacer (ITS) region, the large subunit nuclear ribosomal RNA gene (nrLSU), and the small subunit mitochondrial rDNA gene sequences (mtSSU). In the context of the present study, six new genera, 18 new species, and seven new combinations are proposed. Taxonomic positions of known species having molecular sequences are discussed. A key is provided for the identification of genera and species of *Rhytismatales* on conifers worldwide.

## ﻿Materials and methods

### ﻿Specimen collection and isolation

Fresh specimens were collected in China, mostly in Yunnan Province. Specimens were air-dried, placed in paper bags and stored in a cool, dry location in the laboratory for subsequent studies. Ascomata were cut from the twigs of conifers and disinfected in 75% ethanol for 30 s, followed by 10% sodium hypochlorite (NaOCl) for 3 min, washed in sterile water three times, then placed on Petri dishes containing potato dextrose agar (PDA) and incubated at room temperature (20 °C), hyphae emerging from the surface of the ascomata were isolated and subcultured on individual PDA plates. Living cultures of new species from this study were deposited in Capital Normal University Culture Collection Center (**CNUCC**) in China.

### ﻿Morphological studies

Mature ascomata were selected for morphological analyses. External shape, size, color, opening of the ascomata and conidiomata, as well as characteristics of zone lines and other details, were observed and photographed under a Nikon SMZ-1000 stereomicroscope (Japan). Color values were taken from ColorHexa (https://www.colorhexa.com/). For a detailed description of methods for morphological analysis, see Hou et al. (2009) and Wang et al. (2023). Dry specimens were deposited at the Herbarium of the College of Life Science, Capital Normal University (**BJTC**) and Reference Collection of Forest Fungi of Anhui Agricultural University (**AAUF**). New names have been registered in the MycoBank database (http://www.mycobank.org/).

### ﻿Molecular techniques

Genomic DNA was extracted from specimens and cultures with the M5 Plant Genomic DNA Kit (Mei5 Biotechnology Co., Ltd., China) following the manufacturer’s instructions. The ITS regions were amplified with PCR using the primers ITS1f/ITS4 (White et al. 1990; Gardes and Bruns 1993), LR0R/LR5 primers were used for nrLSU (White et al. 1990), and mrSSU1/mrSSU3R primers were used for mtSSU (Zoller et al. 1999). PCR was performed in 25 µL reactions according to Lv (2020) and Sui et al. (2023). The PCR products were sent to Zhongkexilin Biotechnology Co., Ltd. (Beijing, China) for purifying, sequencing and editing.

### ﻿Phylogenetic analysis

The forward and reverse DNA sequences were aligned to generate consensus sequences using SeqMan v.7.1.0 in the DNASTAR Lasergene Core Suite software (DNASTAR Inc., Madison, WI, USA). The newly obtained sequences were submitted to the GenBank database, while additional ITS, nrLSU, and mtSSU rDNA sequences included in this study were downloaded from GenBank and UNITE (Suppl. material 1: table S1). The analysis includes the type and representative species from all known genera with available molecular data within *Cudoniaceae*, *Triblidiaceae*, and *Rhytismataceae* s.s. For polyphyletic genera within *Rhytismataceae* s.l., such as *Lophodermium* Chevall. and *Coccomyces* De Not., a representative species from each clade of these genera was selected. *Peziculacarpinea* (Pers.) Tul. & C. Tul. ex Fuckel (*Helotiales*, *Dermateaceae*) as well as *Cudoniellaclavus* (Alb. & Schwein.) Dennis (*Helotiales*, *Tricladiaceae*) were selected as outgroup based on Lantz et al. (2011) and Guo et al. (2024). The ITS, nrLSU, and mtSSU rDNA datasets were aligned with MAFFT (https://www.ebi.ac.uk/Tools/msa/mafft/), and then manually corrected by eye in Se-Al v.2.03a (Rambaut 2000). Ambiguously aligned regions were not used in the analysis. A combined dataset of ITS, nrLSU, and mtSSU sequences was prepared and analyzed using the maximum parsimony method performed with PAUP* 4.0b10 (Swofford 1998). Maximum parsimony analysis was conducted using heuristic searches with 1,000 replicates of random-addition sequence, tree bisection reconnection (TBR) branch swapping and no maxtree limit. All characteristics were equally weighted and unordered. Gaps were treated as missing data to minimize homology assumptions. A bootstrap analysis was performed with 1,000 replicates, each with 100 random taxon addition sequences. MAXTREES was set to 1,000, and TBR branch swapping was employed. For the Bayesian inference (BI) analysis, MrModeltest 2.3 with the Akaike information criterion (AIC) was used to choose the substitution model for each gene: GTR+I+G for ITS, GTR+I+ G for nrLSU, GTR+I+G for mtSSU. The Bayesian analysis was performed with MrBayes 3.1.2 (Huelsenbeck et al. 2001; Ronquist and Huelsenbeck 2003). The analysis of four chains was conducted for 100 000 000 generations with the default settings and sampled every 100 generations, halting the analysis at an average standard deviation of split frequencies of 0.01. The first 25% of the trees were removed as burn-in. Bayesian posterior probabilities (PP) were obtained from the 50% majority rule consensus of the remaining trees. Maximum likelihood (ML) analysis was performed with IQ-TREE 2.2.0 (Minh et al. 2020), the substitution model for ITS is TIM2e+I+R5, for nrLSU TIM3e+FQ+R5, and for mtSSU K3Pu+F+R4. ML bootstrap replicates (1000) were computed in IQ-TREE using a rapid bootstrap analysis and search for the best-scoring ML tree. We only considered clades supported by bootstrap values (MLB) ≥70% for the ML analysis, supported by bootstrap values (MPB) ≥ 70% for the MP analysis and support by PP ≥ 0.95 for Bayesian inference. The final alignments and the retrieved topologies were deposited in TreeBASE (http://www.treebase.org), under accession ID: 31632.

## ﻿Results

By integrating multi-gene phylogenetic analysis (ITS, nrLSU, mtSSU) with morphological assessments, we investigated the phylogeny and taxonomic positions of *Rhytismatales* species collected from conifer twigs. The results reveal 18 new species, six new genera, and seven new combinations, emphasizing the rich fungal diversity associated with conifers.

### ﻿Molecular phylogeny

**Figure 1.**
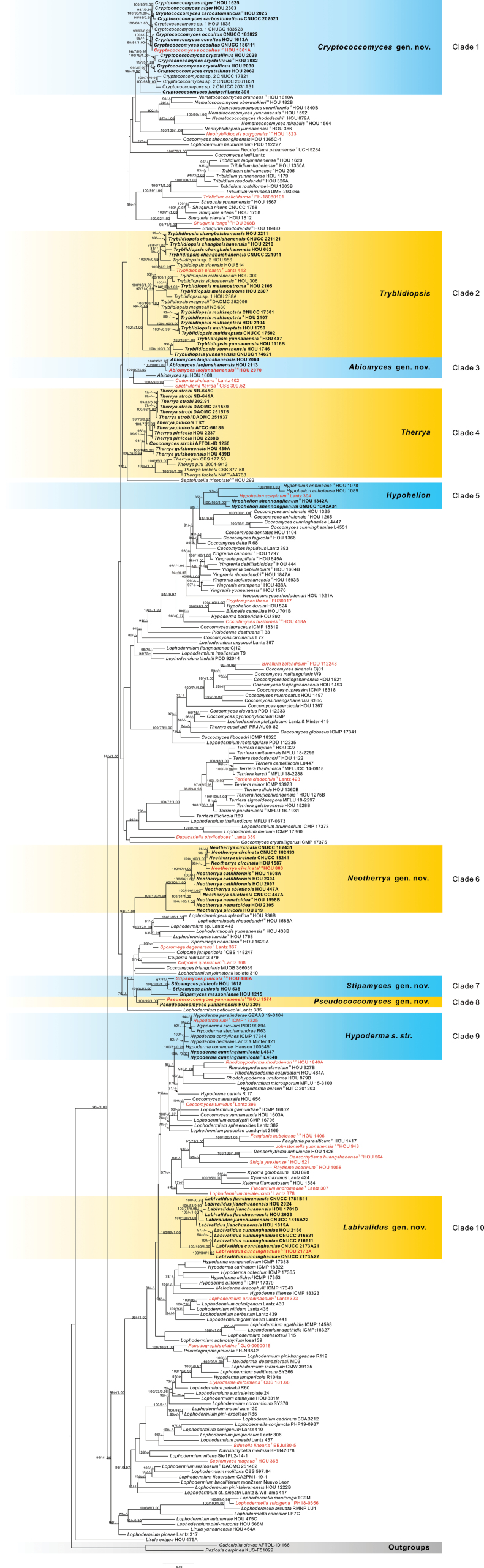
Phylogenetic tree derived from maximum likelihood analysis of combined ITS, nrLSU, and mtSSU rDNA sequences of *Rhytismatales*, using *Cudoniellaclavus* (AFTOL-ID 166) and *Peziculacarpinea* (KUS-F51029) as outgroups. Bootstrap support values for ML analysis (MLB) and MP analysis (MPB) greater than 70% and Bayesian posterior probabilities (PP) greater than 0.95 are given above the nodes. Names of new species and a new combination are written in **bold**. Species name ^T^ – Type species; Species name ^H^ – Holotype.

Seventy new sequences were obtained for ITS rDNA, 61 for nrLSU rDNA, and 61 for mtSSU rDNA regions from newly collected specimens and their cultures. These new sequences were combined with corresponding sequences retrieved from GenBank (Suppl. material 1: table S1). After removing regions impossible to align, the combined matrix included 2070 base positions including 962 of which are parsimony-informative. The maximum parsimony analysis of sequences resulted in one most parsimonious tree (Fig. 1) with a length (TL) of 9560 steps, consistency index (CI) of 0.224, retention index (RI) of 0.692, homoplasy index (HI) of 0.776, and rescaled consistency index (RC) of 0.155. The phylogenetic trees of single loci (Suppl. material 1: figs S1–S3) and the nrLSU-mtSSU phylogenetic tree (Suppl. material 1: fig. S4) show topologies similar to those of the ITS-nrLSU-mtSSU phylogenetic tree.

### ﻿Taxonomy

The new sequence data of *Rhytismatales* species obtained in the context of this study are distributed across ten clades that can be considered as genera (Fig. 1). Four of these genera are already established, namely *Hypoderma*, *Hypohelion*, *Therrya*, and *Tryblidiopsis*, while the remaining six monophyletic groups are proposed as genera that are new to science.

A total of 18 new species have been found, with 12 of them belonging to the six newly described genera. These new species are *Abiomyceslaojunshanensis*, *Cryptococcomycescarbostomaticus*, *Cry.crystallinus*, *Cry.niger*, *Cry.occultus*, *Labivaliduscunninghamiae*, *Neotherryacatilliformis*, *N.circinate*, *N.nematoidea*, *N.pinicola*, *Pseudococcomycesyunnanensis*, and *Stipamycesmassonianae*. Additionally, one new species, *Hypodermacunninghamiicola*, is assigned to the genus *Hypoderma*, one species, *Hypohelionshennongjianum*, to the genus *Hypohelion*, and four species, *Tryblidiopsischangbaishanensis*, *Try.melanostroma*, *Try.multiseptata*, and *Try.yunnanensis*, are introduced in *Tryblidiopsis*. Furthermore, several taxonomic reassignments are made based on the molecular phylogenetic evidence. *Colpomajuniperi* (P. Karst.) Dennis is reclassified from *Colpoma* to *Cryptococcomyces*. *Lophodermiumjianchuanense* C.L. Hou & M. Piepenbr. is transferred from *Lophodermium* to *Labivalidus*. *Therryaabieticola* C.L. Hou & M. Piepenbr. is moved from *Therrya* to *Neotherrya*. Additionally, several species previously classified in *Coccomyces* are transferred into diverse genera: *Coccomycespinicola* R.H. Lei & C.L. Hou to *Stipamyces*, *Co.guizhouensis* and *Co.strobi* J. Reid & Cain to *Therrya*, and *Parvacoccumpini* R.S. Hunt & A. Funk to *Therrya*. Based on molecular phylogenetic evidence, there are additional samples that represent unnamed species, which are not further described here as the ascomata of these specimens are not yet mature.

#### ﻿ Abiomyces

Taxon classificationAnimaliaRhytismatalesRhytismataceae

Lan Zhuo & C.L. Hou gen. nov.

088C8184-676C-5F25-8B76-021B5A7D08CE

856584

##### Etymology.

Referring to the type that growing on twigs of *Abies*.

##### Diagnosis.

This new genus is similar to *Coccomyces* De Not. on coniferous twigs and barks, but differs from *Coccomyces* by truncate acsi. *Abiomyces* is also similar to *Therrya* Sacc., but differs in having simple paraphyses, which do not form an epithecium, and aseptate ascospores.

##### Type species.

*Abiomyceslaojunshanensis* Lan Zhuo & C.L. Hou, described below.

##### Sexual morph.

***Ascomata*** on twigs of *Abies*, scattered, round or slightly irregular, black (#000000), opening by radial or irregular splits to expose a yellow (#ffd400) to pale orange (#ffa500) hymenium. In median vertical section, ascomata intracortical. ***Lips*** absent. ***Covering stroma*** well developed. ***Basal Covering stroma*** poorly developed. ***Internal matrix of Covering stroma*** present, consisting of hyaline, thin-walled, angular cells with some irregular crystalloids and short hyphae. ***Subhymenium*** consisting of small, hyaline cells. ***Paraphyses*** filiform, simple. ***Asci*** clavate, thin-walled, J–, 8-spored. ***Ascospores*** aseptate, filiform, hyaline, without a gelatinous sheath.

##### Asexual morph.

***Conidiomata*** and ***zone lines*** not seen.

##### Notes.

Phylogenetically, the molecular sequences of species belonging to this new genus form a distinct lineage (Clade 3, Fig. 1) with high support (MLB = 100%, MPB = 97%, PP = 1.00). In the phylogenetic tree, the sequences of species of *Abiomyces* are clustered with sequences of species of *Cudoniaceae*, a group of fungi that live in the soil and leaf litter. Some species of *Cudoniaceae* are associated with conifer, so that the species of *Abiomyces* and *Cudoniaceae* may have evolved together in the same habitat.

Morphologically, the ascomatal shape of *A.laojunshanensis* is similar to those of *Coccomyces* and *Therrya* that develop on twigs of conifers. Until now, 16 species of *Coccomyces* have been reported growing on coniferous twigs and bark (https://www.ars.usda.gov/). However, except for *Co.mertensianae*, other species in *Coccomyces* lacks apically truncate asci. Species of *Therrya* differ from *A.laojunshanensis* by apically inflated paraphyses embedded in gelatinous sheaths forming an epithecium and multi-septate ascospores (Reid and Cain 1961; Sherwood 1980). Based on the separate phylogenetic positions and morphological characteristics, we propose *Abiomyces* as a separate genus.

#### ﻿ Abiomyces laojunshanensis

Taxon classificationAnimaliaRhytismatalesRhytismataceae

Lan Zhuo & C.L. Hou sp. nov.

9B97E290-87CC-58D8-9DBE-511AE0D5CA21

856588

Figs 2 , 3

##### Etymology.

Referring to the host genus *Abies*.

##### Diagnosis.

This new species is similar to *Coccomycesmertensianae* Sherwood, but *Abiomyceslaojunshanensis* has longer, fili-fusiform ascospores.

##### Type.

CHINA, Yunnan Province, Lijiang, Laojunshan, 26.6310°N, 99.7227°E, alt. ca. 3930 m, on twigs of *Abiesgeorgei* Orr (*Pinaceae*), 17 Aug 2023, C.L. Hou, L. Zhuo, and S.Y. Zhao, HOU 2070 (BJTC 2023200, holotype).

##### Sexual morph.

***Ascomata*** on twigs, scattered, not associated with pale areas. In surface view, ascomata subround or slightly irregular, 500–1250 × 750–1500 µm, black (#000000), erumpent from the bark, opening by radial splits to expose a yellow (#ffd400) to pale orange (#ffa500) hymenium. ***Lips*** absent. In median vertical section, ascomata intracortical. ***Covering stroma*** 30–50 μm thick near center of ascomata, not extending to the basal Covering stroma, consisting of an outer layer of remains of the host cortex and an inner layer of carbonized, angular to globose cells. ***Basal Covering stroma*** poorly developed, consisting of carbonized, angular to globose cells. ***Internal matrix of Covering stroma*** 375–470 µm thick, consisting of hyaline, thin-walled, angular cells with some irregular crystalloids and short hyphae. ***Subhymenium*** 40–60 µm thick, consisting of small, hyaline cells. ***Paraphyses*** aseptate, filiform, not branched, not swollen at tips, 130–150 × ca. 1 µm, covered by a thin gelatinous sheath. ***Asci*** ripening sequentially, clavate, apex truncate, 80–90 × 8–10 µm, stalked, thin-walled, J–, 8-spored. ***Ascospores*** aseptate, filiform to fusiform, 50–65 × 3 μm, hyaline, without a gelatinous sheath.

##### Asexual morph.

***Conidiomata*** and ***zone lines*** not seen.

##### Additional specimens examined.

CHINA, Yunnan Province, Lijiang, Laojunshan, 26.6314°N, 99.7235°E, alt. ca. 3880 m, on twigs of *Abiesgeorgei*, 17 Aug. 2023, C.L. Hou, L. Zhuo, and S.Y. Zhao, HOU 2064 (BJTC 2023194); CHINA, Yunnan Province, Dali Bai Autonomous Prefecture, Mount Cangshan, 25.6644°N, 100.1024°E, alt. ca. 3830 m, on twigs of *Abiesdelavayi* Franch. (*Pinaceae*), 18 Aug. 2023, C.L. Hou, L. Zhuo, and S.Y. Zhao, HOU 2113 (BJTC 2023244).

##### Distribution.

Known only from Yunnan Province, China.

##### Notes.

Morphologically, *A.laojunshanensis* resembles *Coccomycesmertensianae* growing on twigs of *Tsugamertensiana* (Bong.) Carrière, however, *Coccomycesmertensianae* has septate paraphyses and shorter (19–25 × 2.5–3.0 µm), narrowly clavate ascospores. The multi-locus gene analysis indicates that the molecular sequences of *A.laojunshanensis* form an independent clade with high support (MLB = 100%, MPB = 100%, PP = 1.00). These sequences do not cluster with any species of *Coccomyces*, especially the type *Co.tumidus* (Fr.) De Not. Therefore, *A.laojunshanensis* is considered as a species new to science.

**Figure 2.**
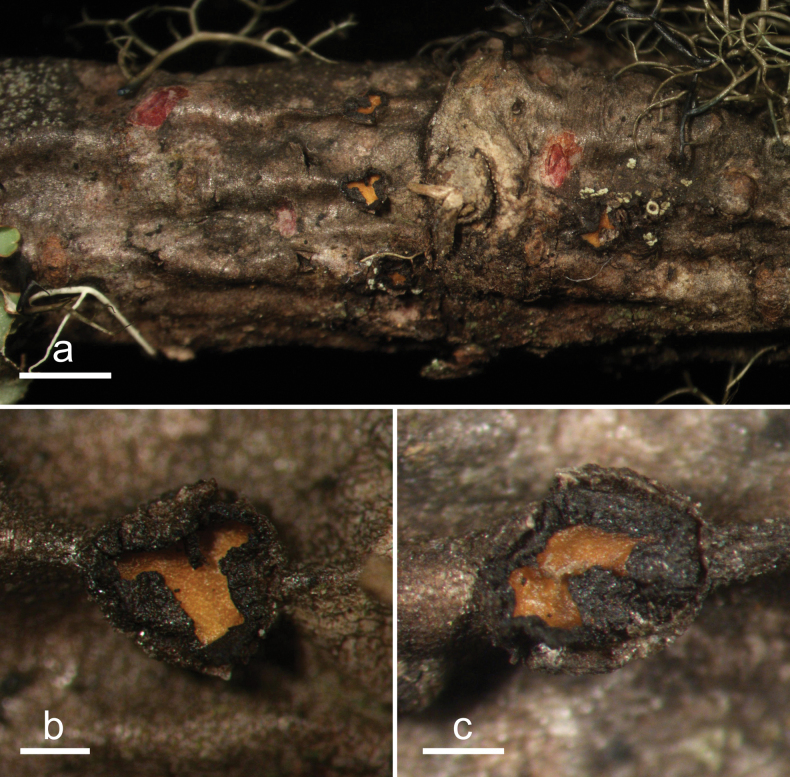
*Abiomyceslaojunshanensis* (HOU 2070/BJTC 2023200, holotype) **a** ascomata on a twig of *Abiesgeorgei***b, c** mature ascomata. Scale bars: 3 mm (**a**); 500 μm (**b, c**).

**Figure 3.**
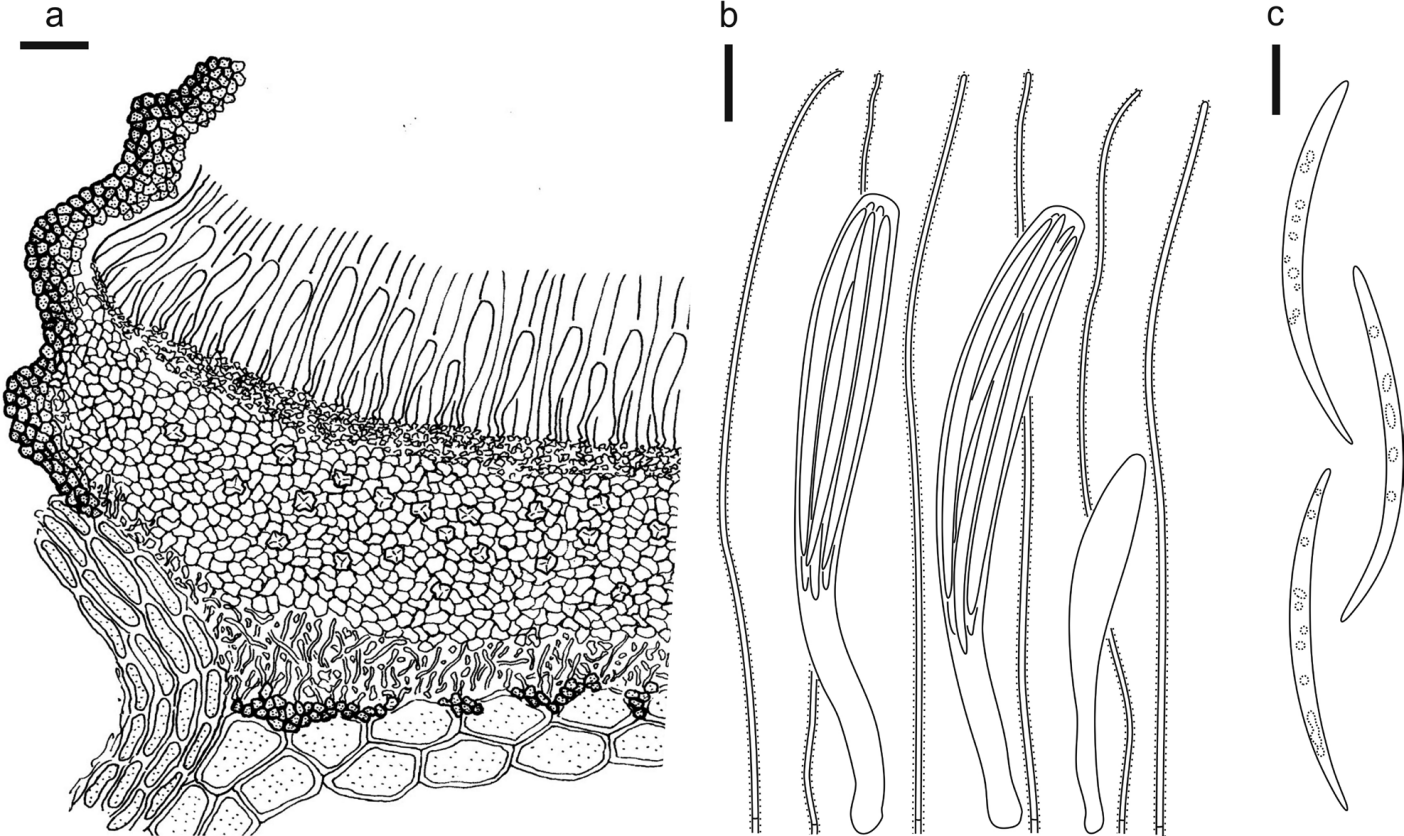
*Abiomyceslaojunshanensis* on *Abiesgeorgei* (HOU 2070/BJTC 2023200, holotype) **a** part of an ascoma in vertical section, the internal matrix of Covering stroma filled with numerous crystals **b** paraphyses, mature asci with ascospores, and immature ascus. **c** Liberated ascospores. Scale bars: 50 μm (**a**); 10 μm (**b, c**).

#### ﻿ Abiomyces

Taxon classificationAnimaliaRhytismatalesRhytismataceae

sp.

44D94737-B375-5A71-B6FB-A881D11B9BA6

##### Specimen examined.

CHINA, Yunnan Province, Lijiang, Laojunshan, 26.6323°N, 99.7252°E, alt. ca. 3880 m, on twigs of *Abiesgeorgei*, 16 Jul. 2020, C.L. Hou, M.J. Guo, and Q.T. Wang, HOU 1608ZI (BJTC 2020049).

##### Notes.

In the multigene phylogenetic analysis, the molecular sequence of this specimen is closely related to *A.laojunshanensis* with strong support (MLB = 100%, MPB = 97%, PP = 1.00). The ITS rDNA sequence similarity of these two species is 94%. Unfortunately, ascomata could not be found for this species. Mature ascomata are necessary to clarify specific morphological features of the species.

#### ﻿ Cryptococcomyces

Taxon classificationAnimaliaRhytismatalesRhytismataceae

Lan Zhuo & C.L. Hou gen. nov.

19919607-8256-575F-ABBD-2EC55EA80B6F

856607

##### Etymology.

*Crypto* (Latin) = hidden, referring to the hidden ascomata of the type and morphological similarity with species of the genus *Coccomyces*.

##### Diagnosis.

Species of this new genus are morphologically similar to some species of *Coccomyces* that inhabit coniferous twigs and bark, but are phylogenetically distant from the type of *Coccomyces*, *Co.tumidus*, which inhabits leaves, as shown in the phylogenetic tree.

##### Type.

*Cryptococcomycesoccultus* Lan Zhuo & C.L. Hou, described below.

##### Sexual morph.

***Ascomata*** on twigs of *Juniperus* spp., scattered, round, elliptical or slightly irregular, black (#000000) or concolorous with the surface of the substrate, opening by radial or irregular splits to expose a pale yellow (#ffff9d) to pale orange (#ffa500) hymenium. ***Lips*** absent. Ascomata intracortical. ***Covering stroma*** well developed. ***Basal Covering stroma*** absent or present, consisting of carbonized, thick-walled, angular to globose cells. ***Internal matrix of Covering stroma*** absent or present, consisting of hyaline, thin-walled, angular cells and short hyphae; in some species, the matrix is filled with crystals. ***Subhymenium*** flat or slightly depressed, consisting of hyaline textura porrecta. ***Paraphyses*** filiform, branched or not branched, not swollen at tips. ***Asci*** ripening sequentially, clavate, thin-walled, J–, 8-spored. ***Ascospores*** aseptate, filiform, hyaline, covered or not covered by a gelatinous sheath.

##### Asexual morph.

***Conidiomata*** and ***zone lines*** not seen.

##### Notes.

Phylogenetically, molecular sequence data obtained from specimens cited below for species of *Cryptococcomyces* cluster together with a sequence in GenBank labelled “*Colpomajuniperi* (P. Karst.) Dennis Lantz 395” with high support (MLB = 99%, PP = 0.97; Clade 1, Fig. 1). According to molecular sequence data, this clade is distantly related to the type species of *Colpoma*, *Colpomaquercinum* (Pers.) Wallr. Species of *Cryptococcomyces* do not belong to the genus *Colpoma* according to molecular sequence data.

In addition, the round ascomata with radial or irregular openings of the species in the new genus resemble those of the genus *Coccomyces* rather than *Colpoma* (Sherwood 1980; Johnston 1991). However, the clade formed by molecular sequences of *Cryptococcomyces* is phylogenetically distinct from the type of *Coccomyces*, *Co.tumidus*. Therefore, these species are not congeneric with *Coccomyces*. Additionally, species in *Cryptococcomyces* all grow on twigs of *Juniperus* spp. Based on morphological characteristics, phylogenetic analysis, and the host relationship, *Cry.juniperi* is proposed to belong to the genus *Cryptococcomyces*.

#### ﻿ Cryptococcomyces carbostomaticus

Taxon classificationAnimaliaRhytismatalesRhytismataceae

Lan Zhuo & C.L. Hou sp. nov.

BA9292B3-8F9C-5819-9AFC-4A3F6696DC31

856610

Figs 4 , 5

##### Etymology.

Carbo- (Latin) = carbonized, -stoma (Latin) = opening, referring to strongly carbonized opening of ascomata.

##### Diagnosis.

This new species is similar to *Cryptococcomycesniger*, but differs by the presence of a basal Covering stroma and ascospores covered by a thick gelatinous sheath.

##### Type.

CHINA, Yunnan Province, Lijiang, Laojunshan, 26.6418°N, 99.7673°E, alt. ca. 3495 m, on twigs of *Juniperussquamata* D. Don (*Cupressaceae*), 16 Aug. 2023, C.L. Hou, L. Zhuo, and S.Y. Zhao, HOU 2025 (BJTC 2023155, holotype).

##### Sexual morph.

***Ascomata*** on twigs, erumpent from bark, scattered, not associated with pale areas. In surface view, ascomata round or slightly irregular, 1000–2000 × 750–1000 µm, black (#000000), opening by irregular splits to expose a yellow (#ffe562) hymenium. ***Lips*** absent. In median vertical section, ascomata intracortical. ***Covering stroma*** 100–175 μm thick near the center of ascomata, consisting of an outer layer of remains of the host cortex, an inner layer of carbonized, angular to globose cells, and an innermost layer of hyaline textura prismatica. ***Basal Covering stroma*** 25–35 µm thick, consisting of 3–7 μm diam., thick-walled, angular to globose cells. ***Internal matrix of Covering stroma*** 40–65 µm thick, consisting of hyaline, thin-walled, angular cells, filled with irregular crystals. ***Subhymenium*** 25–20 µm thick, consisting of hyaline textura porrecta. ***Paraphyses*** aseptate, filiform, not branched, curled or coiled at their tips, 130–145 × 1–1.5 µm, covered by a thin gelatinous sheath. ***Asci*** ripening sequentially, clavate, apex acute, 80–120 × 6–8 µm, thin-walled, J–, 8-spored. ***Ascospores*** aseptate, filiform, tapering towards the apex, 30–40 × 1–1.5 μm, hyaline, covered by a ca. 2 µm thick gelatinous sheath.

##### Asexual morph.

***Conidiomata*** and ***zone lines*** not seen.

##### Distribution.

Known only from Yunnan Province, China.

##### Notes.

The multi-locus gene analysis indicates that the sequences of *Cry.carbostomaticus* form a well-supported clade sister to the sequences of *Cry.niger*. *Cryptococcomycescarbostomaticus* is morphologically similar to *Cry.niger*, but the latter has a covering stroma without textura prismatica, no basal Covering stroma, and ascospores lack a thick gelatinous sheath. Therefore, *Cry.carbostomaticus* is considered to be a distinct species.

**Figure 4.**
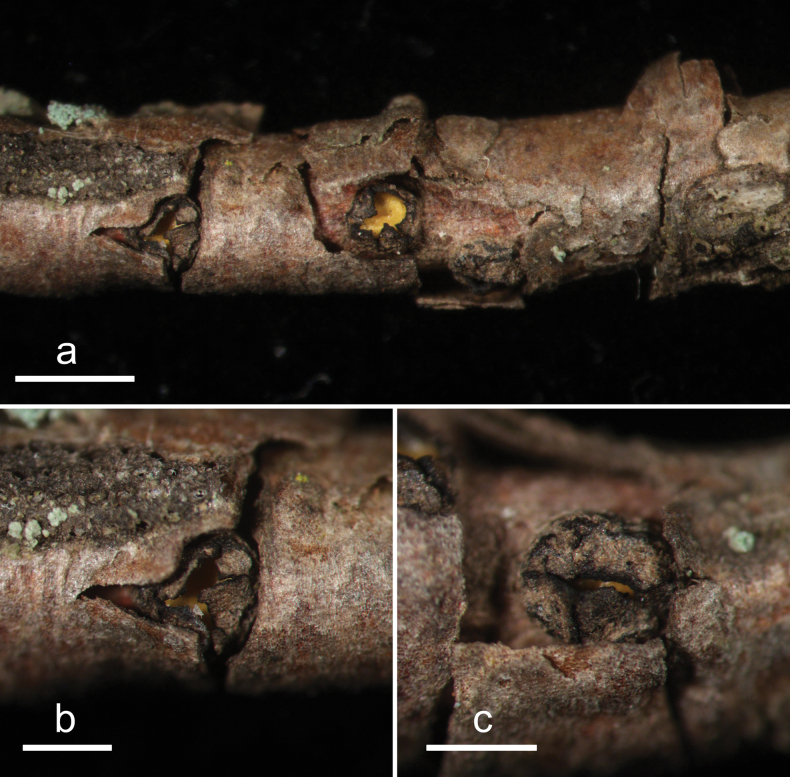
*Cryptococcomycescarbostomaticus* (HOU 2025/BJTC 2023155, holotype) **a** ascomata on a twig of *Juniperussquamata***b, c** mature ascomata. Scale bars: 2 mm (**a**); 1 mm (**b, c**).

**Figure 5.**
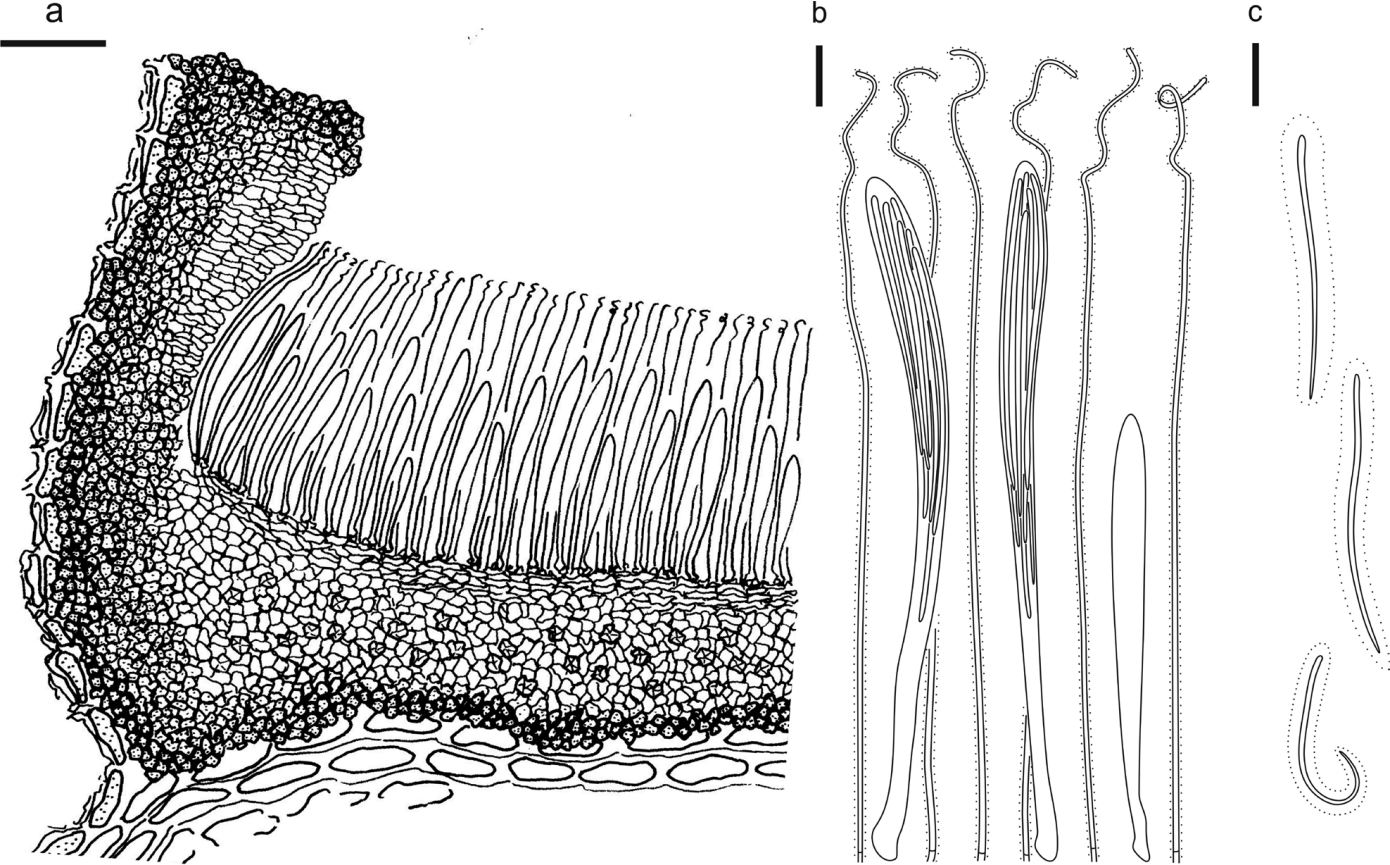
*Cryptococcomycescarbostomaticus* on *Juniperussquamata* (HOU 2025/BJTC 2023155, holotype) **a** part of an ascoma in vertical section, covering stroma with hyaline textura prismatica **b** paraphyses, mature asci with ascospores, and immature ascus. **c** Liberated ascospores. Scale bars: 100 μm (**a**); 10 μm (**b, c**).

#### ﻿ Cryptococcomyces crystallinus

Taxon classificationAnimaliaRhytismatalesRhytismataceae

Lan Zhuo & C.L. Hou sp. nov.

42ED08D7-6FBD-54D6-AF25-B66133FFE10F

856611

Figs 6 , 7

##### Etymology.

*Crystallinus* (Latin) = crystal, referring to the internal matrix of Covering stroma filled with numerous crystals.

##### Diagnosis.

This new species is similar to *Cryptococcomycesniger*, but differs by an internal matrix of the Covering stroma almost fully filled with crystals.

##### Type.

CHINA, Yunnan Province, Lijiang, Laojunshan, 26.6326°N, 99.7199°E, alt. ca. 3950 m, on twigs of *Juniperussquamata* (*Cupressaceae*), 17 Aug. 2023, C.L. Hou, L. Zhuo, and S.Y. Zhao, HOU 2082 (BJTC 2023212, holotype).

##### Sexual morph.

***Ascomata*** on twigs, erumpent from bark, scattered, not associated with pale areas. In surface view, ascomata round or slightly irregular, 1000–2000 × 750–1000 µm, black (#000000), opening by radial splits to expose a yellow (#ffd400) to pale orange (#ffa500) hymenium. ***Lips*** absent. In median vertical section, ***covering stroma*** 80–100 μm thick near the center of ascomata, consisting of an outer layer of carbonized, angular to globose cells and an inner layer of hyaline, thin-walled, angular to globose cells. ***Basal Covering stroma*** absent. ***Internal matrix of Covering stroma*** 240–260 µm thick, consisting of hyaline, thin-walled, angular cells, with abundant irregular crystals in the matrix. ***Subhymenium*** 15–20 µm thick, consisting of hyaline textura porrecta. ***Paraphyses*** aseptate, filiform, not branched, slightly curved at tips, 110–130 × 1–2 µm, covered by a thin gelatinous sheath. ***Asci*** ripening sequentially, clavate, apex acute, 85–105 × 7–8 µm, stalked, thin-walled, J–, 8-spored. ***Ascospores*** aseptate, filiform, tapering towards apex, 40–60 × 1–2 μm, hyaline, with a gelatinous cap.

##### Asexual morph.

***Conidiomata*** and ***zone lines*** not seen.

##### Additional specimens examined.

CHINA, Yunnan Province, Lijiang, Laojunshan, 26.6319°N, 99.7252°E, alt. ca. 3860 m, on twigs of *Juniperussquamata* (*Cupressaceae*), 16 Aug. 2023, C.L. Hou, L. Zhuo, and S.Y. Zhao, HOU 2028 (BJTC 2023158); 26.6318°N, 99.7250°E, alt. ca. 3930 m, on twigs of *J.squamata*, 16 Aug. 2023, C.L. Hou, L. Zhuo, and S.Y. Zhao, HOU 2030 (BJTC 2023160); 26.6319°N, 99.7244°E, alt. ca. 3890 m, on twigs of *J.squamata*, 17 Aug. 2023, C.L. Hou, L. Zhuo, and S.Y. Zhao, HOU 2062 (BJTC 2023192).

##### Distribution.

Known only from Yunnan Province, China.

##### Notes.

The multi-locus gene analysis shows that the sequences of *Cry.crystallinus* form a well-supported clade sister to the sequences of *Cryptococcomyces* sp. 2. However, the specimens of *Cryptococcomyces* sp. 2 do not have any mature ascoma; therefore, these two species cannot be compared morphologically. The ITS rDNA sequence similarity between these two species is 92%, so we treat *Cry.crystallinus* as a separate species. Morphologically, *Cry.crystallinus* is similar to *Cry.niger*, but *Cry.niger* differs by an internal matrix of the Covering stroma lacking crystals.

**Figure 6.**
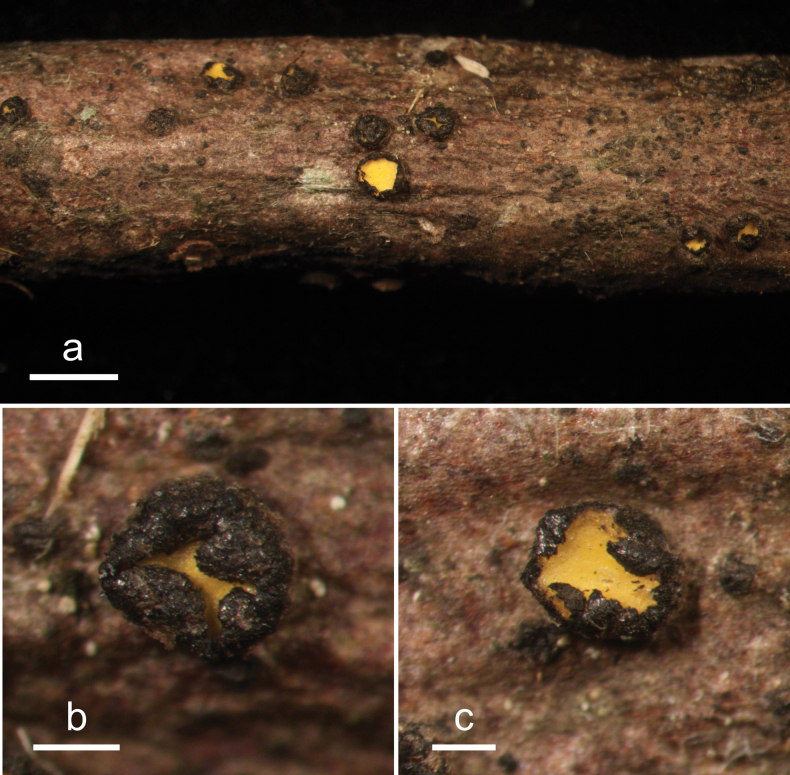
*Cryptococcomycescrystallinus* (HOU 2082/BJTC 2023212, holotype) **a** ascomata on a twig of *Juniperussquamata***b, c** mature ascomata. Scale bars: 2 mm (**a**); 500 μm (**b, c**).

**Figure 7.**
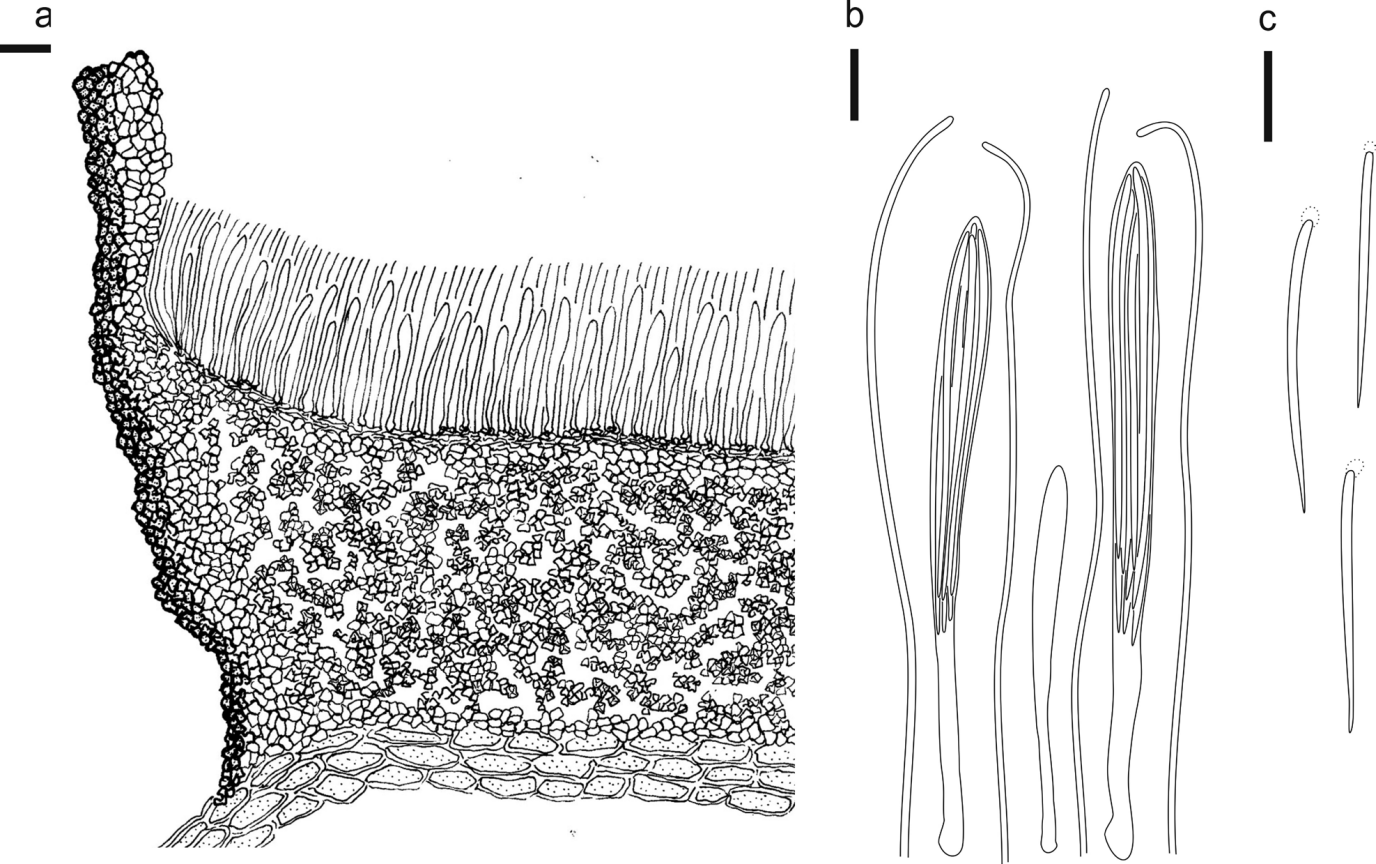
*Cryptococcomycescrystallinus* on *Juniperussquamata* (HOU 2082/BJTC 2023212, holotype) **a** part of an ascoma in vertical section, the internal matrix of Covering stroma filled with numerous crystals **b** paraphyses, mature asci with ascospores, and immature ascus. **c** Liberated ascospores with gelatinous cap. Scale bars: 100 μm (**a**); 10 μm (**b, c**).

#### ﻿ Cryptococcomyces juniperi

Taxon classificationAnimaliaRhytismatalesRhytismataceae

(P. Karst.) Lan Zhuo & C.L. Hou comb. nov.

B5577C4F-CA49-5DCD-817C-CE40B0687271

856609

Coccomyces juniperi P. Karst., Bidr. Känn. Finl. Nat. Folk 19: 254. 1871. Basionym.
= Colpomajuniperi (P. Karst.) Dennis, Kew Bull. [12](3): 401. 1958.

##### Type.

Finland, on bark of *Juniperuscommunis* L. (*Cupressaceae*), Fennia et Lapponia, collected throughout the year (exact date unknown), P. Karsten (A holotype needs to be designated).

##### Notes.

Phylogenetically, a non-type sequence of *Cry.juniperi* clusters with other species of *Cryptococcomyces*, which also occur on *Juniperus* spp. Dennis (1958) transferred *Coccomycesjuniperi* to the genus *Colpoma* based on its slender paraphyses with spirally coiled tips (Dennis 1958). However, species of the genus *Colpoma* usually have elongated and curved ascomata with a single longitudinal opening slit, whereas *Cryptococcomycesjuniperi* has subglobose ascomata with 3 to 6 triangular flaps (Dennis 1958; Johnston 1991; Lin et al. 2012). Therefore, based on their phylogenetic position, morphological similarities, and host relationship, *Cry.juniperi* is proposed to belong to the genus *Cryptococcomyces*.

#### ﻿ Cryptococcomyces niger

Taxon classificationAnimaliaRhytismatalesRhytismataceae

Lan Zhuo & C.L. Hou sp. nov.

6F09E651-FA17-5E88-A0F5-47E9A3FD68AA

856612

Figs 8 , 9

##### Etymology.

*Niger* (Latin) = black, referring to the black ascomata.

##### Diagnosis.

This new species is similar to *Cryptococcomycescrystallinus*, but differs by having an internal matrix of the Covering stroma without crystals.

##### Type.

CHINA, Yunnan Province, Lijiang, Laojunshan, 26.6322°N, 99.7250°E, alt. ca. 3880 m, on twigs of *Juniperussquamata* (*Cupressaceae*), 23 Jun. 2020, C.L. Hou, M.J. Guo, and Q.T. Wang, HOU 1625 (BJTC 2020067, holotype).

##### Sexual morph.

***Ascomata*** on twigs, scattered, not associated with pale areas. In surface view, ascomata elliptical to round, 800–1000 × 900–1200 µm, black (#000000), erumpent from bark, opening by radial splits to expose a pale yellow (#ffff9d) to yellow (#ffd400) hymenium. ***Lips*** absent. In median vertical section, ascomata intracortical. ***Covering stroma*** 110–125 μm thick near the center of the ascomata, not extending to the basal Covering stroma, consisting of an outer layer of remains of the host cortex, an inner layer of carbonized, angular to globose cells, and an innermost layer of hyaline angular cells embedded in the hyaline gelatinous matrix. ***Basal Covering stroma*** absent. ***Internal matrix of Covering stroma*** 90–110 µm thick, consisting of hyaline, thin-walled short hyphae in a gelatinous matrix. ***Subhymenium*** 20–30 µm thick, consisting of hyaline textura porrecta. ***Paraphyses*** aseptate, filiform, not branched, curled or coiled at tips, 125–150 × 1–2 µm, covered by a thin gelatinous sheath. ***Asci*** ripening sequentially, clavate, apex obtuse to acute, 72–95 × 6–8 µm, stalked, thin-walled, J–, 8-spored. ***Ascospores*** aseptate, filiform, tapering towards the apex, 30–40 × 1 μm, hyaline.

##### Asexual morph.

***Conidiomata*** and ***zone lines*** not seen.

##### Additional specimens examined.

CHINA, Yunnan Province, Lijiang, Laojunshan, 26.6322°N, 99.7250°E, alt. ca. 3880 m, on twigs of *Juniperussquamata* (*Cupressaceae*), 26 Jul. 2024, C.L. Hou, L. Zhuo, and X.N. Sui, HOU 2303 (BJTC 2024153).

##### Distribution.

Known only from Yunnan Province, China.

##### Notes.

The multi-locus gene analysis indicates that the molecular sequences of *Cry.niger* form a well-supported clade sister to *Cryptococcomycescarbostomaticus*. *Cry.carbostomaticus* is distinguished by presence of a basal Covering stroma and textura prismatica on innermost layer of the covering stroma. Morphologically, *Cry.niger* is similar to *Coccomycespetersii*, and both of these two species occur on *Juniperus* spp. However, the covering stroma of *Co.petersii* is composed entirely of carbonized globose cells, whereas that of *Cry.niger* includes an innermost layer of hyaline angular cells embedded in a hyaline gelatinous matrix. Additionally, *Co.petersii* has larger asci (85–110 × 8–10 µm). The basal Covering stroma of *Co.petersii* is reduced to a subiculum of dark brown hyphae, while *Cry.niger* lacks a basal Covering stroma but possesses a well-developed internal matrix within the Covering stroma, consisting of hyaline hyphae.

**Figure 8.**
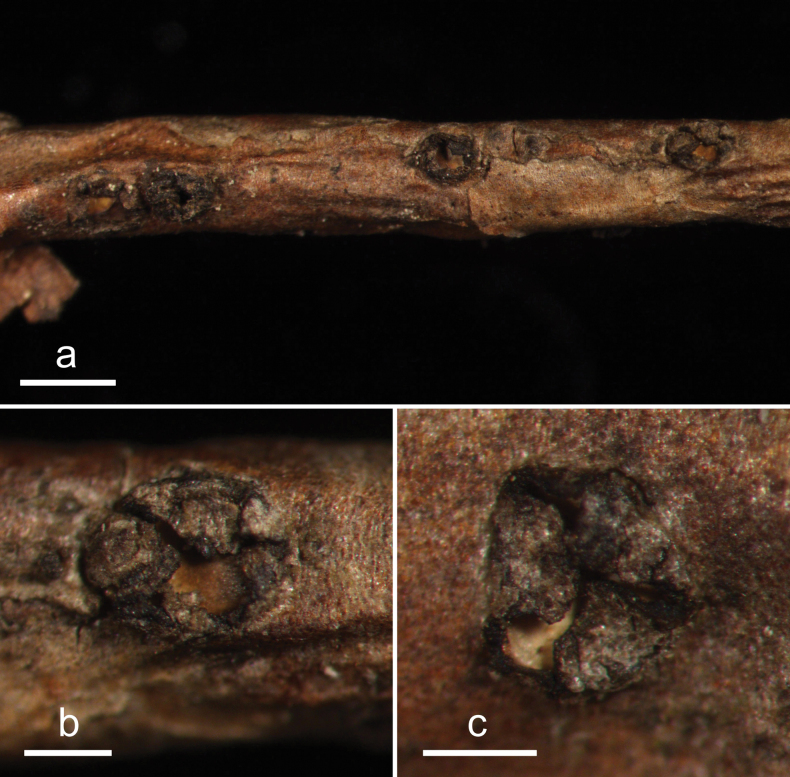
*Cryptococcomycesniger* (HOU 1625/BJTC 2020067, holotype) **a** ascomata on a twig of *Juniperussquamata***b, c** mature ascomata. Scale bars: 2 mm (**a**); 500 μm (**b, c**).

**Figure 9.**
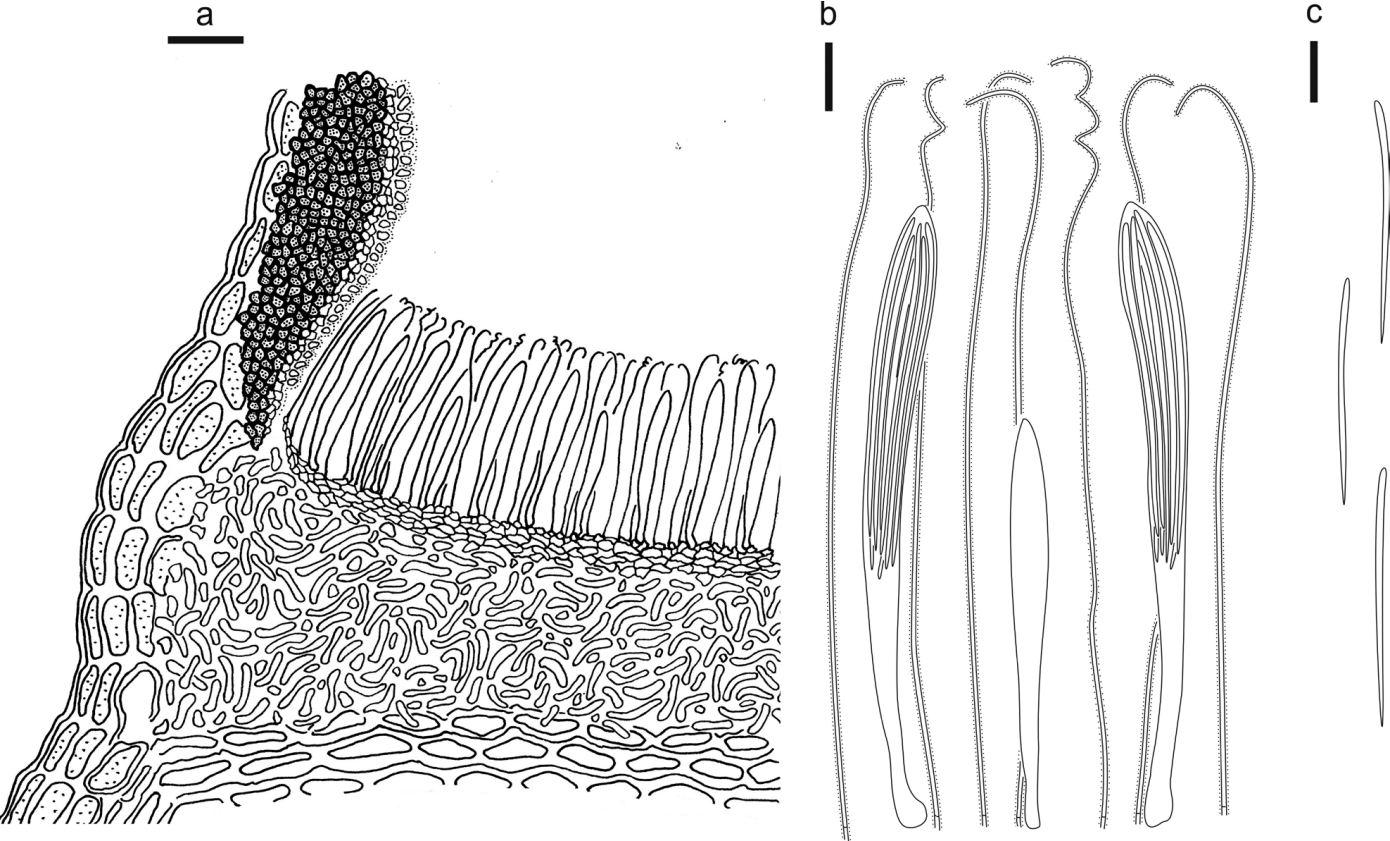
*Cryptococcomycesniger* on *Juniperussquamata* (HOU 1625/BJTC 2020067, holotype) **a** part of an ascoma in vertical section, innermost layer of covering stroma with hyaline angular cells embedded in the hyaline gelatinous matrix **b** paraphyses, mature asci with ascospores, and immature ascus **c** liberated ascospores. Scale bars: 100 μm (**a**); 10 μm (**b, c**).

#### ﻿ Cryptococcomyces occultus

Taxon classificationAnimaliaRhytismatalesRhytismataceae

Lan Zhuo & C.L. Hou sp. nov.

41537303-A752-5A65-8537-40D62B330561

856613

Figs 10 , 11

##### Etymology.

*Occultus* (Latin) = hidden, referring to the ascomata surface being concolorous with the surface of the substrate.

##### Diagnosis.

This new species is similar to *Cryptococcomycescarbostomaticus*, but differs by the absence of hyaline textura prismatica as innermost layer of the covering stroma.

##### Type.

CHINA, Yunnan Province, Kunming, Jiaozi Mountains, 26.0861°N, 102.8492°E, alt. ca. 3810 m, on twigs of *Juniperussquamata* (*Cupressaceae*), 23 Jun. 2021, C.L. Hou, M.J. Guo, and H. Zhou, HOU 1861A (BJTC 2021172, holotype).

##### Sexual morph.

***Ascomata*** on twigs, scattered, not associated with pale areas. In surface view, ascomata elliptical to round, 1600–2100 × 1400–2100 μm, concolorous with surface of substrate, erumpent through host epidermis, opening by irregular splits to expose pale yellow (#ffff9d) hymenium. ***Lips*** absent. In median vertical section, ascomata intracortical. ***Covering stroma*** 125–145 μm thick near the center of the ascomata, extending to the basal Covering stroma, consisting of an outer layer of remains of host cortex and an inner layer of carbonized, angular to globose cells. ***Excipulum*** present, formed by marginal paraphyses. ***Basal Covering stroma*** poorly developed, consisting of carbonized, angular to globose cells. ***Internal matrix of Covering stroma*** 95–130 µm thick, consisting of hyaline, thin-walled, angular cells and short hyphae embedded in gelatinous mucus. ***Subhymenium*** 20–30 µm thick, consisting of hyaline textura porrecta. ***Paraphyses*** aseptate, filiform, not branched, slightly curved at tips, 130–140 × 1–2 µm, covered by a thin gelatinous sheath. ***Asci*** ripening sequentially, clavate, apex obtuse to acute, 80–120 × 6–8 μm, stalked, thin-walled, J–, 8-spored. ***Ascospores*** aseptate, filiform, 30–40 × 1 μm, hyaline.

##### Asexual morph.

***Conidiomata*** and ***zone lines*** not observed.

##### Additional specimens examined.

CHINA, Yunnan Province, Lijiang, Laojunshan, 26.6433°N, 99.7675°E, alt. ca. 3500 m, on twigs of *Juniperussquamata* (*Cupressaceae*), 16 Jul. 2020, C.L. Hou, M.J. Guo and Q.T. Wang, HOU 1613 (BJTC 2020055); Kunming, Jiaozi Mountains, 26.0841°N, 102.8405°E, alt. ca. 3570 m, on twigs of *J.squamata*, 23 Jun. 2021, C.L. Hou, M.J. Guo and H. Zhou, HOU 1838 (BJTC 2021149).

##### Distribution.

Known only from Yunnan Province, China.

##### Notes.

Phylogenetically, molecular sequences of *Cry.occultus* forms a distinct clade with high support values (MLB = 98%, MPB = 97%, PP = 1.00) together with the sequences of *Cry.carbostomaticus*, *Cry.niger*, and *Cryptococcomyces* sp. 1. However, the values of similarity of the ITS rDNA sequences of *Cry.occultus* with *Cry.carbostomaticus*, *Cry.niger*, and *Cryptococcomyces* sp. 1 are 97%, 96%, and 98%, respectively. Morphologically, *Cry.niger* differs from *Cry.occultus* by black, smaller (800–1000 × 900–1200 µm) ascomata, which protrude from the surface of the host; *Cry.carbostomaticus* differs from *Cry.occultus* by the presence of hyaline textura prismatica in the innermost layer of the covering stroma. Unfortunately, ascomata in the specimen of *Cryptococcomyces* sp. 1 are not mature enough to observe microscopic features. *Cryptococcomycesoccultus* is morphologically similar to *Coccomyceslijiangensis* on *Pinusarmandii* (*Pinaceae*), but *Co.lijiangensis* has a well-developed basal Covering stroma (900–1200 µm), and the internal matrix of the Covering stroma consists of hyaline hyphae and numerous crystals variable in shape and size. Therefore, based on phylogenetic and morphological differences, *Cry.occultus* is designated as a new species. It is the type of the genus *Cryptococcomyces* because it possesses the most typical traits of ascomata covered by bark.

**Figure 10.**
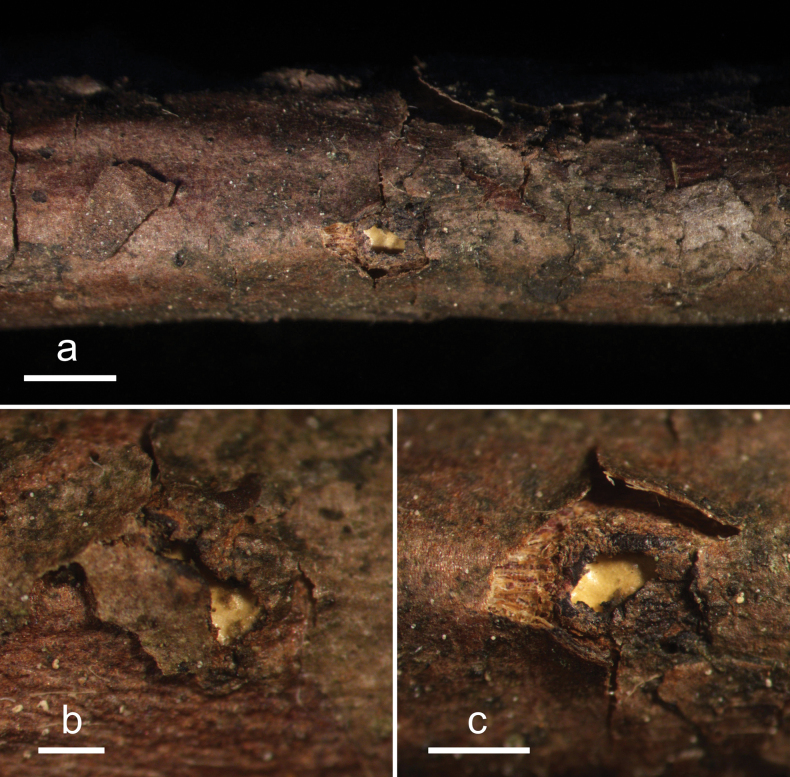
*Cryptococcomycesoccultus* (HOU 1861A/BJTC 2021172, holotype) **a** ascomata on a twig of *Juniperussquamata***b, c** mature ascomata. Scale bars: 2 mm (**a**); 500 μm (**b**); 1 mm (**c**).

**Figure 11.**
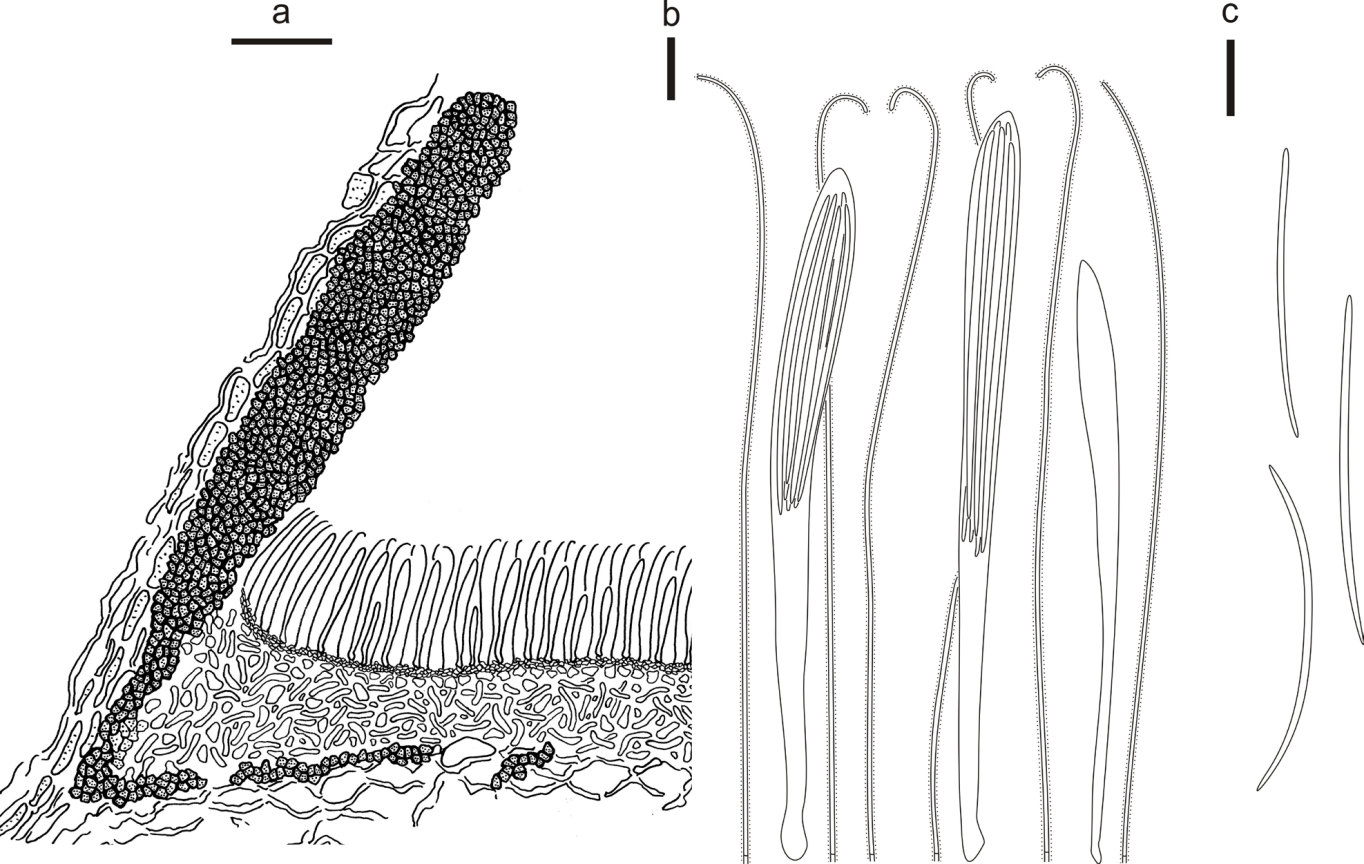
*Cryptococcomycesoccultus* on *Juniperussquamata* (HOU 1861A/BJTC 2021172, holotype) **a** part of an ascoma in vertical section **b** paraphyses, mature asci with ascospores, and immature ascus **c** liberated ascospores. Scale bars: 100 μm (**a**); 10 μm (**b, c**).

#### ﻿ Cryptococcomyces

Taxon classificationAnimaliaRhytismatalesRhytismataceae

sp. 1

1C0A9D2E-DB73-5137-8038-8F371AF8196B

Fig. 12

##### Specimen examined.

CHINA, Yunnan Province, Lijiang, Laojunshan, 26.6413°N, 99.7672°E, alt. ca. 3500 m, on twigs of *Juniperus* sp. (*Cupressaceae*), 21 Jun. 2021, C.L. Hou, M.J. Guo, and H. Zhou, HOU 1835 (BJTC 2021146).

##### Notes.

In the multi-gene phylogenetic tree, the sequences of this undescribed species form a highly supported sister clade with the sequences of *Cry.carbostomaticus* and *Cry.niger*. The similarity of the ITS rDNA sequences of *Cryptococcomyces* sp. 1 with *Cry.carbostomaticus* and *Cry.niger* are 98% and 96%, respectively. As mature ascomata were not found in this specimen, it was not possible to conduct detailed morphological studies.

#### ﻿ Cryptococcomyces

Taxon classificationAnimaliaRhytismatalesRhytismataceae

sp. 2

E3F48638-1CDF-5DEB-8EB0-BFC98AE5512F

##### Cultures examined.

CHINA, Yunnan Province, Lijiang, Laojunshan, 26.6315°N, 99.7249°E, alt. ca. 3900 m, isolated from *Juniperus* sp. (*Cupressaceae*), 16 Aug. 2023, C.L. Hou, L. Zhuo, and S.Y. Zhao, CNUCC 2031A31; 26.6316°N, 99.7253°E, alt. ca. 3855 m, isolated from *Juniperus* sp., 21 Jun. 2021, C.L. Hou, M.J. Guo and H. Zhou, CNUCC 17821; 26.6319°N, 99.7243°E, alt. ca. 3890 m, isolated from *Juniperus* sp., 17 Aug. 2023, C.L. Hou, L. Zhuo, and S.Y. Zhao, CNUCC 2061B31.

##### Notes.

In the multi-gene phylogenetic tree, the molecular sequences of this unnamed species cluster together with the molecular sequence of *Cryptococcomycescrystallinus*. The ITS rDNA similarity between these two species is 92%. It is worth noting that while processing other ascomata from these specimens, we obtained isolates of this species. However, the observed ascomata did not match the morphological characteristics of this genus. Since multiple isolates of this species were obtained from different specimens, sequencing errors or other potential mistakes have been ruled out. We hypothesize the presence of an undiscovered cryptic species, although no corresponding ascomata have yet been found.

**Figure 12.**
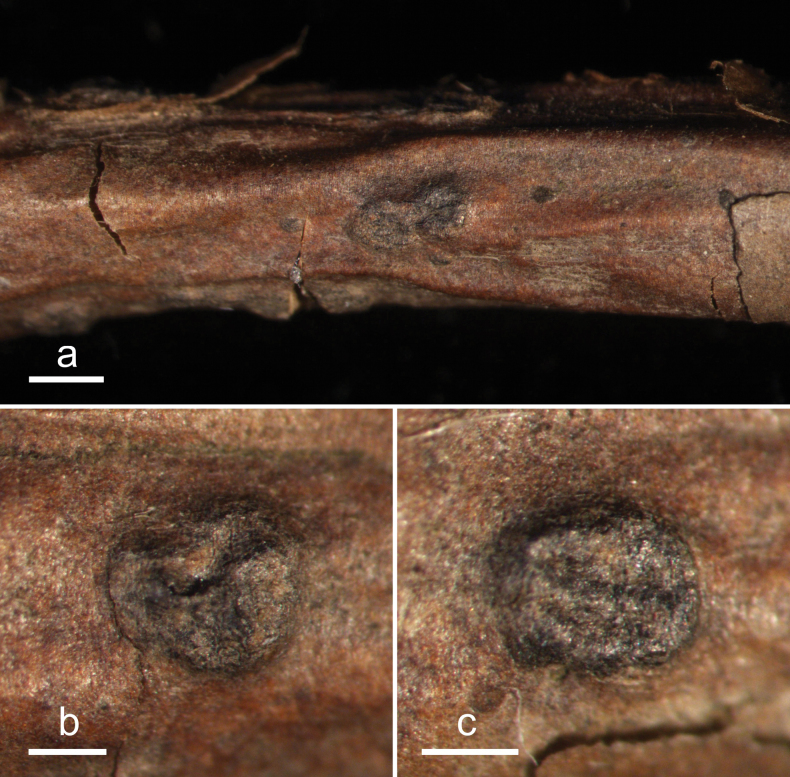
*Cryptococcomyces* sp. 1 (HOU 1835/BJTC 2021146) **a** immature ascomata on a twig of *Juniperus* sp. **b, c** immature ascomata. Scale bars: 1 mm (**a**); 500 μm (**b, c**).

#### ﻿ Hypoderma

Taxon classificationAnimaliaRhytismatalesRhytismataceae

De Not., G. bot. ital. 2(2): 13. 1847.

475B9C56-3485-5D30-B53F-92ABAC11E3E9

##### Type.

*Hypodermarubi* (Pers.) DC., in Lamarck & de Candolle, *Fl. franç*., Edn 3 (Paris) 2: 304. 1805.

##### Sexual morph.

***Ascomata*** elliptical to elongated elliptical, black, opening by a longitudinal split, usually subcuticular. ***Covering stroma*** formed by mostly dark brown, thick-walled, angular cells, in still closed ascomata with a row of thin-walled, paler cells in the inner half of the wall, along the preformed line of dehiscence. ***Basal Covering stroma*** formed prior to the development of the covering stroma or of differentiated asci or paraphyses, comprising several layers of brown, thick-walled, angular cells. ***Subhymenium*** consisting of small cells or textura porrecta. ***Paraphyses*** filiform, branched or not branched, sometimes with swollen tips, usually coiled at the tips. ***Asci*** ripening sequentially, cylindrical to clavate, thin-walled, J–, 8-spored. ***Ascospores*** 0–1 septate, variable in shape, hyaline, usually covered by a gelatinous sheath.

##### Asexual morph.

***Conidiomata***, when present, small, subcuticular, circular in outline when viewed from above, lenticular when viewed in vertical section. ***Upper wall*** present or not. ***Lower wall*** lined with cylindric, solitary conidiogenous cells which proliferate either sympodially or percurrently. ***Conidia*** small, not septate, hyaline (Description based on Johnston 1990a).

##### Notes.

*Hypoderma* is a large genus in *Rhytismataceae* and shares many morphological features with *Lophodermium*. De Notaris (1847) distinguished *Hypoderma* and *Lophodermium* by the shape of the ascospores, placing species with cylindrical to elliptical spores in the former, and those with filiform spores in the latter genus. Johnston (1990a) redefined *Hypoderma* primarily based on features associated with the pattern of development in the sterile tissues of the ascomata rather than the shape of ascospores. Two species of *Hypoderma* are known growing on twigs of conifers, *Hypodermaabietinum* Ellis & Everh. and *H.shimanense* Y. Suto.

#### ﻿ Hypoderma cunninghamiicola

Taxon classificationAnimaliaRhytismatales

Peng Zhang & S.J. Wang sp. nov.

4B515709-4821-5DEE-9470-BE2B7FDE569B

856614

Figs 13 , 14

##### Etymology.

Referring to the host genus *Cunninghamia*.

##### Diagnosis.

This new species is similar to *Hypodermarubi* (Pers.) De Not., but differs by a thicker covering stroma and a basal Covering stroma, longer asci and longer ascospores.

##### Type.

CHINA, Anhui Province, Yaoluoping National Nature Reserve, Dachuanling, alt. ca 800 m, on one year old twigs or needles of *Cunninghamialanceolata* (*Cupressaceae*), 9 Sept. 2023, S.J. Wang, L. Zhen, X.Y. Li, and Y.L. Li, L4648 (AAUF 70756, holotype).

##### Sexual morph.

***Ascomata*** on one-year twigs and needles, scattered, sometimes 2 to 3 clustered. In surface view, ascomata elliptical to elongated-elliptical, 750–1950 × 275–375 µm, dark brown (#2b180b) to black (#000000), sometimes dark brown in the center, gray (#787870) at the edge, shiny, with a conspicuous black perimeter line, opening by a single longitudinal split. ***Lips*** well developed, yellow-brown (#f2d98d). In median vertical section, ascomata subcuticular. ***Covering stroma*** 40–60 μm thick near the center of ascomata, gradually thinner towards the edge, connecting to the basal Covering stroma, consisting of an outer layer of host tissue, and an inner layer of dark brown, thick-walled textura angularis. ***Lips cells*** 3–4-septate, thin-walled, cylindrical, radially arranged, 20–28 × 3–4 µm, hyaline to yellow-brown. ***Basal Covering stroma*** 7–15 µm thick, consisting of carbonized, thick-walled, angular cells. Triangular space in vertical section between the covering stroma and the basal Covering stroma at the margin of the ascoma is filled with hyaline, thin-walled, angular cells. ***Subhymenium*** 5–15 µm thick, consisting of hyaline textura porrecta. ***Paraphyses*** filiform, sometimes branched, not swollen or slightly swollen at tips, 140–160 × 1–1.2 µm, covered by a thin gelatinous sheath. ***Asci*** ripening sequentially, clavate, apex subtruncate or rounded, 70–130 × 12–15 µm, thin-walled, J–, 8-spored. ***Ascospores*** aseptate, cylindrical, elliptical or cylindrical-clavate, 14–22 × 3.5–5 μm, hyaline, covered by a ca. 1 μm thick gelatinous sheath.

##### Asexual morph.

***Conidiomata*** rounded or elliptical, brown (#48240a), slightly raising the substrate surface, opening by 1–2 ostioles. In median vertical section, conidiomata subcuticular. ***Upper wall*** absent. ***Basal wall*** poorly developed, black-brown, 3–5 μm thick, consisting of angular cells. ***Conidia*** and ***zone lines*** not seen.

##### Additional specimens examined.

CHINA, Anhui Province, Yaoluoping National Nature Reserve, Dachuanling, alt. ca. 800 m, on one year old twigs or needles of *Cunninghamialanceolata* (*Cupressaceae*), 9 Sept. 2023, S.J. Wang, L. Zhen, X.Y. Li, and Y.L. Li, L4647 (AAUF 70755).

##### Distribution.

Known only from Anhui Province, China.

##### Notes.

Phylogenetic analysis reveals that, with the exception of *H.berberidis*, *H.minteri*, *H.caricis*, and *H.junipericola*, other species of *Hypoderma* are divided into two distant clades. The sequences of the new species, along with several others, form a highly supported clade (MLB = 100%, MPB = 81%, PP = 0.99; Clade 9, Fig. 1). Morphologically, *H.cunninghamiicola* is similar to the type of *Hypoderma*, *H.rubi*, but the latter has a thicker covering stroma (50–80 μm), a thicker basal Covering stroma (10–15 μm), longer asci (110–160 μm) and longer ascospores (15–28 μm) (Johnston 1990a). The host range of *H.rubi* is broad, and it has been reported on both broadleaved plants and conifers (e.g. *Cunninghamialanceolata*). Considering its saprobic nature, these reports are plausible. However, further research incorporating molecular data is required to confirm whether these occurrences truly represent *H.rubi* or distinct, cryptic species.

**Figure 13.**
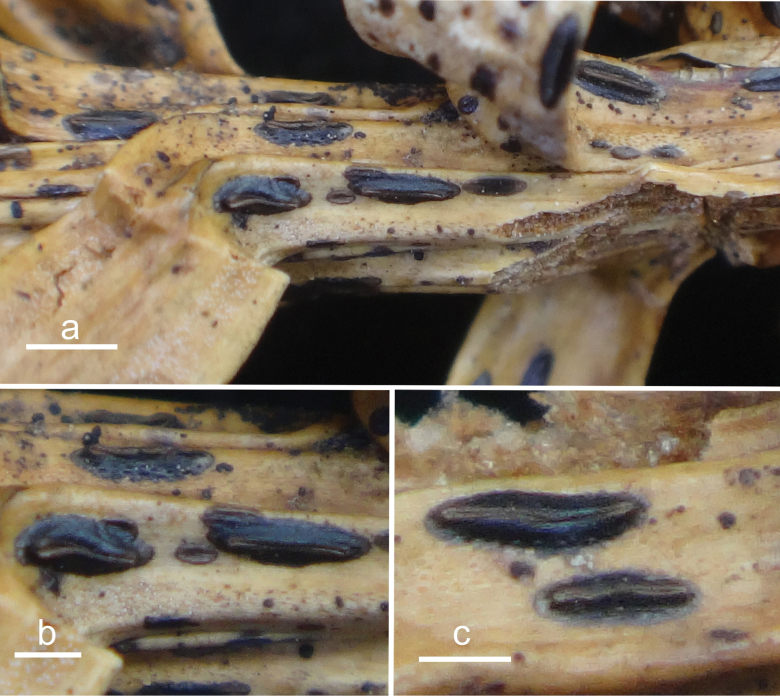
*Hypodermacunninghamiicola* (L4648/AAUF70756, holotype) **a** ascomata on a twig of *Cunninghamialanceolata***b, c** mature ascomata. Scale bars: 1000 μm (**a**); 500 μm (**b, c**).

**Figure 14.**
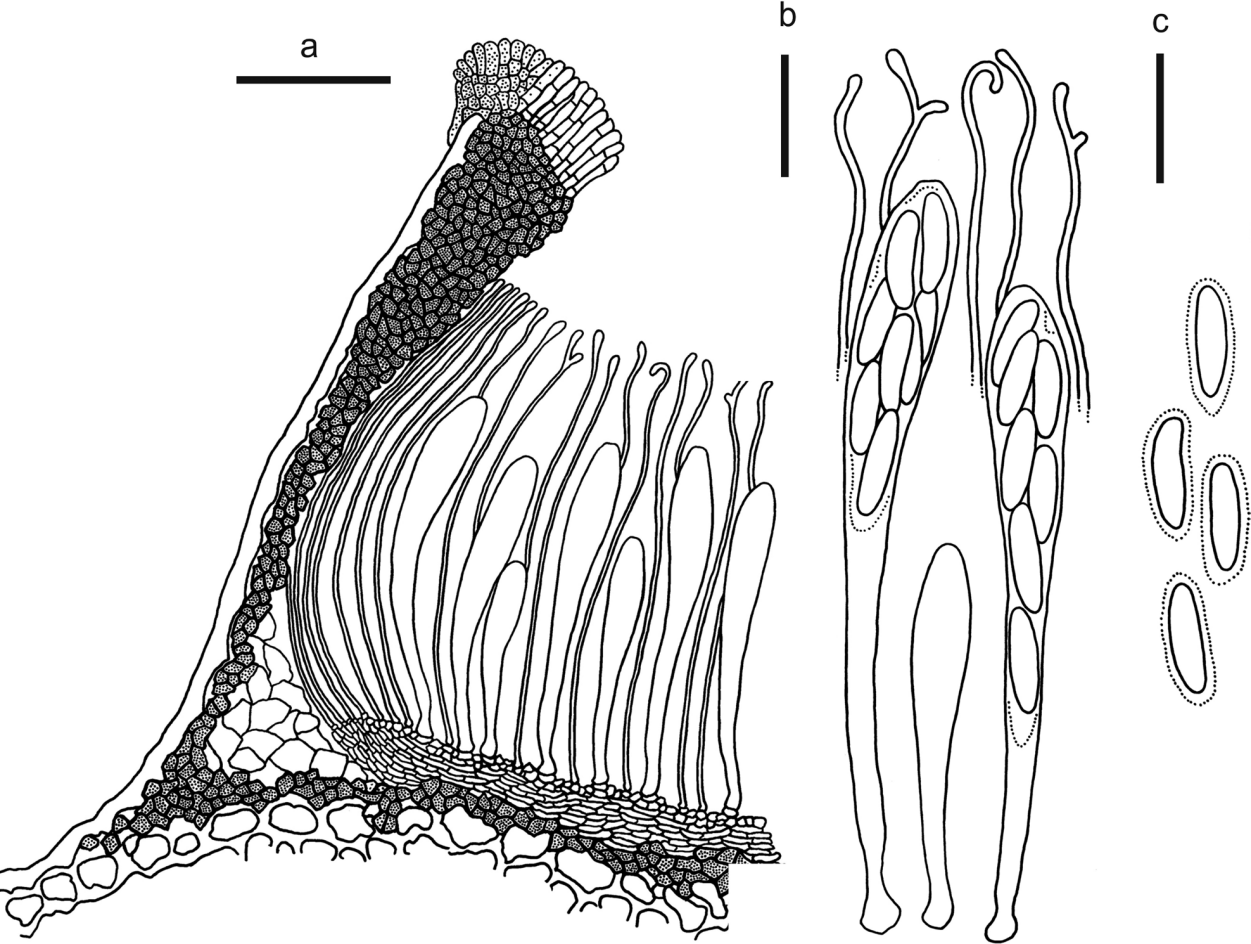
*Hypodermacunninghamiicola* on *Cunninghamialanceolata* (L4648/AAUF70756, holotype) **a** part of an ascoma in vertical section **b** paraphyses, mature asci with ascospores, and immature ascus **c** liberated ascospores. Scale bars: 50 μm (**a**); 20 μm (**b, c**).

#### ﻿ Hypohelion

Taxon classificationAnimaliaRhytismatalesRhytismataceae

P.R. Johnst., Mycotaxon 39: 221. 1990.

14036C6C-CCBA-5027-A5CD-2A1C46794908

##### Type species.

*Hypohelionscirpinum* (DC.) P.R. Johnst., Mycotaxon 39: 221. 1990.

##### Sexual morph.

***Ascomata*** scattered to clustered, subcircular, elliptical or irregular, black, opening by an irregular longitudinal split. ***Covering stroma*** formed by dark brown (#2b180b) to black (#000000), thick-walled angular cells, more or less uniform in thickness. ***Basal Covering stroma*** absent or formed by carbonized, thick-walled angular cells. ***Subhymenium*** consisting of textura intricata. ***Paraphyses*** filiform, not branched, with swollen tips. ***Asci*** ripening sequentially, clavate, thin-walled, J–, 8-spored. ***Ascospores*** 0–1 septate, cylindrical, elliptical or slightly clavate, hyaline, covered by a gelatinous sheath.

##### Asexual morph.

***Conidiomata***, when present, round, dark brown (#2b180b) to black (#000000), with a central ostiole. ***Conidiogenous cells*** cylindrical, tapering towards apex, hyaline. ***Conidia*** simple, oblong-elliptical to cylindrical, hyaline (Description based on Johnston 1990b).

##### Notes.

*Hypohelion* was established by Johnston (1990b) to accommodate two species, *Hy.scirpinum* and *Hy.parvum* P.R. Johnston. Both species are found on members of *Cyperaceae* Juss. growing in swampy habitats. *Hypohelion* can be distinguished from other genera by subcuticular ascomata; covering stroma consisting by dark brown, thick-walled, angular cells, more or less uniform in thickness; edge of the ascomata opening without differentiated cells; lacking a basal Covering stroma; and paraphyses with swollen apex (Johnston 1990b). *Hypoheliondurum* Y.R. Lin, C.L. Hou & S.J. Wang (Lin et al. 2004) and *Hy.anhuiense* Shuang Wang & C.L. Hou (Wang et al. 2014a) were added to this genus subsequently. However, *Hy.durum* differs from other *Hypohelion* species based on molecular phylogenetic data, morphology, and substrate. *Hypoheliondurum* should therefore be excluded from the genus *Hypohelion* (Wang et al. 2014a).

#### ﻿ Hypohelion shennongjianum

Taxon classificationAnimaliaRhytismatalesRhytismataceae

Lan Zhuo & C.L. Hou sp. nov.

B98F76D8-B5D4-51C1-8C6C-DD9A6AFFE617

856615

Figs 15 , 16

##### Etymology.

Referring to the name of the location in the Shennongjia forestry region where the type specimen was collected.

##### Diagnosis.

This new species is similar to *Coccomycesanhuiensis* T. Lv & C.L. Hou, but differs by intraepidermal ascomata and ascospores with an apical gelatinous cap, having 4–6 short filament-like appendage on the gelatinous cap.

##### Type.

CHINA, Hubei Province, Shennongjia forestry region, Muyuzhen, 31.4200°N, 110.4030°E, alt. ca. 1560 m, on twigs of *Cunninghamialanceolata* (Lamb.) Hook. (*Cupressaceae*), 20 Jul. 2018, C.L. Hou & T. Lv. HOU 1342A (BJTC 2018037, holotype).

##### Sexual morph.

***Ascomata*** on young and dead twigs, scattered, not associated with pale areas. In surface view, ascomata wide elliptical to irregular elliptical, 650–950 × 350–500 µm, black (#000000), or black in the center and gray (#787870) towards the margin, shiny, with black perimeter line, opening by an irregular, longitudinal, 3-teethed split. ***Lips*** absent. In median vertical section, ascomata intraepidermal. ***Covering stroma*** 30–35 μm thick near the center of ascomata, extending to the basal Covering stroma, consisting of an outer layer of host cuticle and an inner layer of carbonized, thick-walled angular cells. ***Basal Covering stroma*** 15–20 µm thick, consisting of carbonized, thick-walled, angular cells. Triangular space visible in vertical section between the covering stroma and the basal Covering stroma at margin of ascoma filled with thin-walled angular, globose cells. ***Subhymenium*** 10–15 µm thick, consisting of hyaline textura intricata. ***Excipulum*** well-developed, formed by marginal paraphyses. ***Paraphyses*** aseptate, filiform, not branched, not swollen at tips, 120–140 × 1–2 µm. ***Asci*** ripening sequentially, clavate, apex bluntly pointed, 70–90 × 6–10 µm, thin-walled, J–, 8-spored. ***Ascospores*** aseptate, clavate, 35–45 × 2–3 μm, hyaline, multi-guttulate when immature, covered by thin gelatinous sheaths, with a gelatinous cap at the tip of mature ascospores, with 4–6 short filament-like appendages on each gelatinous cap.

##### Asexual morph.

see Notes. ***Zone lines*** infrequent, diffuse, black.

##### Distribution.

Known only from Hubei Province, China.

##### Notes.

Based on the phylogenetic analysis, *Hy.shennongjianum* clusters with the type of the genus *Hypohelion*, *Hy.scirpinum*, as well as *Hy.anhuiense*, with weak support (Clade 5, Fig. 1). Morphologically, *Hy.shennongjianum* shares clavate ascospores with other species of *Hypohelion*, but the shape of its ascomata is distinct from other *Hypohelion* species and resembles that of *Coccomycesanhuiensis*. However, unlike *Co.anhuiensis*, *Hy.shennongjianum* has intraepidermal ascomata and ascospores carry a gelatinous cap at the tops, bearing 4–6 short filament-like appendages on the gelatinous cap. Considering both its phylogenetic position and morphological characteristics, this new species should be placed in the genus *Hypohelion*.

A probable asexual morph was observed in association with *Hy.shennongjianum*. The conidiomata are scattered, occasionally confluent, elliptical, reddish brown (#68271a), and 100–200 × 200–350 µm in size. In vertical section, the conidiomata are intraepidermal, 80–100 µm deep, with an upper layer 25–30 µm thick, composed of host cuticle and carbonized angular to globose cells, and a basal layer 15–18 µm thick, consisting of similar carbonized cells. Conidiogenous cells and conidia were not observed. Sequencing of the conidiomata was attempted but unsuccessful, so molecular data does not currently support the connection.

**Figure 15.**
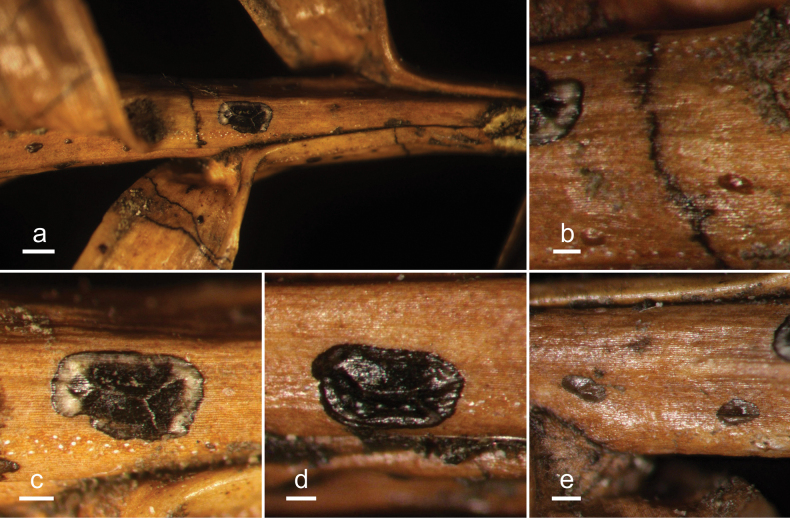
*Hypohelionshennongjianum* (HOU 1342A/BJTC 2018037, holotype) **a** ascomata on a twig of *Cunninghamialanceolata***b** zone line **c, d** mature ascomata **e** conidiomata. Scale bars: 500 μm (**a**); 100 μm (**b**); 200 μm (**c–e**).

**Figure 16.**
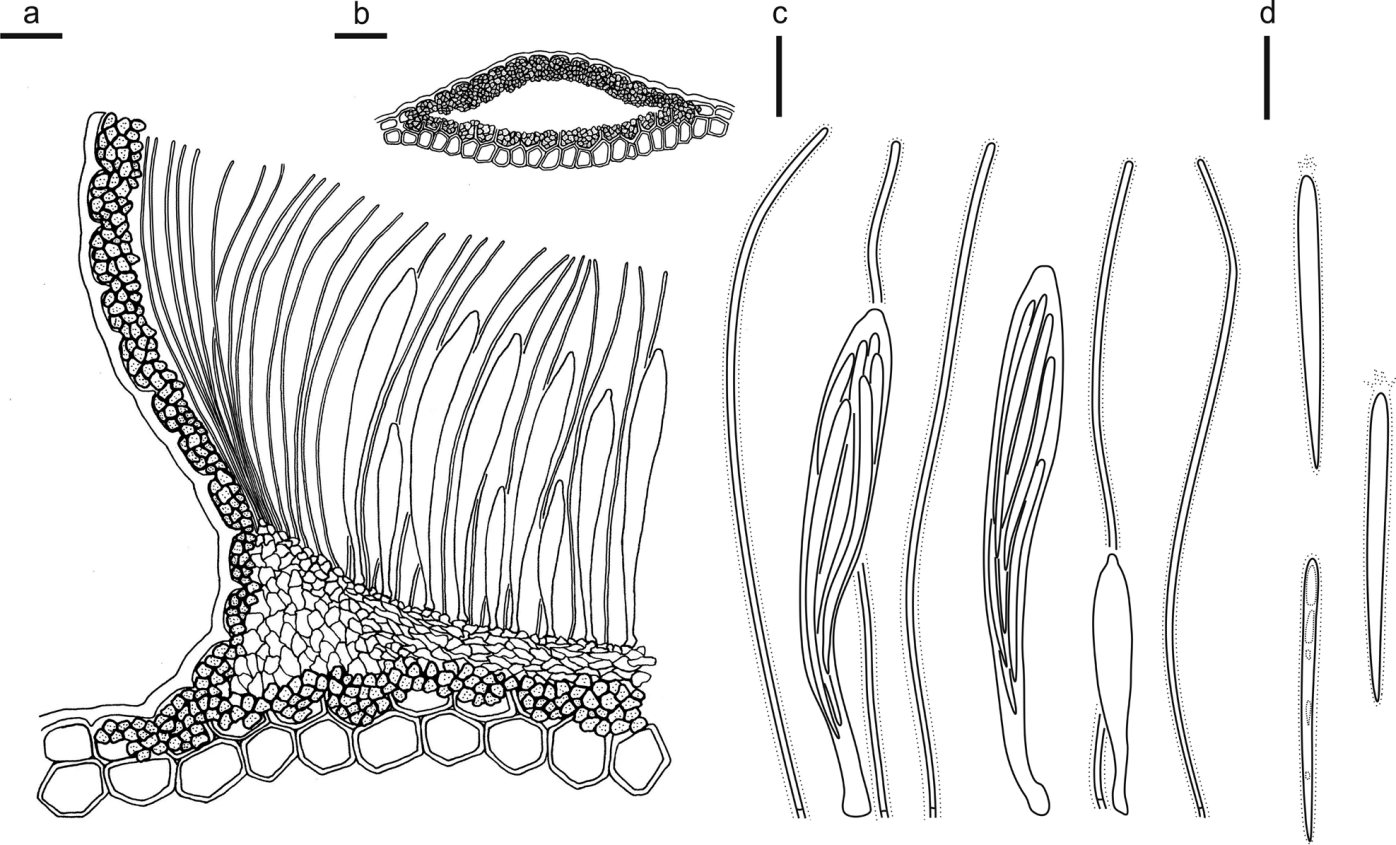
*Hypohelionshennongjianum* on *Cunninghamialanceolata* (HOU 1342A /BJTC 2018037, holotype) **a** part of an ascoma in vertical section **b** conidioma in vertical section **c** paraphyses, mature asci with ascospores, and immature ascus **d** liberated ascospores with apical gelatinous cap. Scale bars: 50 μm (**a, b**); 10 μm (**c**); 20 μm (**d**).

#### ﻿ Labivalidus

Taxon classificationAnimaliaRhytismatalesRhytismataceae

Lan Zhuo & C.L. Hou gen. nov.

42792239-3CA1-5FA4-8231-74F042F6D279

856616

##### Etymology.

*Labium* (Latin) = lip, *validus* (Latin) = strong, referring to the well-developed lips of ascomata.

##### Diagnosis.

This new genus is similar to *Hypoderma*, but differs from *Hypoderma* by broadly elliptical ascomata and filiform ascospores.

##### Type.

*Labivaliduscunninghamiae* Lan Zhuo & C.L. Hou, described below.

##### Sexual morph.

***Ascomata*** often scattered, elliptical to broadly elliptical, black (#000000), opening by a single longitudinal split. ***Lips*** well-developed, creamy white(#fffeea). Ascomata intraepidermal. ***Covering stroma*** consisting of carbonized, thick-walled, angular to globose cells. ***Basal Covering stroma*** moderately to well developed, consisting of carbonized, thick-walled, angular to globose cells. ***Internal matrix of Covering stroma*** absent. ***Paraphyses*** filiform, not branched, not swollen or swollen at tips. ***Asci*** ripening sequentially, cylindrical, rostrate at apex at maturity, thin-walled, J–, 8-spored. ***Ascospores*** aseptate, filiform, hyaline, covered by a gelatinous sheath.

##### Asexual morph.

***Conidiomata*** and ***zone lines*** not seen.

##### Notes.

In the context of the present study, molecular data for the species described as *Lophodermiumjianchuanense* have been obtained. Phylogenetic analyses shows that the molecular sequences of *Lo.jianchuanense* and the new species *Labivaliduscunninghamiae* (described below) form a distinct clade (Clade 10, Fig. 1). Both species are distantly related to the type species of the genus *Lophodermium*, *Lo.arundinaceum* (Schrad.) Chevall., as well as the species of *Lophodermium* on needles of conifers, indicating that *La.cunninghamiae* and *Lo.jianchuanense* should not be classified in the genus *Lophodermium*.

Morphologically, the ascomata of both *Lo.jianchuanense* and *La.cunninghamiae* have conspicuous, creamy-colored lips, which are similar to some species of the genus *Hypoderma*. However, species in the genus *Hypoderma* s. str. are usually saprotrophic, with a wide host range, their ascomata are often elliptical to elongate in shape, and ascospores elliptical to cylindrical (De Notaris 1847; Powell 1974; Cannon and Minter 1986; Johnston 1990a), whereas *Lo.jianchuanense* and *La.cunninghamiae* have a narrow host range, known only from *Cupressaceae*, have broadly elliptical ascomata and filiform ascospores. Furthermore, *Lo.jianchuanense* and *La.cunninghamiae* are also distant from the type of *Hypoderma* in the phylogenetic tree, suggesting that they should not be classified in *Hypoderma*.

Considering the phylogenetic and morphological evidence, it is necessary to establish a new genus to accommodate *Lo.jianchuanense* and *La.cunninghamiae*. *Lophodermiumjianchuanense* is transferred to *Labivalidus* as a new combination, *La.jianchuanensis*.

#### ﻿ Labivalidus cunninghamiae

Taxon classificationAnimaliaRhytismatalesRhytismataceae

Lan Zhuo & C.L. Hou sp. nov.

E194AAC9-406A-5144-8A79-BAE4AAE612BC

856617

Figs 17 , 18

##### Etymology.

Referring to the host genus *Cunninghamia*.

##### Diagnosis.

This new species is similar to *Labivalidusjianchuanensis*, but differs in having larger ascomata, larger ascospores, and paraphyses that are conspicuously swollen at their tips.

##### Type.

CHINA, Anhui Province, Anqing, Yuexi County, Wenao Forest Farm, 30.8050°N, 116.0885°E, alt. ca. 1020 m, on twigs of *Cunninghamialanceolata* (*Cupressaceae*), 13 Apr. 2024, C.L. Hou, L. Zhuo, and X.N. Sui, HOU 2173A (BJTC 2024033, holotype).

##### Sexual morph.

***Ascomata*** on young dead twigs, scattered, sometimes 2–3 clustered. In surface view, ascomata elliptical to broadly elliptical, 900–1600 × 480–880 μm, black (#000000), opening by a longitudinal split. ***Lips*** well developed, creamy white (#fffeea). In median vertical section, ascomata intraepidermal. ***Covering stroma*** 50–90 μm thick near the center of the ascomata, extending to the basal Covering stroma, consisting of an outer layer of host cuticle, and an inner layer of carbonized, thick-walled, angular to globose cells. ***Lip cells*** septate, cylindrical, 6–10 × 2–4 μm, hyaline. ***Basal Covering stroma*** 20–30 µm thick, consisting of carbonized, thick-walled, angular to globose cells. A space triangular in vertical section between the covering stroma and the basal Covering stroma at the margin of the ascoma is filled with thin-walled, hyaline cells. ***Subhymenium*** 20–30 µm thick, consisting of textura intricata. ***Paraphyses*** filiform, not branched, conspicuously swollen to 2–3 μm diam. at their tips, 150–195 × 1 µm, covered by a thin gelatinous sheath. ***Asci*** ripening sequentially, cylindrical, with rostrate apex, 130–180 × 13–15 μm, thin-walled, J–, 8-spored. ***Ascospores*** aseptate, filiform, 100–125 × 3–4 μm, hyaline, covered by a 1–2 μm thick, gelatinous sheath.

##### Asexual morph.

***Conidiomata*** and ***zone lines*** not seen.

##### Additional specimens examined.

CHINA, Anhui Province, Anqing, Yuexi County, Yaoluoping Nature Reserve, 30.8050°N, 116.0885°E, alt. ca. 1020 m, on twigs of *Cunninghamialanceolata* (*Cupressaceae*), 13 Apr. 2024, C.L. Hou, L. Zhuo, and X.N. Sui, HOU 2166 (BJTC 2024026).

##### Distribution.

Known only from Anhui Province, China.

##### Notes.

In the phylogenetic tree, the molecular sequences of *Labivaliduscunninghamiae* form a clade sister to *La.jianchuanensis*. *Labivalidusjianchuanensis* differs from *La.cunninghamiae* by having smaller ascomata (500–750(–920) µm × 400–520 µm), smaller ascospores (65–90 µm × 1–1.5(–2) µm), and tips of paraphyses that are not swollen.

**Figure 17.**
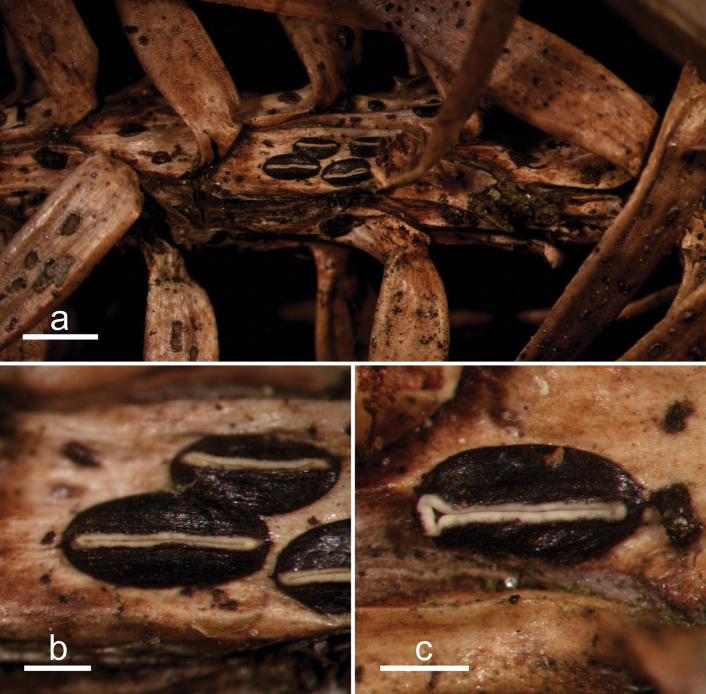
*Labivaliduscunninghamiae* (HOU 2173A/BJTC 2024033, holotype) **a** ascomata on a twig of *Cunninghamialanceolata***b, c** Mature ascomata. Scale bars: 2 mm (**a**); 500 μm (**b, c**).

**Figure 18.**
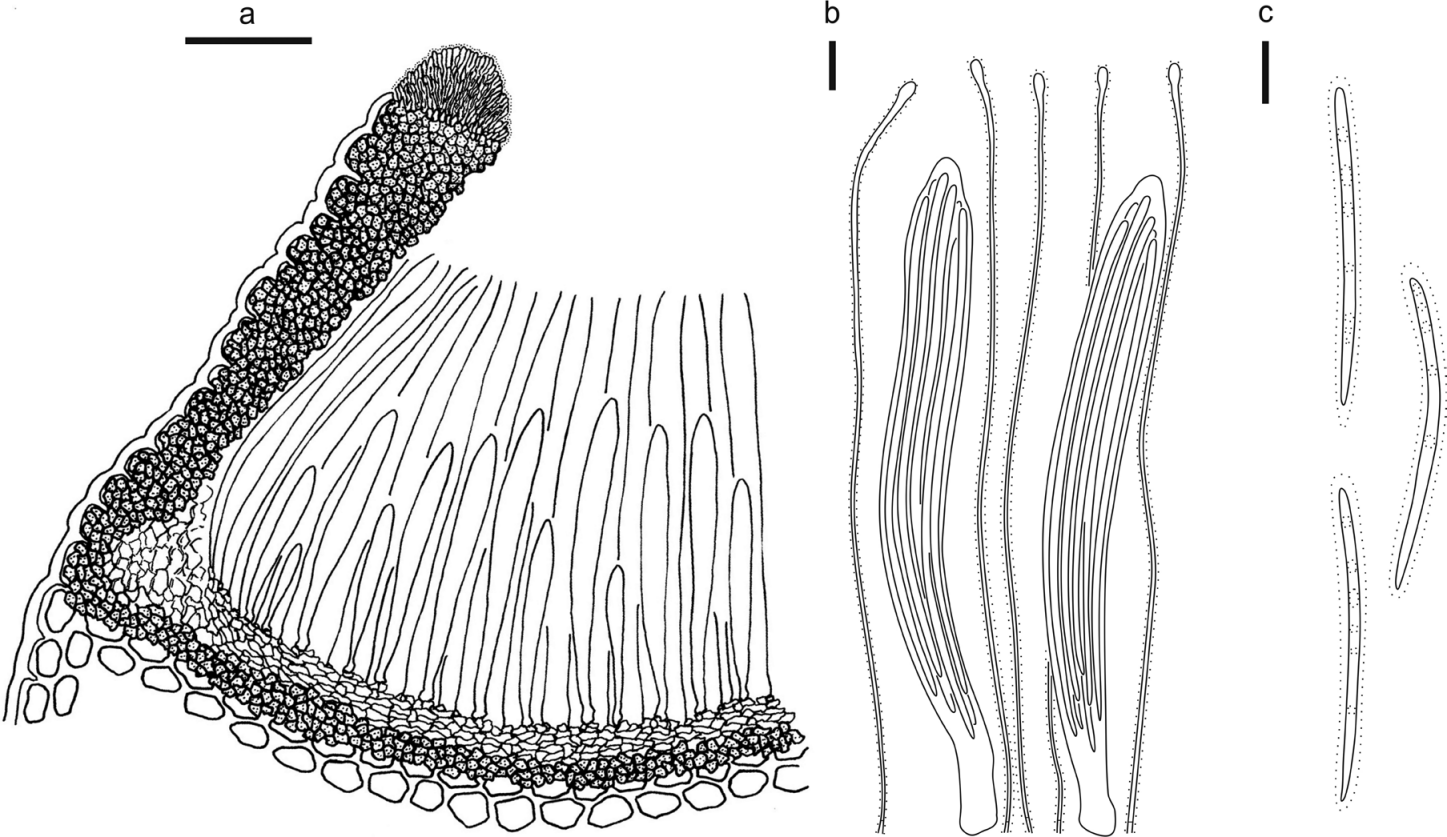
*Labivaliduscunninghamiae* on *Cunninghamialanceolata* (HOU 2173A /BJTC 2024033, holotype) **a** part of an ascoma in vertical section, lips well developed **b** paraphyses, mature asci with ascospores, and immature ascus **c** liberated ascospores. Scale bars: 100 μm (**a**); 10 μm (**b**); 20 μm (**c**).

#### ﻿ Labivalidus jianchuanensis

Taxon classificationAnimaliaRhytismatalesRhytismataceae

(C.L. Hou & M. Piepenbr.) Lan Zhuo & C.L. Hou comb. nov.

6ECC7225-E689-535A-B8A6-DB49603DD48C

856618

Fig. 19

Lophodermium jianchuanense C.L. Hou & M. Piepenbr., Can. J. Bot. 83(1): 40. 2005. Basionym.

##### Type.

CHINA, Yunnan Province, Jianchuan, Laojunshan, alt. ca. 3400 m, on *Juniperusformosana* Hayata (*Cupressaceae*), 25 Jul. 2001, C.L. Hou, M. Piepenbring, Z.L. Yang, & R. Kirschner 104 (HMAS, holotype).

##### Specimens examined.

CHINA, Yunnan Province, Lijiang, Laojunshan, 26.6319°N, 99.7252°E, alt. ca. 3855 m, on needles and twigs of *Juniperussquamata* (*Cupressaceae*), 21 Jun. 2021, C.L. Hou, M.J. Guo, and H. Zhou, HOU 1781B (BJTC 2021092); 26.6314°N, 99.7247°E, alt. ca. 3850 m, on needles and twigs of *J.squamata*, 21 Jun. 2021, C.L. Hou, M.J. Guo, and H. Zhou, HOU 1815A (BJTC 2021126); 26.6425°N, 99.7678°E, alt. ca. 3500 m, on needles and twigs of *J.squamata*, 17 Aug. 2023, C.L. Hou, L. Zhuo, and S.Y. Zhao, HOU 2023 (BJTC 2023153); 26.6425°N, 99.7678°E, alt. ca. 3500 m, on needles and twigs of *J.squamata*, 17 Aug. 2023, C.L. Hou, L. Zhuo, and S.Y. Zhao, HOU 2024 (BJTC 2023154).

##### Notes.

Hou et al. (2005) described *Lophodermiumjianchuanense* on *Juniperusformosana* in the genus *Lophodermium*. In the phylogenetic tree, the molecular sequences of *Lo.jianchuanense* form a clade sister to *La.cunninghamiae* and distant from *Lo.arundinaceum*, the type species of *Lophodermium*. Based on this phylogenetic position and morphological similarity, we transfer *Lo.jianchuanense* to the genus *Labivalidus*, the epithet has been adjusted to agree with the gender of the new genus name.

**Figure 19.**
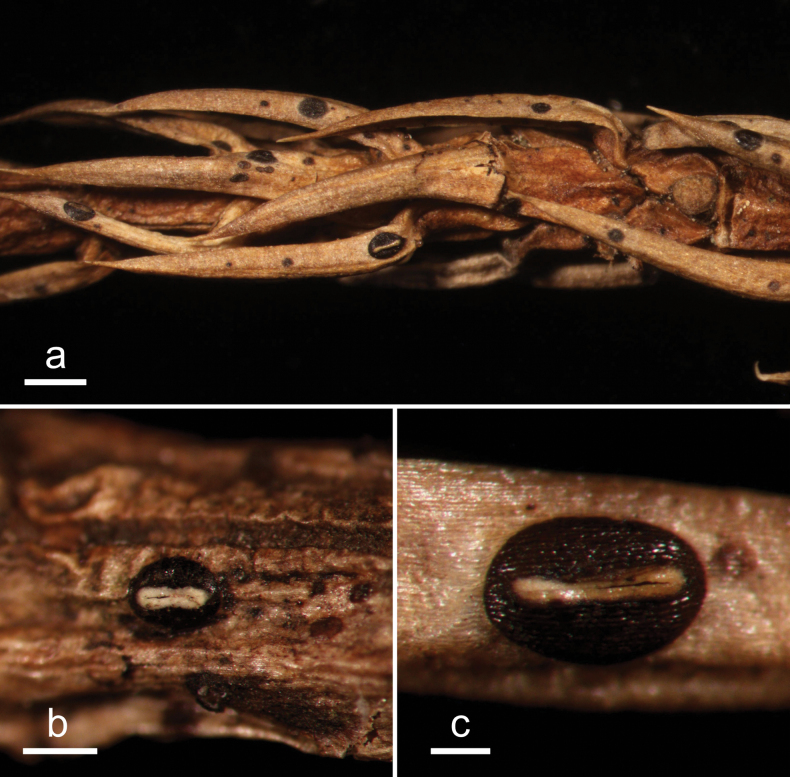
*Labivalidusjianchuanensis* (HOU 1781B/BJTC 2021092) **a** ascomata on needles of *Juniperussquamata***b, c** mature ascomata on a twig or a needle of *Juniperussquamata*. Scale bars: 1 mm (**a**); 500 μm (**b**); 200 μm (**c**).

#### ﻿ Neotherrya

Taxon classificationAnimaliaRhytismatalesRhytismataceae

Lan Zhuo & C.L. Hou gen. nov.

836326D9-F636-5FEA-8EB9-1288F840F481

856619

##### Etymology.

Referring to the morphologically similar genus *Therrya*.

##### Diagnosis.

This new genus is similar to *Therrya*, but *Neotherrya* differs in having well-developed excipulum formed by marginal paraphyses, internal matrix of Covering stroma consisting of hyaline hyphae, and filiform, fusiform, or cylindrical ascospores.

##### Type.

*Neotherryacircinata* Lan Zhuo & C.L. Hou, described below.

##### Sexual morph.

***Ascomata*** on twigs of conifers, scattered to clustered, circular, sessile, usually black, opening by irregular splits. ***Covering stroma*** formed by dark brown (#2b180b) to black (#000000), thick-walled angular cells. ***Basal Covering stroma*** usually present, consisting of carbonized, thick-walled, angular cells. ***Internal matrix of Covering stroma*** usually well developed, consisting of hyaline hyphae, filled or not filled with crystals. ***Subhymenium*** consisting of textura intricata or hyaline cells. ***Paraphyses*** filiform, not branched, with swollen tips. ***Asci*** ripening sequentially, clavate, thin-walled, J–, 8-spored or 4-spored. ***Ascospores*** mostly septate, filiform, fusiform or cylindrical, hyaline, covered or not covered by a gelatinous sheath.

##### Notes.

Some of the morphological characteristics of members in *Neotherrya* are similar to those in *Therrya*, but species of *Neotherrya* differs by a well-developed excipulum formed by marginal paraphyses, an internal matrix of Covering stroma consisting of hyaline hyphae and cylindrical ascospores. Phylogenetic analyses shows that sequences of *Neotherrya* and *Therrya* form two separate clades. Sequences of *Neotherrya* (Clade 6, Fig. 1) are distant from the type of *Therrya*, *Th.pini* (Alb. & Schwein.) Höhn. Based on differences of morphological features and the position in the phylogenetic tree, we herein establish a new genus to accommodate four new species and one new combination described below.

#### ﻿ Neotherrya abieticola

Taxon classificationAnimaliaRhytismatalesRhytismataceae

(C.L. Hou & M. Piepenbr.) Lan Zhuo & C.L. Hou comb. nov.

3EE1E62D-2725-5B40-9073-9AF4671EE15F

856622

Therrya abieticola C.L. Hou & M. Piepenbr., Mycotaxon 102: 168. 2007. Basionym.

##### Type.

CHINA, Yunnan Province, Laojunshan, alt. ca. 2400 m, on twigs of *Abies* sp. (*Pinaceae*), 25 Jul. 2001, C.L. Hou, M. Piepenbring, R. Kirschner, and Z.L. Yang 103 (AAUF 90036).

##### Specimen examined.

CHINA, Yunnan Province, Lijiang, Laojunshan, alt. ca. 3500 m, on twigs of *Abies* sp. (*Pinaceae*), 11 Jul. 2007, C.L. Hou, HOU 447.

##### Notes.

Hou and Piepenbring (2007) described *Therryaabieticola* on *Abies* sp. in the genus *Therrya* Sacc. because the shapes of asci, ascospores, and paraphyses of *Th.abieticola* are similar to those of the type *Th.pini* (Alb. & Schwein.) Höhn. (Reid and Cain 1961; Hou and Piepenbring 2007). However, Hou and Piepenbring (2009) mentioned that *Th.abieticola* differs from the known species of *Therrya* by having aseptate ascospores.

Our phylogenetic analysis shows that sequences of *Th.abieticola* form a well-supported clade (MLB = 100%, MPB = 91%, PP = 1.00) together with sequences of *N.nematoidea* and are distantly related to type species of *Therrya*, *Th.pini*. Based on morphological characteristics and phylogenetic analyses, *Th.abieticola* is transferred here to the new genus *Neotherrya*. For a detailed description of this species see Hou and Piepenbring (2007).

#### ﻿ Neotherrya catilliformis

Taxon classificationAnimaliaRhytismatalesRhytismataceae

Lan Zhuo & C.L. Hou sp. nov.

E2BB60C2-5796-5669-9081-68B1DAFCD87E

856621

Figs 20 , 21

##### Etymology.

*Catilliformis* (Latin) = plate-shaped, referring to the shape of ascomata.

##### Diagnosis.

This new species is distinguished from other *Neotherrya* species by lacking an internal matrix of the Covering stroma.

##### Type.

CHINA, Yunnan Province, Lijiang, Laojunshan, 26.6323°N, 99.7252°E, alt. ca. 3880 m, on twigs of *Abiesgeorgei* (*Pinaceae*), 16 Jul. 2020, C.L. Hou, M.J. Guo, and Q.T. Wang, HOU 1608A (BJTC 2020049, holotype).

##### Sexual morph.

***Ascomata*** on twigs, slightly erumpent from the bark, scattered or aggregated, not associated with pale areas. In surface view, ascomata round to irregular, 550–950 × 350–750 µm, black (#000000), opening by irregular splits to expose an orange (#ffa500) hymenium. ***Lips*** absent. In median vertical section, ***covering stroma*** 20–40 μm thick near the center of ascomata, extending to the basal Covering stroma, consisting of an outer layer of carbonized, angular to globose cells and an inner layer of hyaline, angular to globose cells close to the opening. ***Excipulum*** well-developed, formed by marginal paraphyses. ***Basal Covering stroma*** 15–25 µm thick, easily separable from the hymenium layer when observed under the microscope, consisting of carbonized, thick-walled, angular cells. ***Internal matrix of Covering stroma*** absent. ***Subhymenium*** 25–30 µm thick, consisting of hyaline, globose cells. ***Paraphyses*** aseptate, filiform, not branched, swollen to 2–3 µm at tips, 130–150 × 1–2 µm, covered by a thin gelatinous sheath. ***Asci*** ripening sequentially, clavate, apex obtuse, 110–130 × 12–15 µm, thin-walled, J–, 4-spored. ***Ascospores*** 1–8-septate, cylindrical, 12–15 × 6–7 μm, hyaline, without gelatinous sheaths.

##### Asexual morph.

***Conidiomata*** and ***zone lines*** not seen.

##### Additional specimens examined.

CHINA, Yunnan Province, Lijiang, Laojunshan, 26.6323°N, 99.7252°E, alt. ca. 3880 m, on twigs of *Abiesgeorgei* (*Pinaceae*), 26 Jul. 2024, C.L. Hou, L. Zhuo, and X.N. Sui, HOU 2304 (BJTC 2024154); 26.6357°N, 99.7294°E, alt. ca. 3850 m, on twigs of *A.georgei*, 17 Aug. 2023, C.L. Hou, L. Zhuo, and S.Y. Zhao, HOU 2097 (BJTC 2023227).

##### Distribution.

Known only from Yunnan Province, China.

##### Notes.

In the phylogenetic tree, sequences of *N.catilliformis* form a well-supported clade (MLB = 100%, MPB = 97%, PP = 1.00) with *N.circinata*, but the latter has a well-developed internal matrix in the Covering stroma, a black epithecium formed by curled, coiled paraphyses, and 8-spored asci.

**Figure 20.**
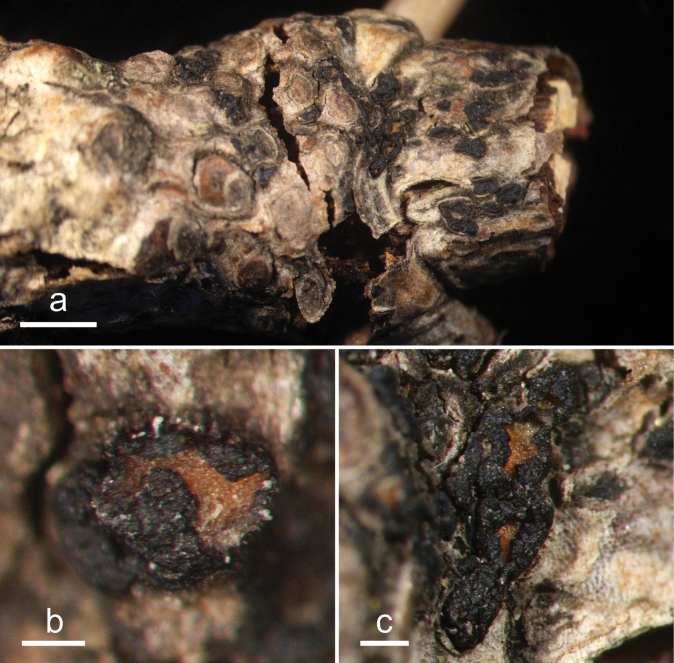
*Neotherryacatilliformis* (HOU 1608A/BJTC 2020049, holotype) **a** ascomata on a twig of *Abiesgeorgei***b, c** mature ascomata. Scale bars: 3 mm (**a**); 200 μm (**b**); 500 μm (**c**).

**Figure 21.**
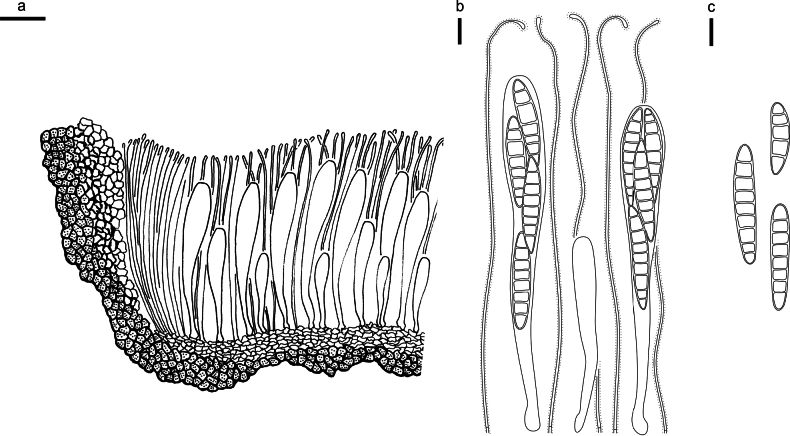
*Neotherryacatilliformis* on *Abiesgeorgei* (HOU 1608A/BJTC 2020049, holotype) **a** part of an ascoma in vertical section **b** paraphyses, mature asci with ascospores, and immature ascus **c** liberated ascospores. Scale bars: 50 μm (**a**); 10 μm (**b, c**).

#### ﻿ Neotherrya circinata

Taxon classificationAnimaliaRhytismatalesRhytismataceae

Lan Zhuo & C.L. Hou sp. nov.

D0CC132C-9A6B-5BFE-8D03-6B012184E35D

856620

Figs 22 , 23

##### Etymology.

From circinatus (Latin) = to make round, referring to the curled tips of the paraphyses.

##### Diagnosis.

This new species is similar to *Neotherryacatilliformis*, but differs by its well-developed internal matrix of the Covering stroma, curled and coiled tips of paraphyses, and 8-spored asci.

##### Type.

CHINA, Yunnan Province, Lijiang, Laojunshan, alt. ca. 3800 m, on twigs of *Abiesgeorgei* (*Pinaceae*), 25 Jun. 2011, C.L. Hou, HOU 883 (BJTC 2011041, holotype).

##### Sexual morph.

***Ascomata*** on twigs, slightly erumpent from bark, scattered, not associated with pale areas. In surface view, ascomata round to irregular, 800–1000 µm in diam., black (#000000), opening by irregular splits to expose a tangerine yellow (#ffb700) hymenium. ***Lips*** absent. In median vertical section, ***covering stroma*** 65–85 μm thick near the center of ascomata, extending to the basal Covering stroma, consisting of an outer layer of carbonized, angular cells and an inner layer of hyaline, angular cells. ***Excipulum*** well-developed, formed by marginal paraphyses. ***Basal Covering stroma*** 25–35 µm thick, consisting of an outer layer of carbonized, angular cells and an inner layer of hyaline, angular cells. ***Internal matrix of Covering stroma*** well-developed, consisting of hyaline hyphae, filled with irregular crystals. ***Subhymenium*** 10–15 µm thick, consisting of textura intricata. ***Paraphyses*** aseptate, filiform, not branched, curled and coiled, slightly swollen at their tips, forming the black epithecium, 110–140 × 2–2.5 µm. ***Asci*** ripening sequentially, clavate, apex obtuse, 75–125 × 10–17 µm, thin-walled, J–, 8-spored. ***Ascospores*** initially non-septate, with up to 7 septa when mature, cylindrical, acute at both ends, 23–38 × 2–4 μm, hyaline, covered by a 2–3 µm thick gelatinous sheath.

##### Asexual morph.

***Conidiomata*** and ***zone lines*** not seen.

##### Additional specimens examined.

CHINA, Yunnan Province, Lijiang, Laojunshan, 26.6317°N, 99.7250°E, alt. ca. 3280 m, on twigs of *Abiesgeorgei* (*Pinaceae*), 17 Jul. 2021, C.L. Hou, M.J. Guo, and Q.T. Wang, HOU 1587 (BJTC 2020026); 26.6308°N, 99.7165°E, alt. ca. 4000 m, on twigs of *A.georgei*, 21 Jun. 2021, C.L. Hou, M.J. Guo, and H. Zhou, HOU 1824 (BJTC 2021135).

##### Distribution.

Known only from Yunnan Province, China.

##### Notes.

Phylogenetically, sequences of *Neotherryacircinata* form a well-supported clade (MLB = 100%, MPB = 97%, PP = 1.00) with *N.catilliformis*, but the latter lacks an internal matrix of the Covering stroma and has 4-spored asci. Morphologically, the new species is similar to *Neotherryanematoidea*, but *N.nematoidea* has well- developed periphysoids, and nematode-like ascospores.

**Figure 22.**
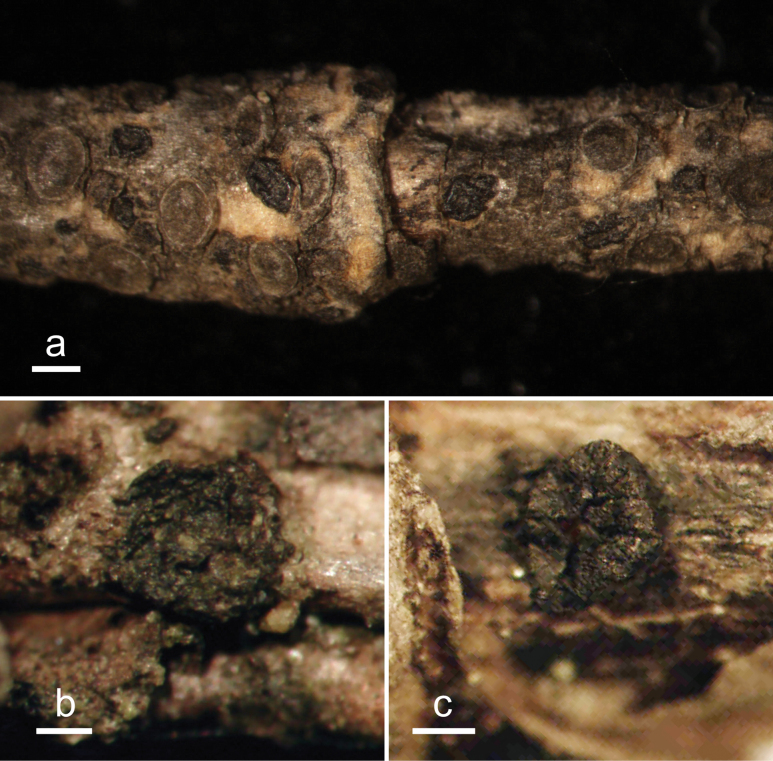
*Neotherryacircinata* (HOU 883/BJTC 2011041, holotype) **a** ascomata on a twig of *Abiesgeorgei***b, c** mature ascomata. Scale bars: 1 mm (**a**); 300 μm (**b, c**).

**Figure 23.**
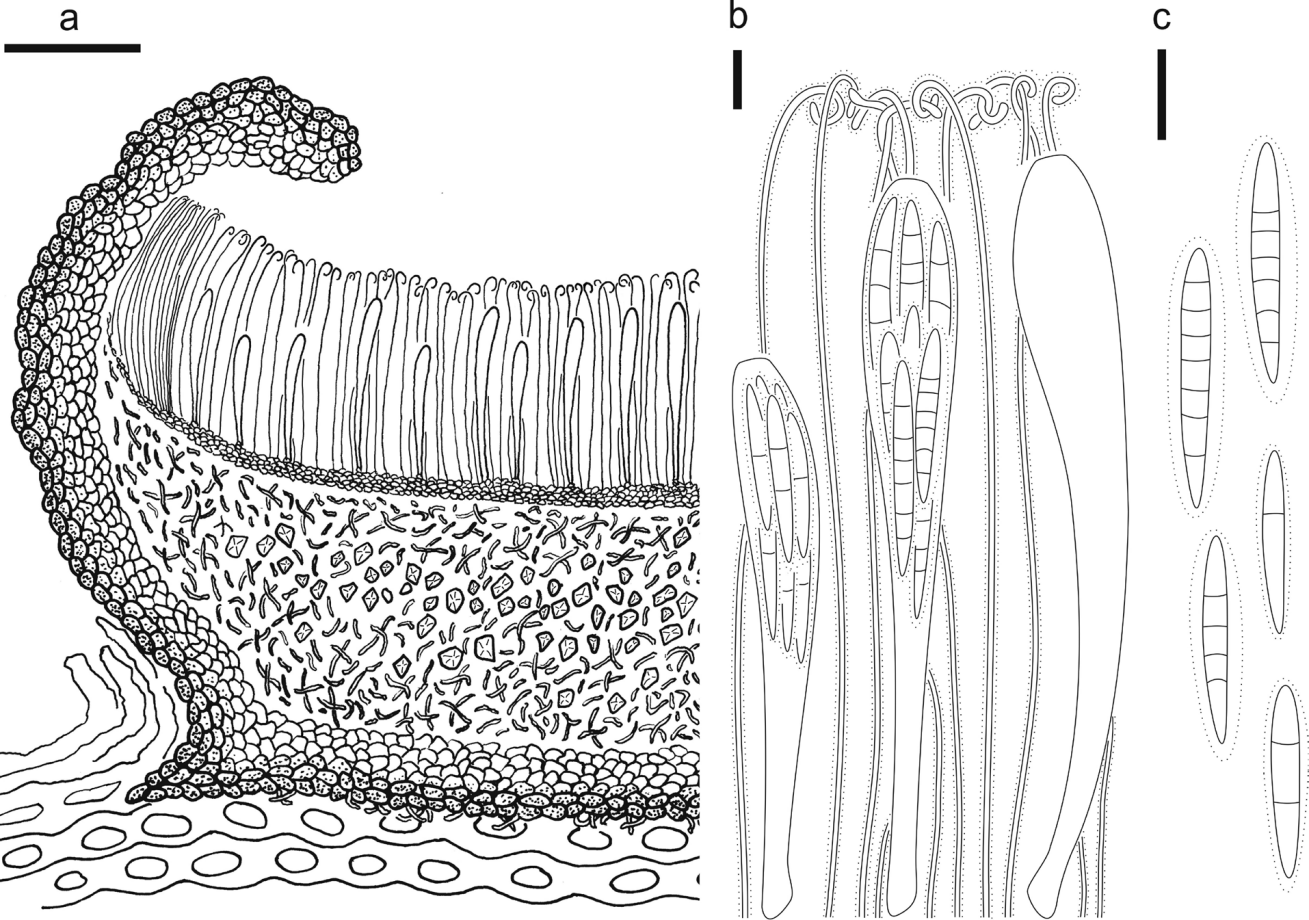
*Neotherryacircinata* on *Abiesgeorgei* (HOU 883/BJTC 2011041, holotype) **a** part of an ascoma in vertical section **b** paraphyses with curled and coiled tips, mature asci with ascospores, and immature ascus **c** liberated ascospores. Scale bars: 100 μm (**a**); 10 μm (**b, c**).

#### ﻿ Neotherrya nematoidea

Taxon classificationAnimaliaRhytismatalesRhytismataceae

Lan Zhuo & C.L. Hou sp. nov.

A0D7EBD4-0A9B-5A23-B17B-942908D0F786

856623

Figs 24 , 25

##### Etymology.

*Nematoidea* (Latin) = nematode-like, refers to the shape of the released ascospores.

##### Diagnosis.

This new species is distinguished from *Neotherryaabieticola* by *N.nematoidea* having filiform ascospores with 1–3-septa.

##### Type.

CHINA, Yunnan Province, Lijiang, Laojunshan, 26.6323°N, 99.7252°E, alt. ca. 3880 m, on twigs of *Abiesgeorgei* (*Pinaceae*), 16 Jul. 2020, C.L. Hou, M.J. Guo, and Q.T. Wang, HOU 1598B (BJTC 2020038, holotype).

##### Sexual morph.

***Ascomata*** on twigs, erumpent from bark, scattered, not associated with pale areas. In surface view, ascomata round to irregular, 750–1000 µm, black (#000000), opening by irregular splits to expose a pale yellow (#ffff9d) hymenium. ***Lips*** absent. In median vertical section, ***covering stroma*** 40–60 μm thick near the center of ascomata, extending to the basal Covering stroma, consisting of carbonized, angular to globose cells. ***Periphysoids*** present. On inner surface of thecovering layer close to opening, a layer formed by periphysoids embedded in gelatinous matrix. ***Excipulum*** well-developed, formed by marginal paraphyses. ***Basal Covering stroma*** absent. ***Internal matrix of Covering stroma*** well-developed, consisting of hyaline hyphae and angular to globose cells. ***Subhymenium*** 15–25 µm thick, consisting of hyaline, angular cells. ***Paraphyses*** aseptate, filiform, not branched, swollen to 2–3 µm diam. at tips, 160–190 × 1–2 µm, covered by a thin gelatinous sheath. ***Asci*** ripening sequentially, clavate, apex obtuse, 110–170 × 10–13 µm, thin-walled, J–, 4-spored. ***Ascospores*** 1–3-septate, usually 3-septate, with shapes of nematode, tapering at both ends, 65–100 × 2–3 μm, hyaline, without gelatinous sheaths.

##### Asexual morph.

***Conidiomata*** and ***zone lines*** not seen.

##### Additional specimens examined.

CHINA, Yunnan Province, Lijiang, Laojunshan, 26.6323°N, 99.7252°E, alt. ca. 3880 m, on twigs of *Abiesgeorgei* (*Pinaceae*), 26 Jul. 2024, C.L. Hou, L. Zhuo, and X.N. Sui, HOU 2305 (BJTC 2024155).

##### Distribution.

Known only from Yunnan Province, China.

##### Notes.

*Neotherryanematoidea* is closely related to *N.abieticola* in the phylogenetic tree, but *N.abieticola* has fusiform ascospores that are much shorter (30–50 × 2.5–4.5 µm) than the ascospores of *N.nematoidea* (65–100 × 2–3 μm) and lacks periphysoids.

**Figure 24.**
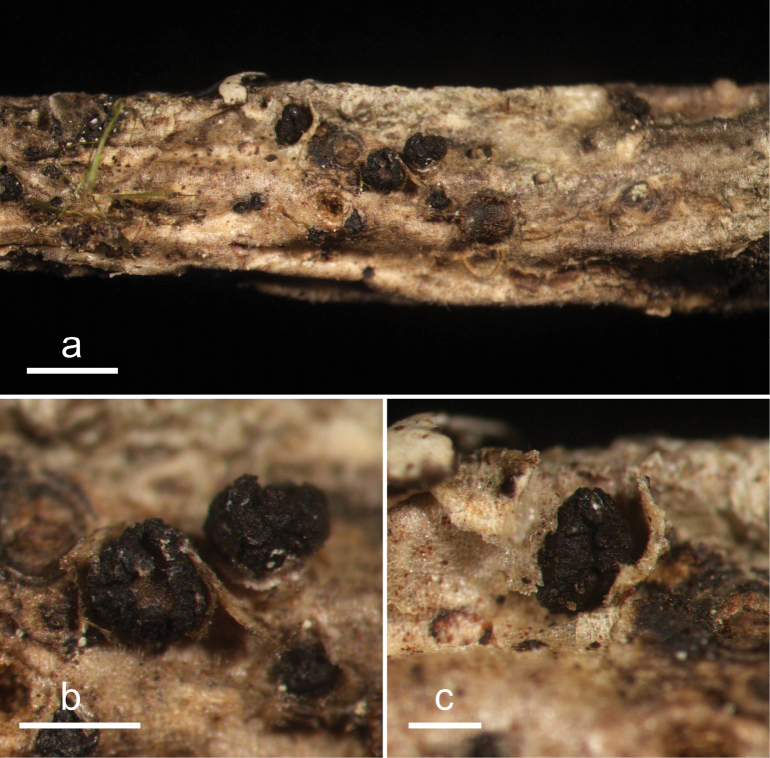
*Neotherryanematoidea* (HOU 1598B/BJTC 2020038, holotype) **a** ascomata on a twig of *Abiesgeorgei***b, c** mature ascomata. Scale bars: 2 mm (**a**); 1 mm (**b**); 500 μm (**c**).

**Figure 25.**
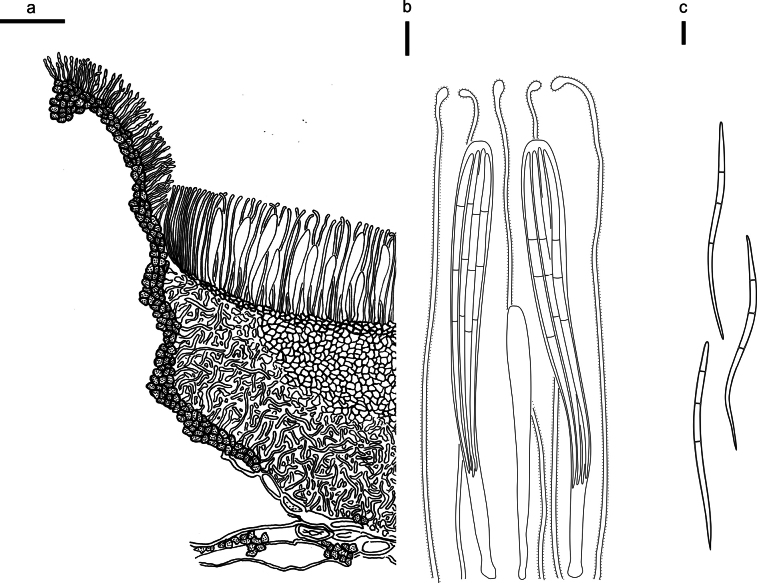
*Neotherryanematoidea* on *Abiesgeorgei* (HOU 1598B /BJTC 2020038, holotype) **a** part of an ascoma in vertical section, periphysoids present, embedded in gelatinous matrix **b** paraphyses, mature asci with ascospores, and immature ascus **c** liberated ascospores. Scale bars: 100 μm (**a**); 10 μm (**b, c**).

#### ﻿ Neotherrya pinicola

Taxon classificationAnimaliaRhytismatalesRhytismataceae

Lan Zhuo & C.L. Hou sp. nov.

D67CD071-F599-539F-9CBB-EEBDE0FFD870

856624

Figs 26 , 27

##### Etymology.

Referring to the host genus *Pinus*.

##### Diagnosis.

This new species is distinguished from other *Neotherrya* species by its aseptate, cylindrical ascospores.

##### Type.

CHINA, Yunnan Province, Lijiang, Shangri-la, Bilahai, alt. ca. 3540 m, on twigs of *Pinusdensata* Mast. (*Pinaceae*), 27 Jun. 2011, C.L. Hou, HOU 919 (BJTC 2011077, holotype).

##### Sexual morph.

***Ascomata*** on twigs, erumpent from bark, scattered, not associated with pale areas. In surface view, ascomata round to irregular, 480–900 µm, dark gray (#373737) to dark brown (#2b180b), opening by irregular splits to expose a black (#000000) hymenium. ***Lips*** absent. In median vertical section, ***covering stroma*** 40–60 μm thick near the center of ascomata, extending to the basal Covering stroma, consisting of the outer remains of the epidermis with the cuticle, an inner layer of carbonized, thick-walled, angular to globose cells, and an innermost layer of hyaline, angular cells. ***Excipulum*** well-developed, formed by marginal paraphyses. ***Basal Covering stroma*** 8–10 μm thick, consisting of dark brown hyphae and carbonized, thick-walled angular cells. ***Internal matrix of Covering stroma*** well-developed, consisting of hyaline, short hyphae. ***Subhymenium*** 10–15 µm thick, consisting of textura intricata. ***Paraphyses*** aseptate, filiform, not branched, swollen to 3–7 µm at their tips, agglutinated to form a dark epithecium, 120–165 × 2–2.5 µm, covered by a thin gelatinous sheath. ***Asci*** ripening sequentially, clavate, apex obtuse-rounded, 100–150 × 8–11 µm, thin-walled, J–, 8-spored. ***Ascospores*** aseptate, cylindrical to fusiform, tapering at both ends, 65–100 × 2–3 μm, hyaline, pluriguttulate, without gelatinous sheaths.

##### Asexual morph.

***Conidiomata*** and ***zone lines*** not seen.

##### Distribution.

Known only from Yunnan Province, China.

##### Notes.

In the phylogenetic tree, *Neotherryapinicola* is closely related to four species, *N.abieticola*, *N.catilliformis*, *N.circinata*, and *N.nematoidea*. These five species form a clade with high support values (MLB = 100%, MPB = 100%, PP = 1.00). Morphologically, *N.pinicola* has sessile ascomata with a well-developed excipulum like the other four species. Based on the phylogenetic analysis and morphological characteristics, *N.pinicola* should be placed in the genus *Neotherrya*. *Neotherryapinicola* differs from the other four species by the cylindrical to fusiform, aseptate ascospores. Therefore, *N.pinicola* is proposed as a distinct species.

**Figure 26.**
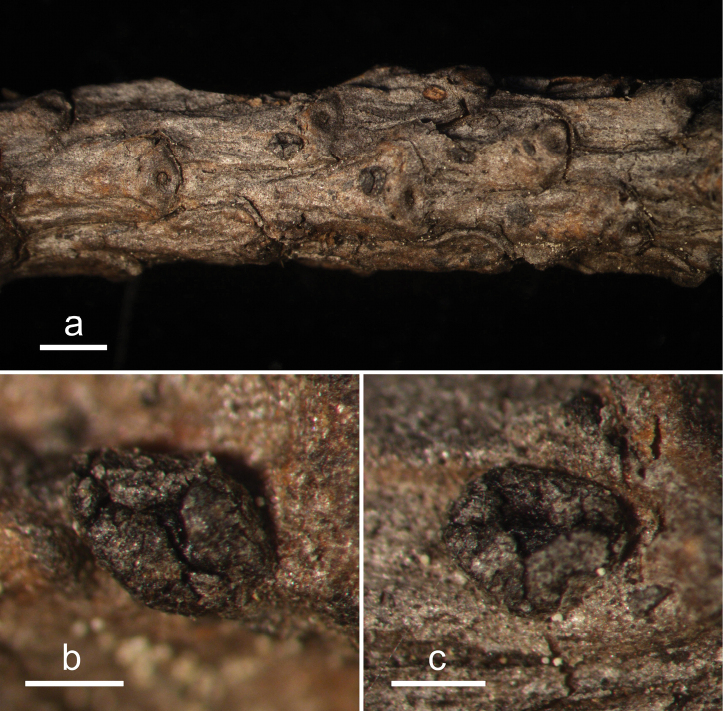
*Neotherryapinicola* (HOU 919/BJTC 2011077, holotype) **a** ascomata on a twig of *Pinusdensata***b, c** mature ascomata. Scale bars: 2 mm (**a**); 500 μm (**b, c**).

**Figure 27.**
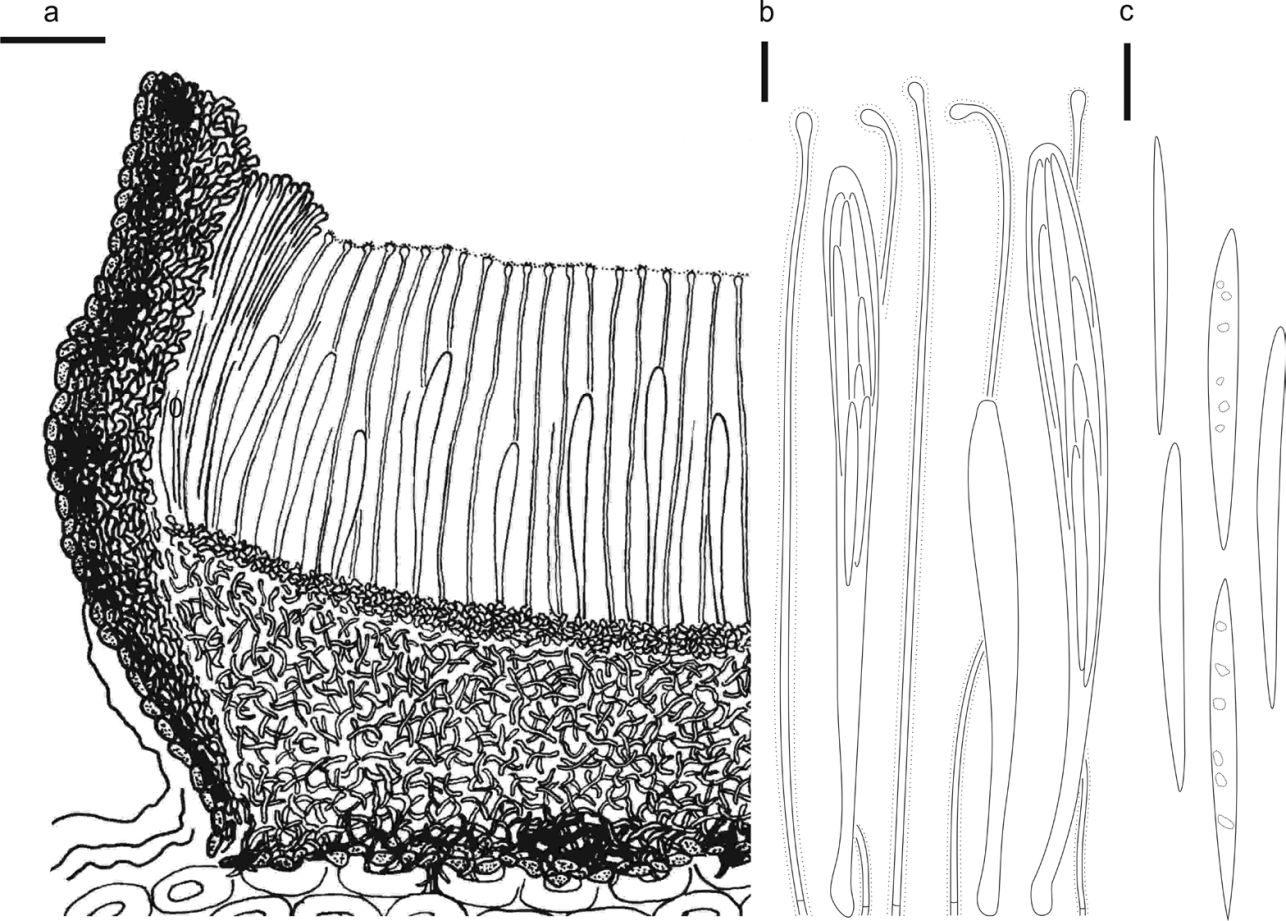
*Neotherryapinicola* on *Pinusdensata* (HOU 919/BJTC 2011077, holotype) **a** part of an ascoma in vertical section **b** paraphyses, mature asci with ascospores, and immature ascus **c** liberated ascospores. Scale bars: 100 μm (**a**); 10 μm (**b, c**).

#### ﻿ Pseudococcomyces

Taxon classificationAnimaliaRhytismatalesRhytismataceae

Lan Zhuo & C.L. Hou gen. nov.

C441823B-C253-5614-A08A-5705A3BE41C6

856625

##### Etymology.

Referring to morphologically similar species of *Coccomyces*.

##### Diagnosis.

This new genus is similar to *Coccomyces*, but lacks a basal Covering stroma.

##### Type.

*Pseudococcomycesyunnanensis* Lan Zhuo & C.L. Hou, described below.

##### Sexual morph.

***Ascomata*** on twigs of *Larix* sp., erumpent from bark, scattered, round or slightly irregular, surface wrinkled, black (#000000), opening by radial or irregular splits. In median vertical section, ***covering stroma*** well developed. ***Basal Covering stroma*** absent. ***Internal matrix of Covering stroma*** present, consisting of hyaline, thin-walled, interwoven hyphae. ***Subhymenium*** consisting of thin-walled, angular cells. ***Paraphyses*** filiform, coiled, interwoven at their tips. ***Asci*** clavate, thin-walled, J–, 8-spored. ***Ascospores*** aseptate, filiform, hyaline, covered by a thick gelatinous sheath.

##### Asexual morph.

***Conidiomata*** and ***zone lines*** not seen.

##### Notes.

Based on the phylogenetic analysis, sequences of *Pseudococcomyces* form a well-supported clade (MLB = 100%, MPB = 99%, PP = 1.00; Clade 8, Fig. 1), and appear to be closely related to *Stipamyces*, but ascomata of *Stipamyces* are stalked. The ascomata of the type species of the new genus is morphologically similar to ascomata of species of the genus *Coccomyces*, but the sequences of species in the new genus on the phylogenetic tree are distant from the sequence of the type species of the genus *Coccomyces* (*Co.tumidus*). Based on morphological and phylogenetic differences, we describe *Pseudococcomyces* as a new genus.

#### ﻿ Pseudococcomyces yunnanensis

Taxon classificationAnimaliaRhytismatalesRhytismataceae

Lan Zhuo & C.L. Hou sp. nov.

91AC3E56-0D9D-5D33-8BAF-A134B0B1EF37

856626

Figs 28 , 29

##### Etymology.

Referring to the name of the province (Yunnan) where the specimen was collected.

##### Diagnosis.

This new species is similar to *Coccomycesirretitus* Sherwood, but *Pseudococcomycesyunnanensis* has paraphyses that are coiled, interwoven at their tips and lacks interwoven hyaline periphysoids.

##### Type.

CHINA, Yunnan Province, Lijiang, Laojunshan, 26.6434°N, 99.7676°E, alt. ca. 3930 m, on twigs of *Larixspeciosa* W. C. Cheng & Y. W. Law (*Pinaceae*), 16 Jul. 2020, C.L. Hou, M.J. Guo, and H. Zhou, HOU 1574 (BJTC 2020012, holotype).

##### Sexual morph.

***Ascomata*** on twigs, erumpent, scattered, not associated with pale areas. In surface view, ascomata round to irregularly elongate, 750–1000 µm diam., black (#000000), erumpent from bark, opening by radial splits to expose a yellow (#ffd400) hymenium. ***Lips*** absent. In median vertical section, ***covering stroma*** 50–70 μm thick near the center of the ascomata, not extending to the base, consisting of an outer layer of remains of the host cortex, an inner layer of carbonized, angular to globose cells, and an innermost layer of pigmented angular to globose cells. ***Basal Covering stroma*** absent. ***Internal matrix of Covering stroma*** 250–300 µm thick, consisting of hyaline, thin-walled, angular cells and interwoven short hyphae. ***Subhymenium*** 30–50 µm thick, consisting of thin-walled, angular cells. ***Paraphyses*** aseptate, filiform, strongly circinate, not swollen at tips, 145–160 × ca. 1 µm. ***Asci*** ripening sequentially, clavate, apex bluntly pointed, 105–155 × 12–18 µm, stalked, thin-walled, J–, 8-spored. ***Ascospores*** aseptate, filiform-clavate, tapering towards base, 40–55 × 1–2 μm, hyaline, covered by a 3–5 μm thick gelatinous sheath.

##### Asexual morph.

***Conidiomata*** and ***zone lines*** not seen.

##### Additional specimens examined.

CHINA, Yunnan Province, Lijiang, Laojunshan, 26.6434°N, 99.7676°E, alt. ca. 3930 m, on twigs of *Larixspeciosa* (*Pinaceae*), 26 Jul. 2024, C.L. Hou, L. Zhuo, and X.N. Sui, HOU 2306 (BJTC 2024156).

##### Distribution.

Known only from Yunnan Province, China.

##### Notes.

In the phylogenetic tree, the sequences of *P.yunnanensis* form a distinct clade. Morphologically, *P.yunnanensis* is closely related to *Co.irretitus*; because both species grow on twigs of *Larix* spp. However, *Co.irretitus* has branched, netlike interwoven hyaline periphysoids immersed in a gel at the inner side of the covering stroma, and the tips of its paraphyses are weakly circinate (Sherwood 1980). Therefore, *P.yunnanensis* is considered to be a new species.

**Figure 28.**
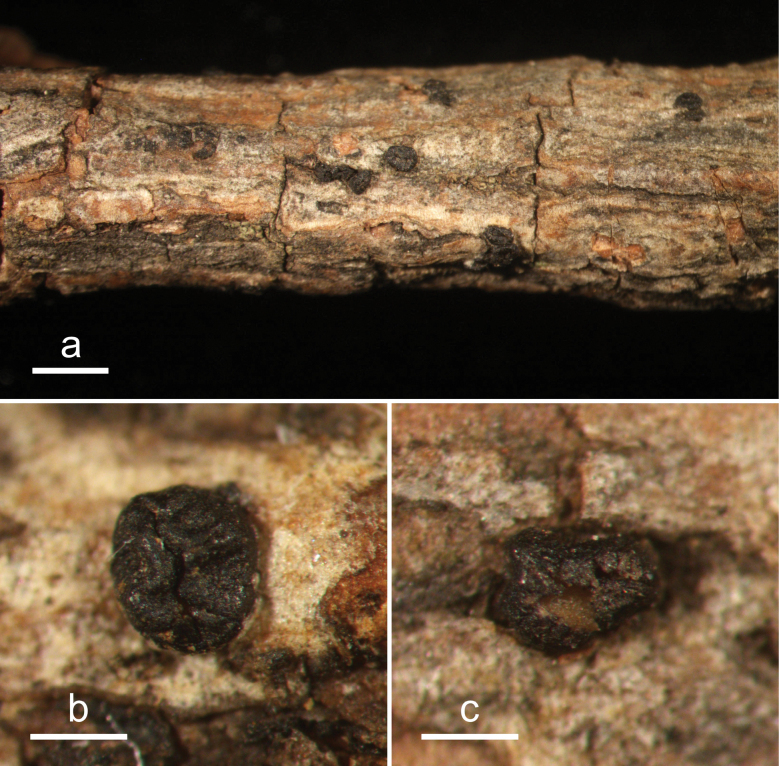
*Pseudococcomycesyunnanensis* (HOU 1574/BJTC 2020012, holotype) **a** ascomata on a twig of *Larixspeciosa***b, c** mature ascomata. Scale bars: 2 mm (**a**); 500 μm (**b, c**).

**Figure 29.**
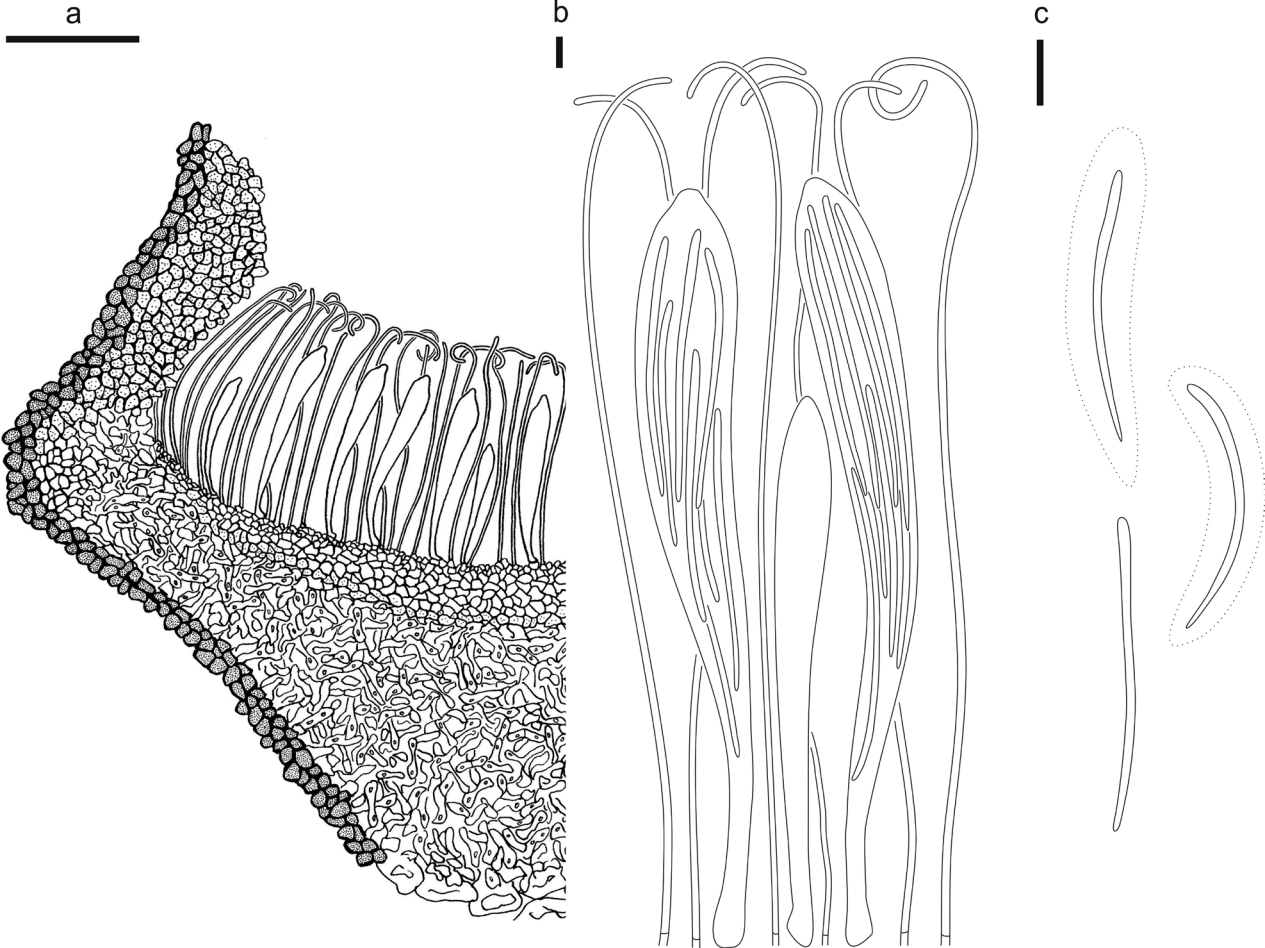
*Pseudococcomycesyunnanensis* on *Larixspeciosa* (HOU 1574/BJTC 2020012, holotype) **a** part of an ascoma in vertical section **b** paraphyses, mature asci with ascospores, and immature ascus **c** liberated ascospores. Scale bars: 100 μm (**a**); 10 μm (**b, c**).

#### ﻿ Stipamyces

Taxon classificationAnimaliaRhytismatalesRhytismataceae

Lan Zhuo & C.L. Hou gen. nov.

CBBFF223-97C3-573E-AE0A-B79B44FCFFE9

856627

##### Etymology.

*Stipa*- from stipes (Latin) = stalk, referring to the stalked ascomata.

##### Diagnosis.

This new genus is similar to *Tryblidiopsis*, but differs in having aseptate ascospores.

##### Type.

*Stipamycespinicola* (R.H. Lei & C.L. Hou) Lan Zhuo & C.L. Hou

##### Sexual morph.

***Ascomata*** on twigs of *Pinus* spp., erumpent, stalked, scattered or aggregate, round or slightly irregular, black, opening by irregular splits. In median vertical section, ***covering stroma*** well developed. ***Basal Covering stroma*** absent. ***Internal matrix of Covering stroma*** present, consisting of hyaline, thin-walled, interwoven hyphae. ***Subhymenium*** consisting of small, hyaline cells. ***Paraphyses*** filiform, circinate at tips. ***Asci*** clavate, thin-walled, J–, 8-spored. ***Ascospores*** aseptate, long-fusiform, hyaline, not covered by a thick gelatinous sheath.

##### Asexual morph.

***Conidiomata*** and ***zone lines*** not seen.

##### Notes.

Based on the phylogenetic analysis, the molecular sequences of *Stipamyces* spp. form a well-supported clade (MLB = 100%, MPB = 82%, PP = 1.00; Clade 7, Fig. 1), and appear to be closely related to *Pseudococcomycesyunnanensis*. *Pseudococcomycesyunnanensis* differs from *Stipamyces* spp. by sessile ascomata. Species within the genus *Tryblidiopsis* also possess stalked ascomata, but the ascospores of *Tryblidiopsis* spp. are typically septate. Furthermore, in the phylogenetic tree, the sequences of species of these two genera are distant. Thus, *Stipamyces* is treated as a new genus.

#### ﻿ Stipamyces massonianae

Taxon classificationAnimaliaRhytismatalesRhytismataceae

Lan Zhuo & C.L. Hou sp. nov.

A2117230-5D86-5870-BB81-58C0C1E6ED37

856628

Figs 30 , 31

##### Etymology.

Referring to the host, *Pinusmassoniana* Lamb. (*Pinaceae*).

##### Diagnosis.

This new species is similar to *Stipamycespinicola*, but the ascomata of *Stipamycesmassonianae* have longer stalks, and paraphyses are sometimes branched at their tips.

##### Type.

CHINA, Anhui Province, Anqing, Yuexi County, alt. ca. 1000 m, on twigs of *Pinusmassoniana*, 20 Apr. 2015, C.L. Hou, HOU 1215 (BJTC 2015001, holotype).

##### Sexual morph.

***Ascomata*** on twigs, aggregated in groups of two to five, not associated with pale areas. In surface view, ascomata round to irregular, 875–1925 µm in diam., 1000–1600 µm high, black (#000000), erumpent from bark, opening by irregular splits to expose a yellow (#ffd400) hymenium. ***Lips*** absent. In median vertical section, ***covering stroma*** 30–50 μm thick near the center of the ascomata, extending to the base, consisting of an outer layer of remains of the host cortex, an inner layer of carbonized, angular cells, and an innermost layer of hyaline angular cells. ***Periphysoids*** on the inner surface of thecovering layer close to the opening, another layer formed by paraphysoids embedded in a gelatinous matrix. ***Basal Covering stroma*** absent. ***Internal matrix of Covering stroma*** well-developed, consisting of hyaline, interwoven hyphae. ***Subhymenium*** 65–80 µm thick, consisting of small, hyaline cells. ***Paraphyses*** aseptate, filiform, circinate, sometimes branched at tips, 100–145 × ca. 1 µm. ***Asci*** ripening sequentially, clavate, slightly truncate at the apex, 40–130 × 4–14 µm, stalked, thin-walled, J–, 8-spored. ***Ascospores*** aseptate, long-fusiform, acute at both ends, 25–40 × 2–4 μm, hyaline, not covered by a gelatinous sheath.

##### Asexual morph.

***Conidiomata*** and ***zone lines*** not seen.

##### Distribution.

Known only from Anhui Province, China.

##### Notes.

The phylogenetic analysis shows that *S.massonianae* is congeneric with *S.pinicola*, but the latter has shorter stalks on ascomata, a thinnercovering layer, and unbranched tips of paraphyses. The sequence similarity of ITS rDNA between these species is 93%, indicating that *S.massonianae* is a distinct species.

**Figure 30.**
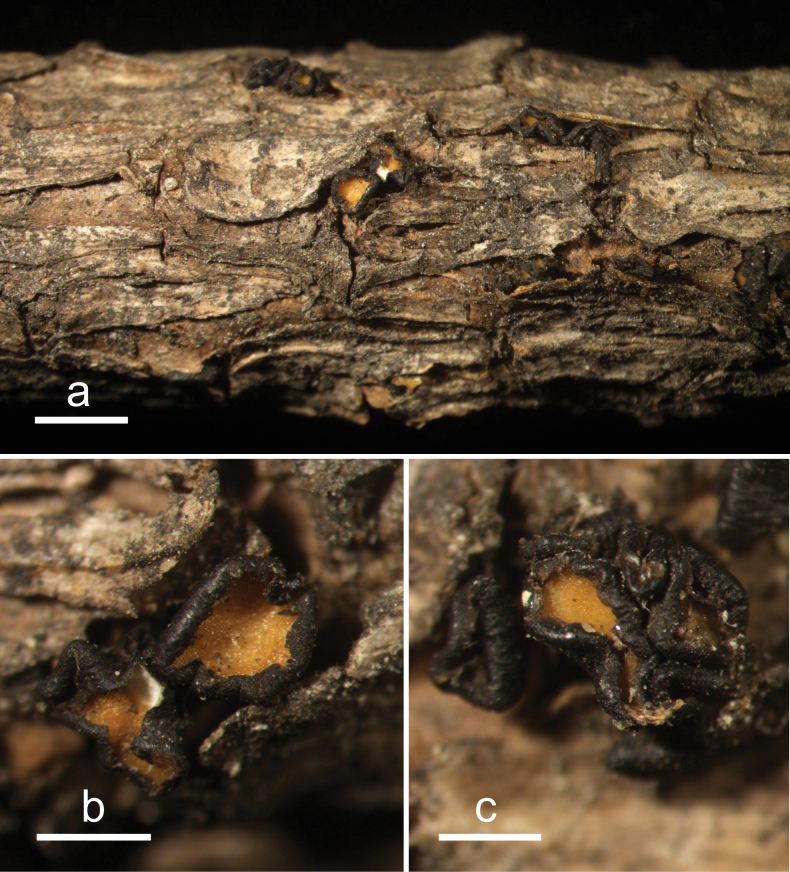
*Stipamycesmassonianae* (HOU 1215/BJTC 2015001, holotype) **a** ascomata on a twig of *Pinusmassoniana***b, c** mature ascomata. Scale bars: 3 mm (**a**); 1 mm (**b, c**).

**Figure 31.**
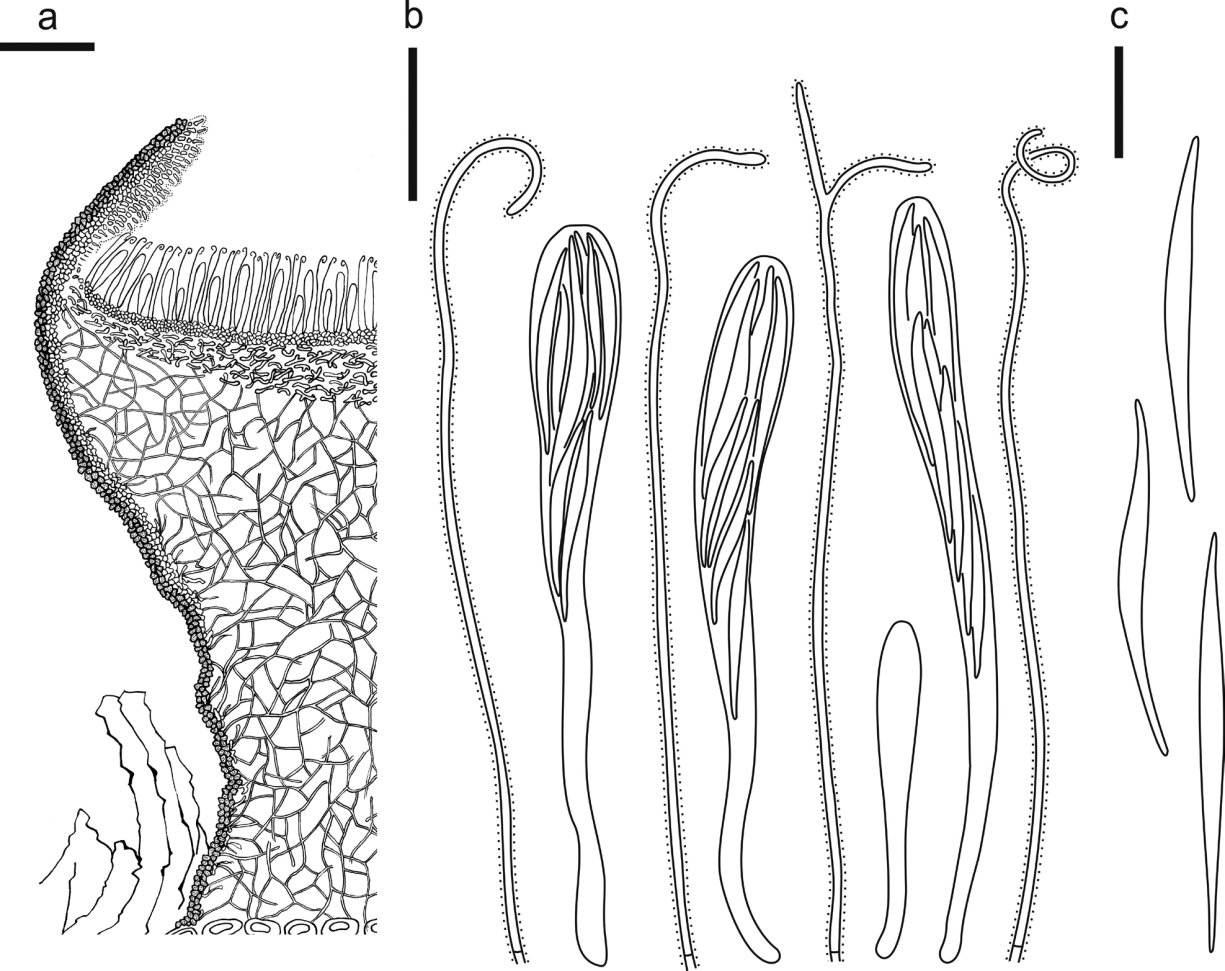
*Stipamycesmassonianae* on *Pinusmassoniana* (HOU 1215/BJTC 2015001, holotype) **a** part of an ascoma in vertical section, periphysoids present, embedded in gelatinous matrix **b** paraphyses, mature asci with ascospores, and immature ascus **c** liberated ascospores. Scale bars: 200 μm (**a**); 25 μm (**b**); 10 μm (**c**).

#### ﻿ Stipamyces pinicola

Taxon classificationAnimaliaRhytismatalesRhytismataceae

(R.H. Lei & C.L. Hou) Lan Zhuo & C.L. Hou comb. nov.

E461397E-8333-5D42-B561-210A1E6B4578

856629

Coccomyces pinicola R.H. Lei & C.L. Hou, Mycotaxon 123: 125. 2013. Basionym.

##### Type.

CHINA, Yunnan Province, Lijiang, Tiejiashan, alt. ca. 2000 m, on twigs of *Pinusarmandii* (*Pinaceae*), 11 Jul. 2007, C.L. Hou, HOU 486A (BJTC 201202, holotype); HOU 486B (BJTC 201212, isotype).

##### Specimens examined.

CHINA, Anhui Province, Yuexi, Miaodaoshan, on twigs of *Pinustaiwanensis* Hayata (*Pinaceae*), 11 Jul. 2007, C.L. Hou, HOU 538 (BJTC 201252); CHINA, Yunnan Province, Lijiang, Laojunshan, 26.6663°N, 99.9388°E, alt. ca. 2345 m, on twigs of *Pinusyunnanensis* Franch. (*Pinaceae*), 16 Jul. 2020, C.L. Hou, HOU 1618 (BJTC 2020060).

##### Notes.

Lei et al. (2013) placed *Co.pinicola* within the genus *Coccomyces* based on morphological characteristics with species of *Coccomyces*. *Coccomycespinicola* clustered weakly with *Colpomaquercinum* in their study. After incorporating sequences from additional specimens, our phylogenetic analysis indicates that these sequences of *Co.pinicola* form a well-supported clade (MLB = 100%, MPB = 82%, PP = 1.00) together with sequences of *S.massonianae*, and are only distantly related to *Col.quercinum*. *Coccomycespinicola* and *S.massonianae* present similar characteristics of ascomata and ascospores, thus *Coccomycespinicola* is transferred to *Stipamyces* here. For a detailed description of this species see Lei et al. (2013).

#### ﻿ Therrya

Taxon classificationAnimaliaRhytismatalesRhytismataceae

Sacc., Michelia 2 (no. 8): 604. 1882.

AB40666A-86C8-516F-82E6-0923FC7783FA

##### Type.

*Therryapini* (Alb. & Schwein.) Höhn., Ber. dt. bot. Ges. 35: 422. 1917.

##### Sexual morph.

***Ascomata*** scattered to clustered, circular to slightly irregular, sessile, dark brown to black, opening by irregular splits. ***Covering stroma*** formed by dark brown (#2b180b) to black (#000000), thick-walled angular cells. ***Epithecium*** present or absent. ***Basal Covering stroma*** poorly or well developed, consisting of carbonized, thick-walled, angular cells. ***Internal matrix of Covering stroma*** well developed, formed by thin-walled, irregular cells or hyaline hyphae, filled or not filled with crystals. ***Subhymenium*** consisting of textura intricata or hyaline cells. ***Paraphyses*** filiform, not branched, tips swollen or not swollen. ***Asci*** ripening sequentially, clavate, thin-walled, J–, 8-spored or 4-spored. ***Ascospores*** aseptate to septate, filiform or fusiform, hyaline, covered or not covered by a gelatinous sheath.

##### Asexual morph.

***Conidiomata*** scattered or gregarious, lenticular, 300–500 μm in diam, dark brown to black, ostiole absent, opening by irregular tears in thecovering layer. In vertical section, conidiomata subepidermal, ***upper layer and basal layer*** 20–60 μm thick, consisting of pale to dark brown textura angularis, with thin- to thick-walled cells. ***Conidiogenous cells*** smooth, cylindrical to ampuliform, discrete, holoblastic, sympodial to synchronous, 9–11.5(–13) × 2–2.5 μm. ***Conidia*** aseptate, hyaline, smooth, cylindrical, straight to curved, with rounded apices, 8–12(–22) × 1–1.5(–2) μm (Description based on Reid and Cain 1960; McMullin et al. 2019).

##### Notes.

Reid and Cain (1960) reviewed the taxonomic history of the genus *Therrya*, listing numerous synonyms under three species names: *Coccomycesstrobi*, *Therryafuckelii* (Rehm) Kujala, and *Therryapini*. Korf (1973) accepted this treatment and emphasize fusiform, multiseptate ascospores lacking gelatinous sheaths as key diagnostic features. Sherwood (1980) further delineated the genus by highlighting its substrate specificity—epiphytic on coniferous substrates—and the epithecium of inflated paraphyses cemented in a brown gel. According to Index Fungorum (https://www.indexfungorum.org) records, there are currently seven legitimate names for the genus *Therrya*, which are *Th.abieticola* C.L. Hou & M. Piepenbr., *Th.eucalypti* Z.Q. Yuan, *Th.fuckelii*, *Th.piceae* A. Funk, *Th.pini*, *Th.pseudotsugae*, and *Th.tsugae* A. Funk. Based on Hou and Piepenbring (2007), *Th.eucalypti* Z.Q. Yuan might be closely related to *Colpoma* Wallr. instead of *Therrya* based on its morphology and host. *Therryaabieticola*, originally placed within this genus, has been transferred to the genus *Neotherrya* in the present study. Molecular data are available for only two species, *Th.fuckelii* and *Th.pini*. Phylogenetic analysis reveals a distinct clade comprising *Co.guizhouensis*, *Co.strobi*, *Parvacoccumpini*, *Th.fuckelii*, and *Th.pini* (Clade 4, Fig. 1), exhibiting morphological similarities with other members of the genus. Based on phylogenetic inference and morphological affinities, it is proposed to transfer *Co.strobi*, *Co.guizhouensis*, and *Pa.pini* into the genus *Therrya*.

#### ﻿ Therrya guizhouensis

Taxon classificationAnimaliaRhytismatalesRhytismataceae

(Y.R. Lin & B.F. Hu) Lan Zhuo & C.L. Hou comb. nov.

8FF0775B-F4C0-5C62-B102-4C57D03580C2

856630

Coccomyces guizhouensis Y.R. Lin & B.F. Hu, in Lin, Liu, Tang & Hu, Acta Mycol. Sin. 13(1): 8. 1994. Basionym.

##### Type.

CHINA, Guizhou Province, Zhijin, alt. ca. 1700 m, on twigs of *Pinusarmandii* Franch. (*Pinaceae*), 2 May 1979, B.F. Hu 16393 (ACAFP 66501, holotype).

##### Notes.

Lin et al. (1994) described *Coccomycesguizhouensis* on twigs of *Pinusarmandii* in the genus *Coccomyces*. In our phylogenetic tree, sequences of *C.guizhouensis* form a sister clade to *Therryapinicola* (synonym *Parvacoccumpini*) and *Therryastrobi* (synonym *Coccomycesstrobi*). *Therryapinicola* differs from *C.guizhouensis* by having a dark zone below the subhymenium and ascospores with more obtuse ends. *Coccomycesstrobi* differs from *C.guizhouensis* by having asci with slightly pointed apices and ascospores that are filiform-clavate with rounded apex. Although the ascospores of *Co.guizhouensis* are not septate, they are fusiform in shape and the apex of paraphyses are sometimes enlarged and form an epithecium. Based on the phylogenetic position and morphological characters, *C.guizhouensis* is transferred to the genus *Therrya* here.

#### ﻿ Therrya pinicola

Taxon classificationAnimaliaRhytismatalesRhytismataceae

Lan Zhuo & C.L. Hou nom. nov.

10F55899-7F7F-5FFC-90A0-C71CAE2A0078

856631

Figs 32 , 33

##### Replacing.

*Parvacoccumpini* R.S. Hunt & A. Funk, Mycotaxon 33: 52. 1988. To avoid a homonym with *Therryapini* (Alb. & Schwein.) Höhn.

##### Type.

CANADA, B.C., Mesachie Lake, on branches of *Pinusmonticola* Douglas ex D.Don (*Pinaceae*) killed by blister rust, 20 Jun. 1986, R.S. Hunt, DAVFP 23420 (holotype).

##### Notes.

Hunt and Funk (1988) described *Parvacoccumpini* on branches of *Pinusmonticola* in the genus *Parvacoccum* R.S. Hunt & A. Funk. In the phylogenetic tree, the molecular sequences of *Th.strobi* form a sister clade to *Parvacoccumpini*. *Parvacoccumpini* differs from *Th.strobi* by having a dark zone below the subhymenium and asci with obtuse apices. Hunt and Funk (1988) established the genus *Parvacoccum* based on the symmetrically fusiform shape of the ascospores with funnel-shaped appendages, separating it from the genus *Coccomyces*. However, the fusiform ascospores and the apically expanded paraphyses suggest that the species be placed in *Therrya*. Given that a species named *Therryapini* (Alb. & Schwein.) Höhn. already exists within the genus *Therrya*, the *Therryapini* (R.S. Hunt & A. Funk) L. Zhuo & C.L. Hou would be a later homonym of the former. In accordance with the International Code of Nomenclature for algae, fungi, and plants, we avoid a nomenclatural conflict by selecting “*pinicola*” as the new epithet for the species previously known as *Parvacoccumpini*.

*Therryapinicola* differs from *Therryapini* by distinct molecular sequence data and morphologically by short, aseptate ascospores.

This is the first record of *Therryapinicola* for China and on the new host *Pinuspumila* (Pall.) Regel.

##### Specimens examined.

CHINA, Heilongjiang Province, Yichun, Xing’anling Arboretum, 47.7474°N, 128.8899°E, alt. ca. 300 m, on twigs of *Pinuspumila* (*Pinaceae*), 16 Jun. 2024, C.L. Hou, L. Zhuo, and Y. Gao, HOU 2237 (BJTC 2024097); 47.7474°N, 128.8899°E, alt. ca. 300 m, on twigs of *Pi.pumila*, 16 Jun. 2024, C.L. Hou, L. Zhuo, and Y. Gao, HOU 2238B (BJTC 2024098).

**Figure 32.**
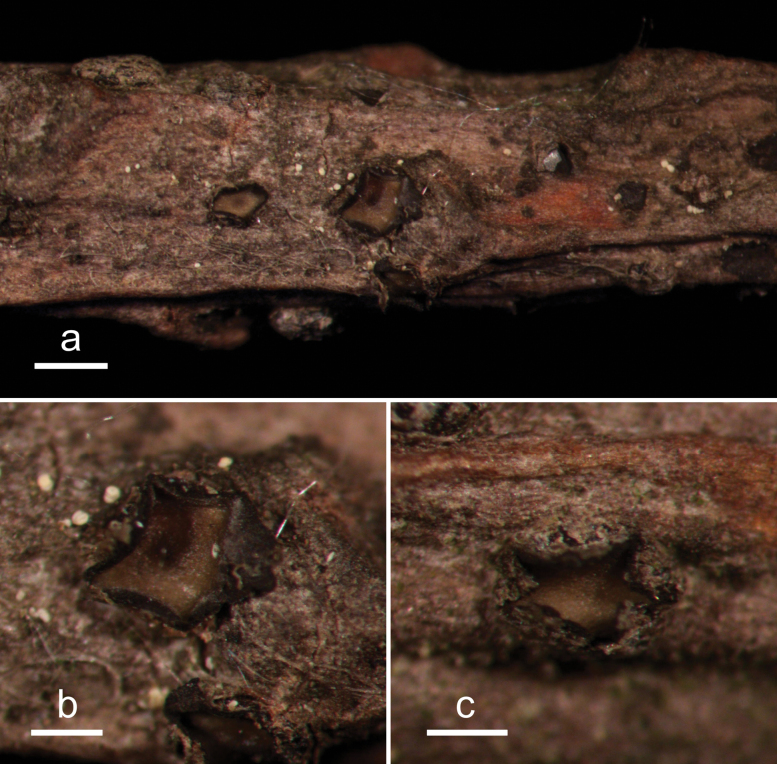
*Therryapinicola* (HOU 2237/BJTC 2024097) **a** ascomata on a twig of *Pinuspumila***b, c** mature ascomata. Scale bars: 1 mm (**a**); 500 μm (**b**); 1 mm (**c**).

**Figure 33.**
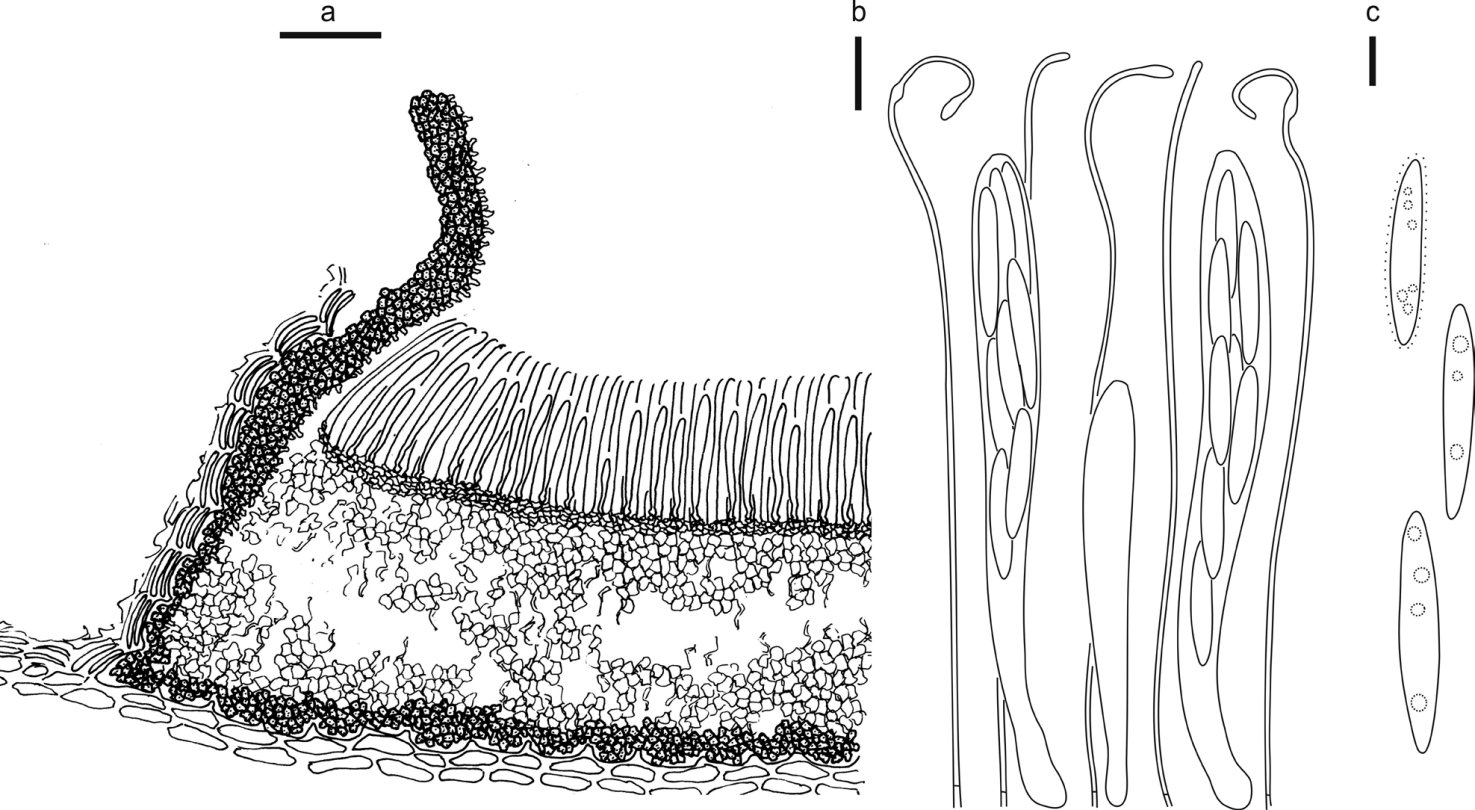
*Therryapinicola* on *Pinuspumila* (HOU 2237 /BJTC 2024097) **a** part of an ascoma in vertical section **b** paraphyses, mature asci with ascospores, and immature ascus **c** liberated ascospores. Scale bars: 100 μm (**a**); 10 μm (**b**); 5 μm (**c**).

#### ﻿ Therrya strobi

Taxon classificationAnimaliaRhytismatalesRhytismataceae

(J. Reid & Cain) Lan Zhuo & C.L. Hou comb. nov.

2694DC96-8995-5983-B7F8-3F3F7D65D80E

856632

Coccomyces strobi J. Reid & Cain, Can. J. Bot. 39(5): 1127. 1961. Basionym.

##### Type.

CANADA, Ontario, Simcoe County, Midhurst, on *Pinusstrobus* L. (*Pinaceae*), 4 Jun. 1951, R.F. Cain, TRTC 23,578 (holotype).

##### Notes.

Reid and Cain (1961) described *Co.strobi* on branches of *Pinusstrobus* in the genus *Coccomyces*. In the phylogenetic tree, *Co.strobi* is closely related to *Therrya* species; ITS rDNA sequences similarities with other *Therrya* species range from 91.86%–97.62%. In the phylogenetic analyses, sequences from seven isolates of *Co.strobi* formed two clades. Among them, isolates NB-645C, NB-641A, DAOMC251937, DAOMC251589, and DAOMC251575 were submitted by Tanney J.B. in McMullin et al. (2018). Their study provides detailed information on isolation sources, and morphological illustrations, which agree with the typical description of *Co.strobi*, and establishes the correspondence between morphology and phylogeny. Therefore, the sequences from these isolates form a clade representing *Co.strobi*. In contrast, the sequence from isolate AFTOL-ID 1250 formed a separate branch, suggesting that it may represent a different species. Further examination of the material is needed to confirm its taxonomic status. The tips of the paraphyses of *Co.strobi* are curled, coiled, tending to form an epithecium, therefore, according to the morphological characters and phylogenetic relationship, *Co.strobi* belongs to the genus *Therrya*.

#### ﻿ Tryblidiopsis

Taxon classificationAnimaliaRhytismatalesRhytismataceae

P. Karst., Bidr. Känn. Finl. Nat. Folk 19: 262. 1871.

AFAA7D84-489B-58E9-A728-1548A4B7EA77

##### Type.

*Tryblidiopsispinastri* (Pers.) P. Karst., Bidr. Känn. Finl. Nat. Folk 19: 262. 1871.

##### Sexual morph.

***Ascomata*** scattered to clustered, circular, stalked, black, opening by irregular splits. ***Covering stroma*** formed by dark brown (#2b180b) to black (#000000), thick-walled angular cells. ***Basal Covering stroma*** usually absent. ***Internal matrix of Covering stroma*** well developed, formed by hyaline, branched, septate hyphae embedded in gelatinous matrix, filled or not filled with crystals. ***Subhymenium*** consisting of textura intricata. ***Paraphyses*** filiform, branched or not branched, with slightly swollen tips. ***Asci*** ripening sequentially, cylindrical to clavate, thin-walled, J–, 8-spored or 4-spored. ***Ascospores*** 0–6 septate, filiform, fusiform to clavate, hyaline, covered by a gelatinous sheath (Description based on Livsey and Minter 1994).

##### Notes.

Livsey and Minter (1994) reviewed the generic concept, and considered *Tryblidiopsis* to be a monotypic genus. *Tryblidiopsismagnesii* Tanney & Seifert, *Try.sichuanensis* S. Wang, P.F. Cannon & C.L. Hou, and *Try.sinensis* S. Wang, P.F. Cannon & C.L. Hou have been described subsequently (Wang et al. 2014b; Tanney and Seifert 2019). These species all have stalked ascomata, grow on *Picea* spp., and have ascospores that are fusiform to clavate, septate, with a thick gelatinous sheath. In the present study, three new species are added to this genus based on morphological analyses, host substratum, and phylogenetic data (Clade 2, Fig. 1).

#### ﻿ Tryblidiopsis changbaishanensis

Taxon classificationAnimaliaRhytismatalesRhytismataceae

Lan Zhuo & C.L. Hou sp. nov.

06309E06-2207-5E2F-830E-436B35FE2D41

856633

Figs 34 , 35

##### Etymology.

Referring to the name of the location (Changbaishan) where the type specimen was collected.

##### Diagnosis.

This new species is similar to *Tryblidiopsissinensis*, but differs by having a brown covering stroma, curved ascospores, and tips of paraphyses strongly swollen to 3–5 µm.

##### Type.

CHINA, Jilin Province, Yanbian Chaoxianzu Autonomous Prefecture, Antu County, Changbaishan, 42.0867°N, 128.0745°E, alt. ca. 1600 m, on twigs of Piceajezoensisvar.komarovii (V. N. Vassil.) W. C. Cheng & L. K. Fu (*Pinaceae*), 14 Jun. 2024, C.L. Hou, L. Zhuo, and Y. Gao, HOU 2210 (BJTC 2024070, holotype).

##### Sexual morph.

***Ascomata*** on twigs, erumpent from bark, scattered, not associated with pale areas. In surface view, ascomata round to elliptical, 1000–1450 × 800–1250 µm, as seen from the side 600–800 µm high, brown (#48240a) to dark brown (#2b180b), opening by irregular splits to expose a yellow (#ffe562) hymenium. ***Lips*** absent. In median vertical section, ***covering stroma*** 40–60 μm thick near center of ascomata, consisting of an outer layer of carbonized, angular to globose cells and an inner layer of hyaline, angular to globose cells. ***Basal Covering stroma*** 5–7 µm thick, located between the subhymenium and the internal matrix of the Covering stroma, consisting of brown to dark brown globose cells. ***Internal matrix of Covering stroma*** well-developed, consisting of hyaline, thin-walled, gelatinous cells and hyaline hyphae, mixed with irregular crystals. ***Subhymenium*** 15–20 µm thick, consisting of textura intricata and hyaline, angular to globose cells. ***Paraphyses*** aseptate, filiform, not branched, swollen to 3–5 µm at tips, 120–140 × 2 µm. ***Asci*** ripening sequentially, clavate, apex obtuse, 110–120 × 15–22 µm, thin-walled, J–, 8-spored. ***Ascospores*** aseptate, curved fusiform, 16–25 × 4–5 μm, hyaline, covered by a 3–4 µm thick gelatinous sheath.

##### Asexual morph.

***Conidiomata*** and ***zone lines*** not seen.

##### Additional specimens examined.

CHINA, Jilin Province, Yanbian Chaoxianzu Autonomous Prefecture, Antu County, Changbaishan, 42.0867°N, 128.0745°E, alt. ca. 1600 m, on twigs of Piceajezoensisvar.komarovii (*Pinaceae*), 14 Jun. 2024, C.L. Hou, L. Zhuo, and Y. Gao, HOU 2211 (BJTC 2024071).

##### Distribution.

Known only from Jilin Province, China.

##### Notes.

Wang et al. (2017) cited the immature specimen of *Tryblidiopsis* sp. (HOU 662) on *Piceajezoensis* (Siebold & Zucc.) Carrière collected from Jilin Province, Changbaishan. In the context of the present study, we returned to this original collection site and successfully collected the mature specimens HOU 2210 and HOU 2211. The nrITS similarity between the newly collected specimens and HOU 662 is 98%. Furthermore, phylogenetic analysis shows that the newly collected specimens and specimen HOU 662 cluster together. These results indicate that the newly collected specimens HOU 2210 and HOU 2211 are the same species as specimen HOU 662.

The sequences of *Try.changbaishanensis* form a sister clade with the sequences of *Try.pinastri* and *Try.sinensis*. *Tryblidiopsischangbaishanensis* differs from *Try.pinastri* by having aseptate ascospores and tips of paraphyses strongly swollen to 3–5 µm. *Tryblidiopsischangbaishanensis* differs from *Try.sinensis* by having curved ascospores and a melanized basal Covering stroma between the subhymenium and the internal matrix of Covering stroma.

**Figure 34.**
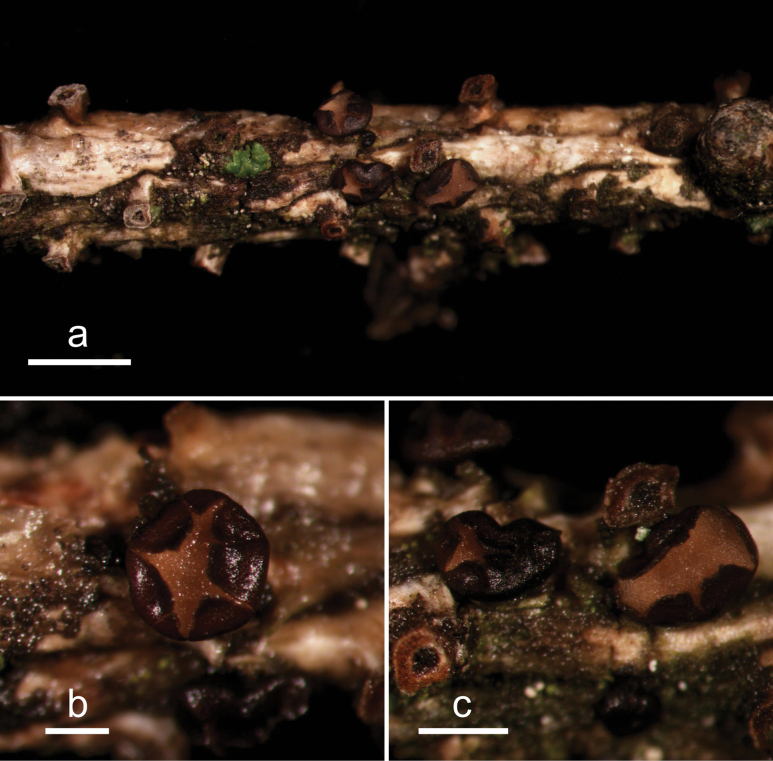
*Tryblidiopsischangbaishanensis* (HOU 2210/BJTC 2024070, holotype) **a** ascomata on a twig of Piceajezoensisvar.komarovii**b, c** mature ascomata. Scale bars: 2 mm (**a**); 500 μm (**b**); 1 mm (**c**).

**Figure 35.**
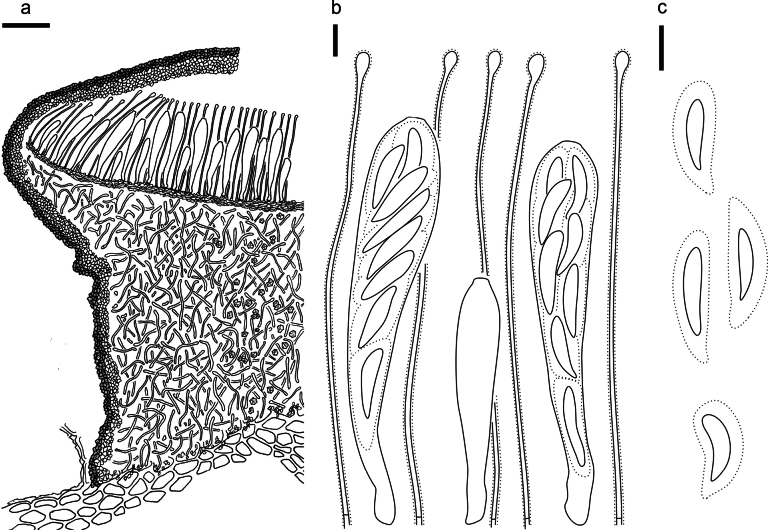
*Tryblidiopsischangbaishanensis* on Piceajezoensisvar.komarovii (HOU 2210 /BJTC 2024070, holotype) **a** part of an ascoma in vertical section **b** paraphyses swollen at tips, mature asci with ascospores, and immature ascus **c** liberated ascospores. Scale bars: 100 μm (**a**); 10 μm (**b, c**).

#### ﻿ Tryblidiopsis melanostroma

Taxon classificationAnimaliaRhytismatalesRhytismataceae

Lan Zhuo & C.L. Hou sp. nov.

DC2E29E4-1860-5F61-8672-262FEE47E652

856634

Figs 36 , 37

##### Etymology.

Melano- (Greek), referring to the melanized basal Covering stroma between the subhymenium and the internal matrix of the Covering stroma.

##### Diagnosis.

This new species is similar to *Tryblidiopsissichuanensis*, but differs by a melanized basal Covering stroma between the subhymenium and an internal matrix of the Covering stroma, and an internal matrix filled with crystals.

##### Type.

CHINA, Yunnan Province, Lijiang, Laojunshan, 26.6452°N, 99.7838°E, alt. ca. 3290 m, on twigs of *Picealikiangensis* (Franch.) E. Pritz. (*Pinaceae*), 17 Aug. 2023, C.L. Hou, L. Zhuo, and S.Y. Zhao, HOU 2105 (BJTC 2023235, holotype).

##### Sexual morph.

***Ascomata*** on twigs, erumpent from the bark, scattered or aggregated in groups of three to five ascomata, not associated with pale areas. In surface view, ascomata round to elliptical, 1000–1150 × 800–1000 µm, as seen from the side 900–1200 µm high, black (#000000), opening by irregular splits to expose a yellow (#ffe562) hymenium. ***Lips*** absent. In median vertical section, ***covering stroma*** 50–110 μm thick near the center of ascomata, consisting of an outer layer of carbonized, angular to globose cells and an inner layer of hyaline, angular to globose cells. ***Basal Covering stroma*** 15–25 µm thick, located between the subhymenium and the internal matrix of the Covering stroma, consisting of brown to dark brown, globose cells. ***Internal matrix of the Covering stroma*** well-developed, consisting of hyaline, thin-walled, gelatinous cells and hyaline hyphae, filled with irregular crystals. ***Subhymenium*** 10–20 µm thick, consisting of hyaline, angular to globose cells. ***Paraphyses*** aseptate, filiform, not branched, slightly swollen at tips, 135–165 × 1 µm. ***Asci*** ripening sequentially, clavate, apex obtuse, 120–150 × 15–18 µm, thin-walled, J–, 8-spored. ***Ascospores*** 1-septate, fusiform, 20–25 × 3–4 μm, hyaline, covered by a ca. 2 µm thick gelatinous sheath.

##### Asexual morph.

***Conidiomata*** and ***zone lines*** not seen.

##### Additional specimens examined.

CHINA, Yunnan Province, Lijiang, Laojunshan, 26.6452°N, 99.7838°E, alt. ca. 3290 m, on twigs of *Picealikiangensis* (*Pinaceae*), 26 Jul. 2024, C.L. Hou, L. Zhuo, and X.N. Sui, HOU 2307 (BJTC 2024157).

##### Distribution.

Known only from Yunnan Province, China.

##### Notes.

The multi-locus gene analysis indicates that the sequences of *Tryblidiopsismelanostroma* form a well-supported clade sister to the sequences of *Try.sichuanensis*. *Tryblidiopsismelanostroma* is morphologically similar to *Try.sichuanensis*, but the latter has an internal matrix of Covering stroma without crystals and lacks a melanized basal Covering stroma between the subhymenium and the internal matrix of the Covering stroma. Interestingly, a similar melanization phenomenon has been observed in *Try.magnesii*, where the inner wall of the covering stroma is melanized (Magnes 1997; Tanney and Seifert 2019). The taxonomic relevance of this feature remains uncertain and requires further study. Therefore, *Try.melanostroma* is considered to be a distinct species.

**Figure 36.**
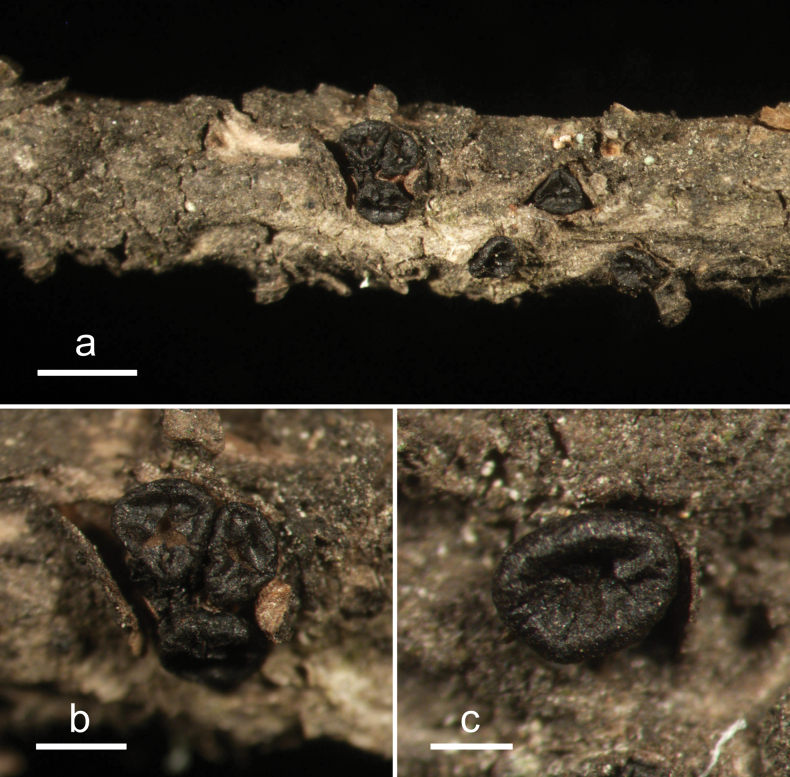
*Tryblidiopsismelanostroma* (HOU 2105/BJTC 2023235, holotype) **a** ascomata on a twig of *Picealikiangensis***b, c** mature ascomata. Scale bars: 2 mm (**a**); 1 mm (**b**); 500 μm (**c**).

**Figure 37.**
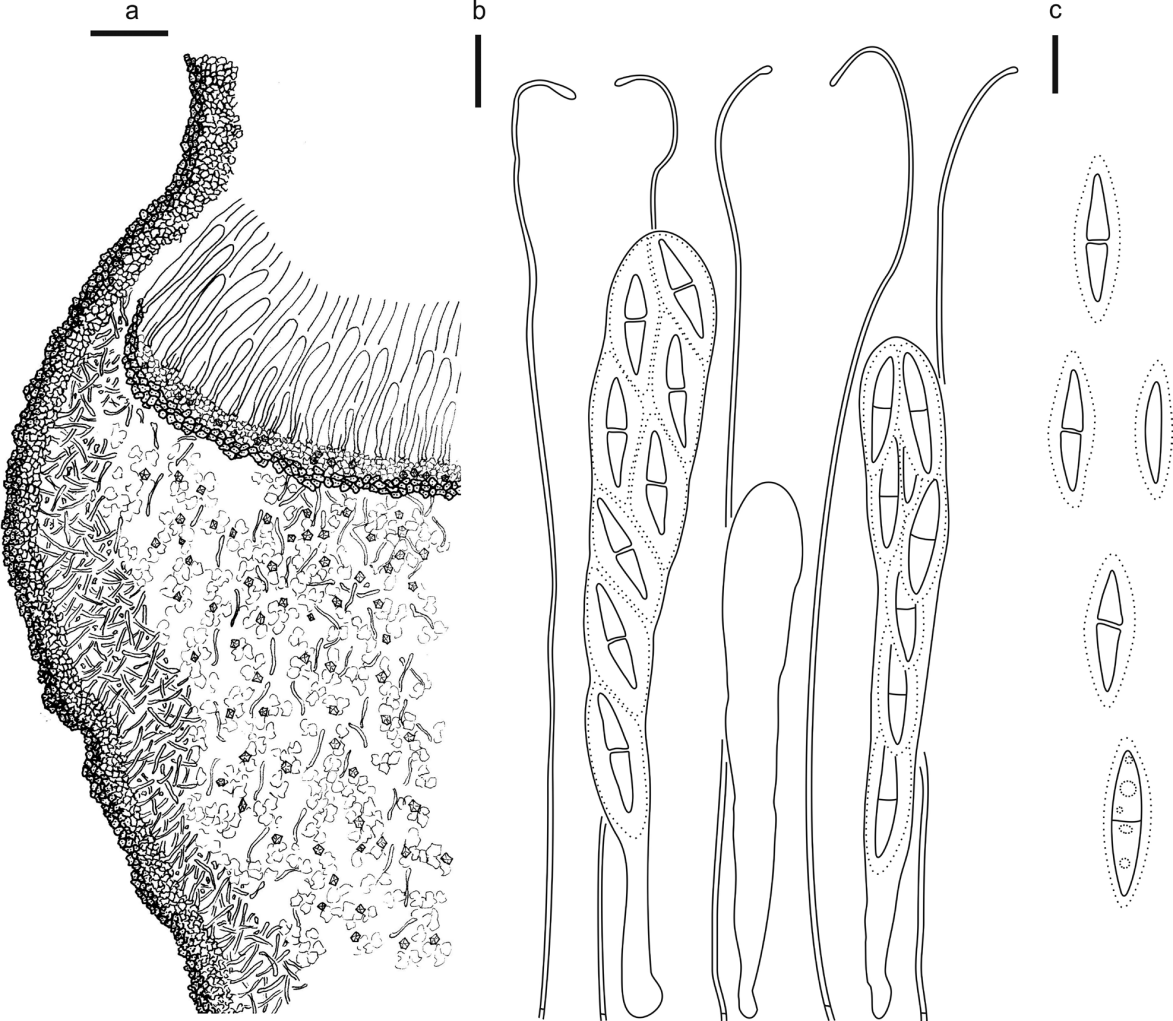
*Tryblidiopsismelanostroma* on *Picealikiangensis* (HOU 2105/BJTC 2023235, holotype) **a** part of an ascoma in vertical section, having a melanized basal Covering stroma between the subhymenium and an internal matrix of the Covering stroma **b** paraphyses, mature asci with ascospores, and immature ascus **c** liberated ascospores. Scale bars: 100 μm (**a**); 15 μm (**b**); 10 μm (**c**).

#### ﻿ Tryblidiopsis multiseptata

Taxon classificationAnimaliaRhytismatalesRhytismataceae

Lan Zhuo & C.L. Hou sp. nov.

389084C1-75F4-5F62-B8E2-96239D11F509

856635

Figs 38 , 39

##### Etymology.

Referring to ascospores with3–6 septae.

##### Diagnosis.

This new species is distinguished from other *Tryblidiopsis* species by its 4-spored asci and 3–6-septate ascospores.

##### Type.

CHINA, Yunnan Province, Lijiang, Laojunshan, 26.6452°N, 99.7838°E, alt. ca. 3290 m, on twigs of *Picealikiangensis* (*Pinaceae*), 17 Aug. 2023, C.L. Hou, L. Zhuo, and S.Y. Zhao, HOU 2107 (BJTC 2023238, holotype).

##### Sexual morph.

***Ascomata*** on twigs, erumpent from the bark, scattered, not associated with pale areas. In surface view, ascomata subround to elliptical, 1500–2200 × 1100–1400 µm, as seen from the side 600–800 µm high, black (#000000), opening by irregular splits to expose a dark gray (#373737) hymenium. ***Lips*** absent. In median vertical section, ***covering stroma*** 35–60 μm thick near the center of ascomata, consisting of an outer layer of carbonized, angular to globose cells and an inner layer of hyaline, angular to globose cells. ***Basal Covering stroma*** absent. ***Internal matrix of the Covering stroma*** well-developed, consisting of hyaline hyphae, filled with irregular crystals. ***Subhymenium*** 25–35 µm thick, consisting of hyaline, angular to globose cells. ***Paraphyses*** aseptate, filiform, not branched, slightly swollen at tips, 160–185 × 1 µm, covered by a ca. 1 µm thick gelatinous sheath. ***Asci*** ripening sequentially, clavate, apex obtuse, 120–150 × 18–25 µm, thin-walled, J–, 4-spored, no aborted ascospores were observed. ***Ascospores*** 3–6-septate, rod-shape, tapering to narrow base, 40–50 × 4–6 μm, hyaline, covered by a 2–3 µm thick gelatinous sheath.

##### Asexual morph.

***Conidiomata*** and ***zone lines*** not seen.

##### Additional specimens examined.

CHINA, Yunnan Province, Lijiang, Laojunshan, 26.6452°N, 99.7838°E, alt. ca. 3290 m, on twigs of *Picealikiangensis* (*Pinaceae*), 17 Aug. 2023, C.L. Hou, L. Zhuo, and S.Y. Zhao, HOU 2104 (BJTC 2023234); 26.6619°N, 99.7758°E, alt. ca. 3280 m, on twigs of *Pi.likiangensis*, 20 Jun. 2021, C.L. Hou, M.J. Guo, and H. Zhou, HOU 1750 (BJTC 2021061).

##### Distribution.

Known only from Yunnan Province, China.

##### Notes.

The phylogenetic analysis shows that sequences of this new species form a separate clade and cluster with sequences of *Try.changbaishanensis*, *Try.magnesii*, *Try.melanostroma*, *Try.pinastri*, *Try.sichuanensis*, and *Try.sinensis*. Morphologically, *Try.multiseptata* can be distinguished from other *Tryblidiopsis* species by its 4-spored asci and 3–6-septate ascospores.

**Figure 38.**
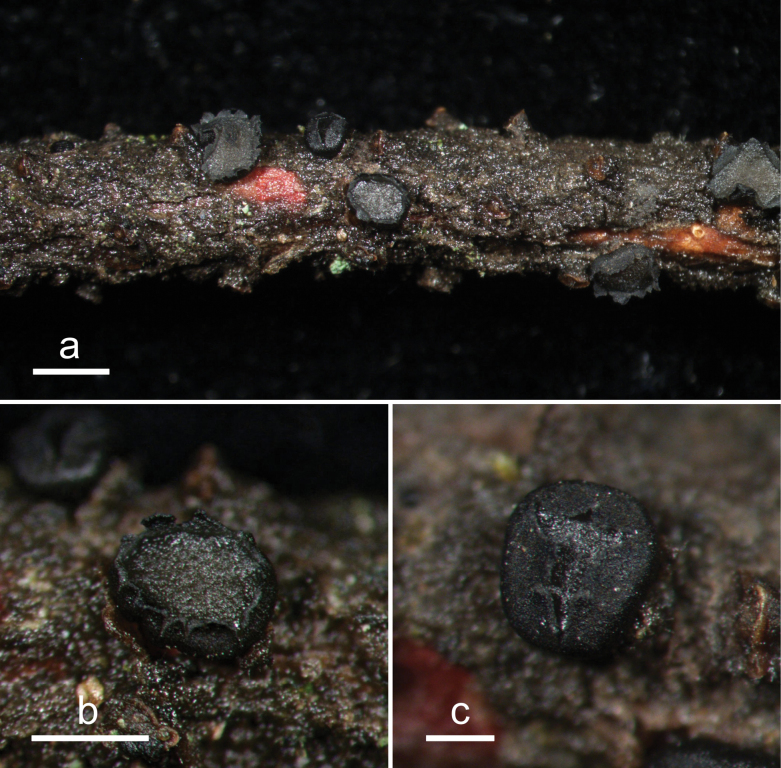
*Tryblidiopsismultiseptata* (HOU 2107/BJTC 2023238, holotype) **a** ascomata on a twig of *Picealikiangensis***b, c** mature ascomata. Scale bars: 2 mm (**a**); 1 mm (**b**); 500 μm (**c**).

**Figure 39.**
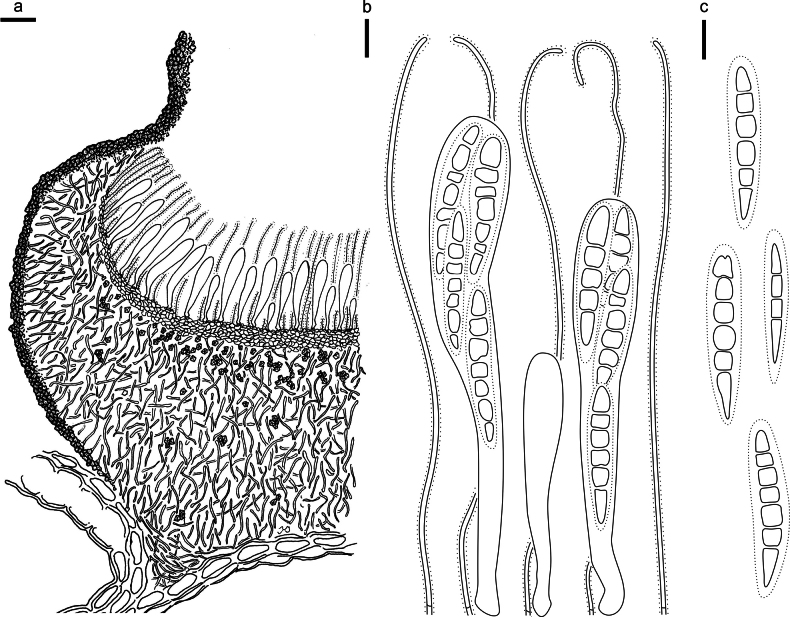
*Tryblidiopsismultiseptata* on *Picealikiangensis* (HOU 2107/BJTC 2023238, holotype) **a** part of an ascoma in vertical section **b** paraphyses, mature asci with ascospores, and immature ascus **c** liberated ascospores. Scale bars: 100 μm (**a**); 10 μm (**b, c**).

#### ﻿ Tryblidiopsis yunnanensis

Taxon classificationAnimaliaRhytismatalesRhytismataceae

Lan Zhuo & C.L. Hou sp. nov.

718D40CB-E242-5DC1-90E8-F774726129B8

856636

Figs 40 , 41

##### Etymology.

Referring to the name of the province (Yunnan) where the specimen was collected.

##### Diagnosis.

This new species is distinguished from other *Tryblidiopsis* species by its filiform ascospores each with 1–3-septa, and occurrence on a non-*Picea* host.

##### Type.

CHINA, Yunnan Province, Lijiang, Laojunshan, alt. ca. 3200 m, on twigs of *Tsugachinensis* (Franch.) Pritz. (*Pinaceae*), 11 Jul. 2007, C.L. Hou, HOU 487 (BJTC 201203, holotype).

##### Sexual morph.

***Ascomata*** on twigs, erumpent from the bark, scattered, not associated with pale areas. In surface view, ascomata round to irregular, 800–1100 µm, as seen from the side 350–550 µm high, black (#000000), opening by irregular splits to expose a pale yellow hymenium. ***Lips*** absent. In median vertical section, ***covering stroma*** 70–100 μm thick near the center of ascomata, consisting of an outer layer of carbonized, angular to globose cells, and an inner layer of hyaline, angular to globose cells. ***Basal Covering stroma*** absent. ***Internal matrix of Covering stroma*** well-developed, consisting of hyaline hyphae and angular to globose cells. ***Subhymenium*** 20–40 µm thick, consisting of textura intricata and hyaline, angular to globose cells. ***Paraphyses*** aseptate, filiform, not branched, curved, sightly swollen at tips, 110–160 × 2–2.5 µm, covered by a thin gelatinous sheath. ***Asci*** ripening sequentially, clavate, apex obtuse, 90–135 × 11–18 µm, thin-walled, J–, 8-spored. ***Ascospores*** 1–3-septate, filiform, tapering at both ends, 45–90 × 2–4 μm, hyaline, covered by a thin gelatinous sheath.

##### Asexual morph.

***Conidiomata*** and ***zone lines*** not seen.

##### Additional specimens examined.

CHINA, Yunnan Province, Lijiang, Xinzhu village, alt. ca. 2400 m, on twigs of *Tsugachinensis* (*Pinaceae*), 20 Jul. 2013, C.L. Hou, HOU 1116B (BJTC 2013047).

##### Distribution.

Known only from Yunnan Province, China.

##### Notes.

Wang et al. (2017) indicated that *Tryblidiopsis* species are host-specific and grow only on *Picea* spp., but the new species *Try.yunnanensis* grows on *Tsugachinensis*. Four species of *Rhytismataceae* are known from *Tsuga* spp, namely *Coccomycesheterophyllae* A. Funk, *Coccomycesmertensianae* Sherwood, *Discocainiatreleasei* (Sacc.) J. Reid & A. Funk, and *Therryatsugae*. However, for none of them molecular sequence data are available. Morphologically, *Co.heterophyllae*, *Co.mertensianae*, and *D.treleasei* differ from *Try.yunnanensis* by aseptate ascospores, and *Th.tsugae* differs from *Try.yunnanensis* by ascomata opening by a single longitudinal split. Usually, *Tryblidiopsis* spp. have fusiform to clavate ascospores and more or less stalked ascomata, whereas *Try.yunnanensis* has filiform ascospores and sessile ascomata. Sequence similarities of ITS rDNA between *Try.yunnanensis* and other sequences of *Tryblidiopsis* species are 86.63%–92.61%, and nrLSU rDNA are 91.16%–97.72%. Based on the differences of ascomata, ascospores, host and sequence similarities, it seems that a new genus should be erected to accommodate this species. To avoid establishing too many genera, however, we tentatively assign this species to *Tryblidiopsis*.

**Figure 40.**
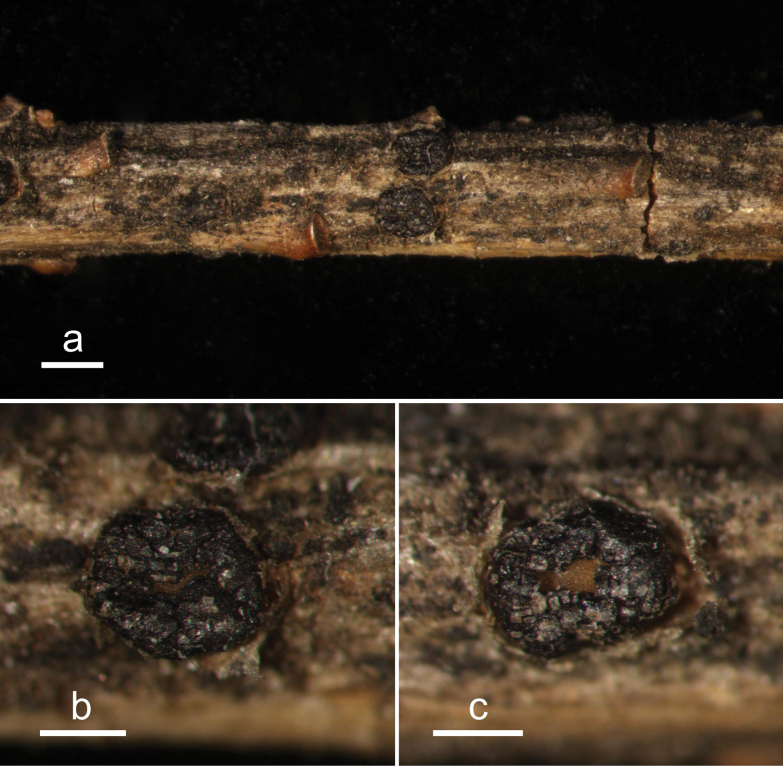
*Tryblidiopsisyunnanensis* (HOU 487/BJTC 201203, holotype) **a** ascomata on a twig of *Tsugachinensis***b, c** mature ascomata. Scale bars: 1 mm (**a**); 500 μm (**b, c**).

**Figure 41.**
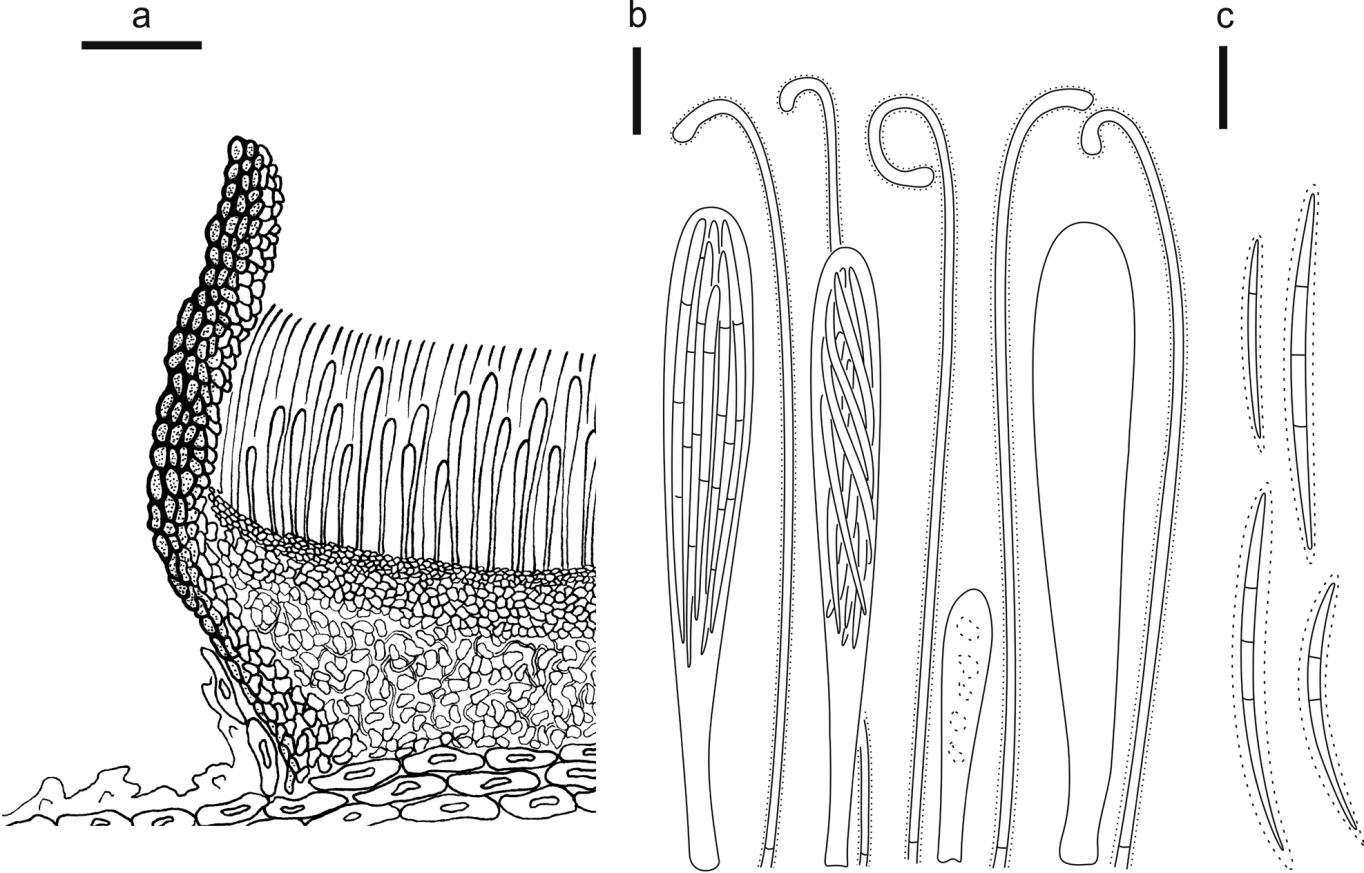
*Tryblidiopsisyunnanensis* on *Tsugachinensis* (HOU 487/BJTC 201203, holotype) **a** part of an ascoma in vertical section **b** paraphyses, mature asci with ascospores, and immature ascus **c** liberated ascospores. Scale bars: 100 μm (**a**); 15 μm (**b**); 20 μm (**c**).

#### ﻿ Tryblidiopsis

Taxon classificationAnimaliaRhytismatalesRhytismataceae

sp. 1

82D073E1-35D2-58EA-A8BF-CA47F6866EE0

Fig. 42

##### Specimen examined.

CHINA, Sichuan Province, Xiaojin County, Jiajinshan, on twigs of *Picea* sp. (*Pinaceae*), 25 Jul. 2006, C.L. Hou, HOU 288 (BJTC 2006015).

##### Notes.

In the multigene phylogenetic analysis, the sequence of the specimen HOU 288 forms a distinct clade closely related to the sequence of *Try.melanostroma* and *Try.sichuanensis*. The ITS rDNA sequence similarity of this specimens and these two species is 97%. Because the specimen is immature, it is labelled as *Tryblidiopsis* sp. 1.

**Figure 42.**
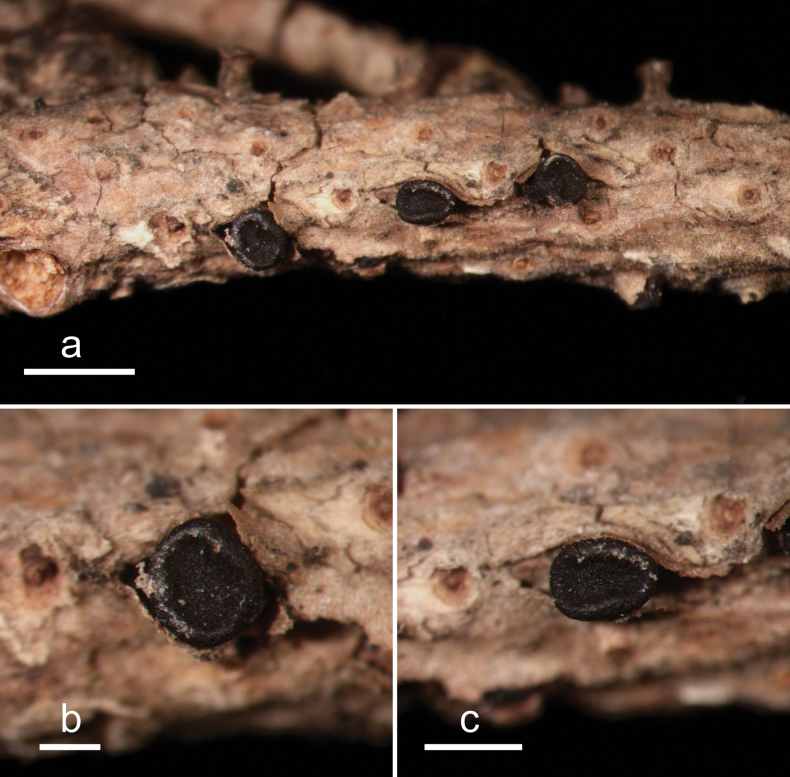
*Tryblidiopsis* sp. 1 (HOU 288/BJTC 2023053) **a** immature ascomata on a twig of *Picea* sp. **b, c** immature ascomata. Scale bars: 2 mm (**a**); 500 μm (**b**); 1 mm (**c**).

#### ﻿ Tryblidiopsis

Taxon classificationAnimaliaRhytismatalesRhytismataceae

sp. 2

01644033-5D16-5F8B-B097-9D40745647EF

Fig. 43

##### Specimen examined.

CHINA, Heilongjiang, Mudanjiang, Jingpohu National Scenic Area, on twigs of *Picea* sp. (*Pinaceae*), 5 Aug. 2011, C.L. Hou, HOU 956 (BJTC 2011114).

##### Notes.

In the multigene phylogenetic analysis, the sequence of this specimen clusters with the sequences of *Try.changbaishanensis*. The ITS rDNA sequence similarity of HOU 956 and *Try.changbaishanensis* is 97%. Unfortunately, no mature ascomata of this specimen have been obtained, so specimen HOU 956 is labelled as *Tryblidiopsis* sp. 2.

**Figure 43.**
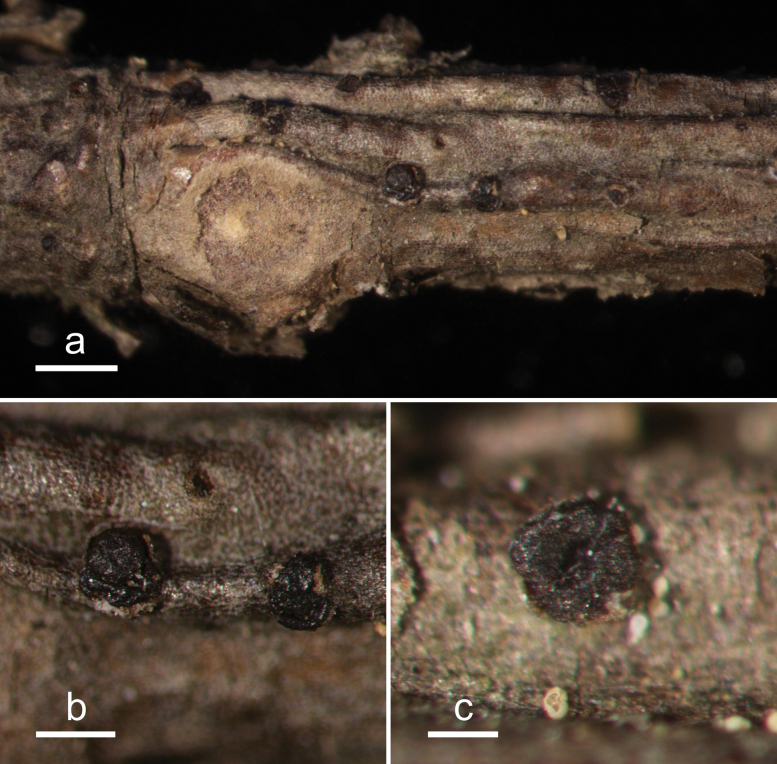
*Tryblidiopsis* sp. 2 (HOU 956/BJTC 2011114) **a** ascomata on a twig of *Picea* sp. **b, c** immature ascomata. Scale bars: 1 mm (**a**); 500 μm (**b**); 200 μm (**c**).

## ﻿Discussion

### ﻿Limitations of ascospore morphology for taxonomy

Morphology of ascospores should not be used as a sole criterion for distinguishing between genera of *Rhytismatales*. The study by Lantz et al. (2011), which utilized ancestral state reconstruction, indicated that filiform spores are of little use to delimit genera. Additionally, the development of ascospore septa is related to external environmental conditions such as humidity, hence relying solely on the shape or septa of ascospores as morphological characters for the classification of genera is not advisable (Darker 1967; Lantz et al. 2011). This is also evident in some taxa in the current study, such as the genus *Tryblidiopsis* and the newly proposed genus *Neotherrya*. These genera form two distinct, highly supported clades in the phylogenetic tree. However, based on morphological characters, species within the genus *Neotherrya* exhibit shapes of ascospores that are filiform, fusiform, or cylindrical. Within the genus *Tryblidiopsis*, different species have different numbers of septa, from aseptate, uniseptate, to multiseptate. The reliance on a single morphological characteristic or phylogenetic analysis as the sole criterion for species can obscure inconsistencies among fungal groups, leading to inaccurately defined species boundaries (Chethana et al. 2021; Wang et al. 2023; Guo et al. 2024). This also explains the speciose and para/polyphyletic genera *Lophodermium* and *Coccomyces*, which share filiform ascospores. The diversity in ascospore morphology within a single genus, as observed in *Neotherrya* and *Tryblidiopsis*, indicates that such characteristics can vary significantly even among closely related species. This variability underscores the complexity of fungal taxonomy and suggests that a more comprehensive approach, which may include genetic analysis and other morphological traits, is necessary for accurate classification. Therefore, this research integrates evidence from various morphological characteristics, phylogenetic analysis based on multiple genetic loci, and specificity to both host and infected organ, to precisely delineate the taxonomic units within *Rhytismatales*. The findings highlight the importance of considering multiple criteria when delineating fungal genera to ensure a robust and biologically meaningful classification system.

### ﻿Rich interspecific variation in *Neotherrya*

Species of *Neotherrya* form a distinct clade with high support in the phylogenetic tree. The species within this genus exhibit considerable morphological variability, concerning ascospore shape, the number of septa in ascospores, and the number of spores in each ascus. These differences make it impossible to classify species in *Neotherrya* based solely on morphological traits. The ITS sequence similarity among species within this genus also varies significantly, ranging from 87.01% to 95.66%. While there is no universally accepted threshold for ITS sequence divergence, Vu et al. (2019) proposed a genus-level threshold of 94.3% for ITS sequences, which further emphasizes the variability in *Neotherrya*ITS sequences. Because the nrITS region can be quite variable, the more conserved nrLSU region is often used to resolve relationships at higher taxonomic levels (Hillis and Dixon 1991; Untereiner et al. 2001). In *Neotherrya*, the nrLSU sequences similarity is relatively high (94.48% to 99.18%), providing strong support for grouping these five species together in the same genus.

Four of the five species of *Neotherrya* were collected on Laojunshan in Yunnan, part of the Hengduan Mountains, a region known for its high diversity of conifer species. The concentration of species highlights the rich fungal diversity in this region, which may be due to unique environmental conditions, niche specialization, or co-evolution with the host conifers.

The significant differences in ITS sequences’ similarity among species suggest there may be undiscovered species in genus *Therrya*. This points to the need to further explore the Hengduan Mountains to uncover hidden biodiversity. Future studies should focus on collecting more specimens and analyzing a wider range of genetic markers to better understand the species boundaries and evolutionary relationships in these taxa.

### ﻿Host specificity

Some taxa within the order *Rhytismatales* exhibit strong host specificity. In the study by Wang et al. (2023), *Rhytismataceae* s. s. was proposed based on phylogeny and host relationships. Species of many genera grow on the twigs of conifers and occur on specific groups of hosts. For example, species within the newly described genus *Cryptococcomyces* are exclusively found on twigs of *Juniperus* spp., while *Stipamyces* are only found on *Pinus* spp. Host preferences are also evident within the genus *Therrya*. Phylogenetic analysis shows that *Th.strobi*, *Th.pinicola*, and *Th.guizhouensis* cluster together in a clade, with species predominantly associated with hosts in PinussubsectionStrobus. In contrast, *Th.fuckelii* and *Th.pini* form a separate clade, more closely associated with hosts in subsectionPinus. This pattern suggests the possibility of further subdivision within these lineages based on host specificity. The genus *Neotherrya* includes five species, four of which are found on *Abies* twigs, with only one species, *N.pinicola*, living on twigs of *Pinusdensata*. Similarly, the seven known species of *Tryblidiopsis* all live on twigs of *Picea* spp. except for *Try.yunnanensis*, which is found on *Tsugachinensis*. Both *N.pinicola* and *Try.yunnanensis* occupy relatively independent positions on the phylogenetic tree. However, due to some morphological similarities and a reluctance to excessively divide the genera, these two species are provisionally classified within *Neotherrya* and *Tryblidiopsis*, respectively. Host organ specificity can also be observed in *Rhytismatales* on conifers, In addition to host specificity, substrate preferences can also be observed in *Rhytismatales* on conifers, i.e., needle-inhabiting species are separated from twig-inhabiting species in the phylogenetic analysis (Fig. 1) (Ortiz-Garcia et al. 2003; Hou 2004; Guo et al. 2024). While some species show host specificity, others have a broad host range and are found living on different host plants. For instance, *Coccomycesirretitus* and *Pseudographiselatina* have both been discovered on *Abies*, *Larix*, *Pinus*, and *Picea*. Species found on bark are likely to be saprotrophs, with broader host preferences. This contrasts with species like *Therrya* spp. and *Tryblidiopsis* spp., which are more likely endophytes, functioning as biotrophs. These species likely exhibit more restricted host preferences during their living phase and transition to saprotrophic behavior upon host tissue death. This distinction may help explain the observed host preferences and ecological niches within *Rhytismatales*.

### ﻿Key to taxa of *Rhytismatales* on conifers worldwide

**Table:** 

1	Cell wall of ascospores reacts opaque dark blue to dark purple in iodine-based reagents	***Pseudographis* (19)**
–	Cell wall of ascospores does not reacts opaque dark blue to dark purple in iodine-based reagents	**2**
2	Ascospores muriform	***Triblidium* (*Tri.sherwoodiae*)**
–	Ascospores not muriform	**3**
3	Ascomata stipitate	**4**
–	Ascomata sessile	**5**
4	Ascospores with gelatinous sheath	***Tryblidiopsis* (20)**
–	Ascospores without gelatinous sheath	***Stipamyces* (27)**
5	Ascomata round or irregular	**6**
–	Ascomata elliptical or elongate	**13**
6	Ascospores elliptical	***Zeus* (*Z.olympius*)**
–	Ascospores not elliptical	**7**
7	Basal Covering stroma absent	***Pseudococcomyces* (*P.yunnanensis*)**
–	Basal Covering stroma present	**8**
8	Ascomata growing on *Juniperus* twigs	***Cryptococcomyces* (28)**
–	Ascomata growing on twigs of other hosts	**10**
9	Paraphyses coiled or bent at tips	***Discocainia* (*D.treleasei*)**
–	Paraphyses not coiled at tips	11
10	Ascal apex usually rounded or acute; ascospores generally aseptate	***Coccomyces* (32)**
–	Ascal apex usually obtuse	**12**
11	Internal matrix of Covering stroma consisting of hyaline hyphae; ascospores cylindrical	***Neotherrya* (44)**
–	Internal matrix of Covering stroma consisting of globoes or angular cells; ascospores fusiform	**12**
12	Paraphyses not swollen at tips	***Abiomyces* (*A.laojunshanensis*)**
–	Paraphyses usually swollen at tips	***Therrya* (49)**
13	Ascomata elliptical or elongate, often curved, sometimes branched, often parasitic on living twigs or branches	***Colpoma* (55)**
–	Ascomata elliptical to broadly elliptical	**14**
14	Lips present	**15**
–	Lips absent	**18**
15	Ascomata develop a darkened lower wall at an early stage in ascomatal development, before the hymenium is differentiated, and before a darkened upper wall has developed	***Bivallium* (61)**
–	Ascomata with simultaneous development of darkened upper and lower walls or darkened upper wall develops first	**16**
16	Ascomata elliptical or cylindrical	***Hypoderma* (62)**
–	Ascospores filiform	**17**
17	Lips creamy white; asci rostrate at apex at maturity	***Labivalidus* (64)**
–	Lips other colors; asci round or subacute at apex	***Lophodermium* (65)**
18	Ascospores 0–1 septate	***Hypohelion* (*Hy.shennongjianum*)**
–	Ascospores 3 septate	***Xyloschizon* (*X.weirianum*)**
19	Ascospores muriform, broadly elliptical	***Pseudographiselatina***
–	Ascospores phragmosporous, elongate	***Pseudographispinicola***
20	Asci 4-spored	***Tryblidiopsismultiseptata***
–	Asci 8-spored	**21**
21	Ascospores 1–3 septate	***Tryblidiopsisyunnanensis***
–	Ascospores 0–1 septate	**22**
22	Melanized layer between the subhymenium and internal matrix of Covering stroma	**23**
–	No melanized layer between the subhymenium and internal matrix of Covering stroma	**24**
23	Tips of paraphyses strongly swollen 3–5 µm	***Tryblidiopsischangbaishanensis***
–	Tips of paraphyses only slightly swollen	***Tryblidiopsismelanostroma***
24	Gelatinous sheaths of ascospores with extended hyaline appendages at apex and base	***Tryblidiopsissinensis***
–	Gelatinous sheaths of ascospores without appendages at apex and base	**25**
25	Melanized inner layer of the covering stroma	***Tryblidiopsismagnesii***
–	No melanized inner layer of the covering stroma	**26**
26	Multilocular conidiomata	***Tryblidiopsissichuanensis***
–	Unilocular conidiomata	***Tryblidiopsispinastri***
27	Ascomata with longer stalks, paraphyses branched at tips	***Stipamycesmassonianae***
–	Ascomata with shorter stalks, paraphyses not branched at tips	***Stipamycespinicola***
28	Paraphyses coiled or bent at tips	**29**
–	Paraphyses not coiled at tips	**31**
29	Basal Covering stroma absent	***Cryptococcomycesniger***
–	Basal Covering stroma present	**30**
30	Internal matrix of Covering stroma consisting of crystals; consistent gelatinous sheaths consistency of ascospores	***Cryptococcomycescarbostomaticus***
–	Internal matrix of Covering stroma without crystals; different gelatinous sheaths consistency of ascospores at apex	***Cryptococcomycesjuniperi***
31	Ascospores each with gelatinous caps	***Cryptococcomycescrystallinus***
–	Ascospores without gelatinous caps	***Cryptococcomycesoccultus***
32	Branched, netlike interwoven hyaline periphysoids immersed in a gel lined on the inside ofcovering layer	***Coccomycesirretitus***
–	Netlike interwoven hyaline periphysoids absent	**33**
33	Ascospores 7–8 septate	***Coccomycesvillaevicosae***
–	Ascospores aseptate	**34**
34	Basal Covering stroma absent or poorly developed	**35**
–	Basal Covering stroma well developed	**39**
35	Coveringstroma barely carbonized on the exterior	***Coccomycesheterophyllae***
–	Coveringstroma strongly carbonized on the exterior	**36**
36	Inner layer of covering stroma composed of interwoven hyphae embedded in a brown gelatinous matrix	***Coccomycesmertensianae***
–	Inner layer of covering stroma not composed of interwoven hyphae	**37**
37	Paraphyses embedded in gel, area in the upper part of the asci with brown globose granules, which turn bright olive green after the addition of KOH	***Coccomycespumilio***
–	Paraphyses not embedded in gel, without globose granules	**38**
38	Paraphyses branched, subhymenium faintly brown	***Coccomycesparvulus***
–	Paraphyses not branched; subhymenium hyaline	***Coccomycespetersii***
39	Paraphyses septate	**40**
–	Paraphyses aseptate	**41**
40	Paraphyses partly recurved at the tips and nodose	***Coccomycespseudotsugae***
–	Paraphyses not recurved at the tips or nodose	***Coccomyceslijiangensis***
41	Internal matrix of Covering stroma thick, up to 500 μm	***Coccomycespapillatus***
–	Internal matrix of Covering stroma thin	**42**
42	Ascospores fusiform	***Coccomycesatactus***
–	Ascospores filiform	**43**
43	Paraphyses branched, enlarged to 3 μm at apex	***Coccomycesbipartitus***
–	Paraphyses not branched, weakly circinate, enlarged to 2–3 μm at apex	***Coccomycescembrae***
44	Ascospores septate	**45**
–	Ascospores aseptate	**46**
45	Ascospores fusiform, 30–50 × 2.5–4.5 μm	***Neotherryaabieticola***
–	Ascospores cylindrical, 65–100 × 2–3 μm	***Neotherryapinicola***
46	Ascospores 1–3-septate, usually 3-septate, nematode-like	***Neotherryanematoidea***
–	Ascospores more than 3-septate, cylindrical	**47**
47	Ascospores covered by a 2–3 µm thick gelatinous sheath; paraphyses curled and coiled at tips	***Neotherryacircinata***
–	Ascospores without gelatinous sheath; paraphyses swollen to 2–3 µm at tips	***Neotherryacatilliformis***
48	Ascospores aseptate	**49**
–	Ascospores septate	**51**
49	Ascospores filiform-clavate	***Therryastrobi***
–	Ascospores narrowly fusiform or fusiform	**50**
50	Ascospores with a funnel shaped appendage at both ends, 16–18 × 2 μm	***Therryapinicola***
–	Ascospores without appendage, 21–45 × 1.6–2.8 μm	***Therryaguizhouensis***
51	Asci 4-spored; ascospores 8–12-celled	***Therryafuckelii***
–	Asci 8-spored; ascospores ≤ 8 cells	**52**
52	Ascospores broadly fusiform to clavate	***Therryapiceae***
–	Ascospores narrowly fusiform or acicula	**53**
53	Asci acutely rounded at the apex	***Therryapseudotsugae***
–	Asci blunt or obtuse at the apex	**54**
54	Ascomata 1.5 to 3 mm wide, opening by means of longitudinal fissures or irregular lobes; ascospores at first single-celled, later becoming 4- to 8-celled	***Therryapini***
–	Ascomata 600 μm × 300 μm, opening by a longitudinal split; ascospores 2- to 4-septate	***Therryatsugae***
55	Ascospores septate	**56**
–	Ascospores aseptate	**57**
56	Ascospores broad-elliptic to oval	***Colpomaagathidis***
–	Ascospores fili-fusiform	***Colpomaintermedium***
57	The internal matrix consisting of brown hyphae; the slit flanked by a dense fringe of colorless gelatinous branched hyphae	***Colpomadensta***
–	The internal matrix absent	**58**
58	A wider and rather complex region layered and branched region near the split lined with pale lip cells	***Colpomacrispum***
–	No such structure at the split of the covering stroma	**59**
59	Ascospores short, ≤ 35 μm	***Colpomapseudographioides***
–	Ascospores long, ≥ 70 μm	**60**
60	Ascomata densely scattered, with a margin that is white or whitish-gray crenate, black, with an ellipsoid to subrotund or irregularly elongated and flexuous disc, almost flat, gray or sub-violaceous	***Colpomaserrulatum***
–	Ascomata seated on a thin black crust, irregular, elliptical or oblong, rugose, black, at length widely gaping or even suborbicular	***Colpomamorbidum***
61	Ascospores 22–27 × 6–7 μm, oblong-elliptic to fusoid-elliptic, often slightly constricted near center, nonseptate, surrounded by a 4–6 μm thick gelatinous sheath	***Bivallumpodocarpi***
–	Ascospores 11–13 × 4–5 μm, oblong-ellipsoidal to slightly fusiform-ellipsoidal, hyaline, aseptate, with 3–5 μm thick gelatinous sheaths	***Bivallumpanamense***
62	Paraphyses sometimes branched, not swollen or slightly swollen at apex	***Hypodermacunninghamiicola***
–	Paraphyses simple, not branched or swollen	**63**
63	Ascospores narrow-fusoid, aseptate, slightly curved	***Hypodermaabietinum***
–	Ascospores rod-like, hyaline, aseptate in early stage, becoming one-septate at maturity	***Hypodermashimanense***
64	Paraphyses conspicuously swollen to 2–3 μm diam. at their tips; ascospores 100–125 × 3–4 μm	***Labivaliduscunninghamiae***
–	Paraphyses not swollen or slightly swollen at their tips; ascospores100–125 × 3–4 μm	***Labivalidusjianchuanensis***
65	Ascomata dull to shiny, dark gray to black, subcuticular, subhymenium flat, consisting of textura angularis	***Lophodermiumagathidis***
–	Ascomata slightly shiny, brown, irregular intraepidermal, subhymenium depressed, consisting of textura porrecta	***Lophodermiumcephalotaxi***

## Supplementary Material

XML Treatment for Abiomyces

XML Treatment for Abiomyces laojunshanensis

XML Treatment for Abiomyces

XML Treatment for Cryptococcomyces

XML Treatment for Cryptococcomyces carbostomaticus

XML Treatment for Cryptococcomyces crystallinus

XML Treatment for Cryptococcomyces juniperi

XML Treatment for Cryptococcomyces niger

XML Treatment for Cryptococcomyces occultus

XML Treatment for Cryptococcomyces

XML Treatment for Cryptococcomyces

XML Treatment for Hypoderma

XML Treatment for Hypoderma cunninghamiicola

XML Treatment for Hypohelion

XML Treatment for Hypohelion shennongjianum

XML Treatment for Labivalidus

XML Treatment for Labivalidus cunninghamiae

XML Treatment for Labivalidus jianchuanensis

XML Treatment for Neotherrya

XML Treatment for Neotherrya abieticola

XML Treatment for Neotherrya catilliformis

XML Treatment for Neotherrya circinata

XML Treatment for Neotherrya nematoidea

XML Treatment for Neotherrya pinicola

XML Treatment for Pseudococcomyces

XML Treatment for Pseudococcomyces yunnanensis

XML Treatment for Stipamyces

XML Treatment for Stipamyces massonianae

XML Treatment for Stipamyces pinicola

XML Treatment for Therrya

XML Treatment for Therrya guizhouensis

XML Treatment for Therrya pinicola

XML Treatment for Therrya strobi

XML Treatment for Tryblidiopsis

XML Treatment for Tryblidiopsis changbaishanensis

XML Treatment for Tryblidiopsis melanostroma

XML Treatment for Tryblidiopsis multiseptata

XML Treatment for Tryblidiopsis yunnanensis

XML Treatment for Tryblidiopsis

XML Treatment for Tryblidiopsis

## ﻿Additional information

### Author ORCIDs

Lan Zhuo https://orcid.org/0000-0002-8129-2812

Hai-Qi Wang https://orcid.org/0009-0004-3388-6263

Peng Zhang https://orcid.org/0009-0007-3113-1838

Xiao-Nan Sui https://orcid.org/0009-0005-3840-3969

Mei-Jun Guo https://orcid.org/0000-0002-8387-8147

Shi-Juan Wang https://orcid.org/0009-0004-1711-4394

Cheng-Lin Hou https://orcid.org/0000-0001-8162-5560

### Data availability

The fungal type materials and type strains of the new species proposed in this study were deposited at Herbarium of the College of Life Science, Capital Normal University, Beijing, China (BJTC) and Capital Normal University Culture Collection Center (CNUCC) with the accession numbers provided in Suppl. material 1: table S1.

## Supplementary materials

Supplementary material 1 Supplementary table and figures https://doi.org/10.3897/imafungus.16.138790.suppl1 Data type doc Explanation note **table S1**: Taxa of *Rhytismatales* used for the phylogenetic analysis, GenBank/UNITE accession numbers, and voucher information. The newly generated sequences in the context of the present study are indicated in **bold**. Notes: HOU: Collecting number; CNUCC: Isolate number; “—” sequences are not available; Species name ^T^ – Type species; Species name ^H^ – Holotype. **fig. S1**: Phylogenetic tree generated by ML analysis based on sequences of the ITS. *Cudoniellaclavus* (AFTOL-ID 166) and *Peziculacarpinea* (KUS-F51029) were selected as outgroups. Maximum likelihood bootstrap values (MLB ≥ 70%) were shown at the nodes. **fig. S2**: Phylogenetic tree generated by ML analysis based on sequences of the LSU. *Cudoniellaclavus* (AFTOL-ID 166) and *Peziculacarpinea* (KUS-F51029) were selected as outgroups. Maximum likelihood bootstrap values (MLB ≥ 70%) were shown at the nodes. **fig. S3**: Phylogenetic tree generated by ML analysis based on sequences of the mtSSU. *Cudoniellaclavus* (AFTOL-ID 166) and *Peziculacarpinea* (KUS-F51029) were selected as outgroups. Maximum likelihood bootstrap values (MLB ≥ 70%) were shown at the nodes. **fig. S4**: Phylogenetic tree generated by ML analysis based on sequences of the LSU and mtSSU. *Cudoniellaclavus* (AFTOL-ID 166) and *Peziculacarpinea* (KUS-F51029) were selected as outgroups. Maximum likelihood bootstrap values (MLB ≥ 70%) were shown at the nodes. https://binary.pensoft.net/file/1253041This dataset is made available under the Open Database License (http://opendatacommons.org/licenses/odbl/1.0/). The Open Database License (ODbL) is a license agreement intended to allow users to freely share, modify, and use this Dataset while maintaining this same freedom for others, provided that the original source and author(s) are credited.Lan Zhuo, Hai-Qi Wang, Peng Zhang, Xiao-Nan Sui, Mei-Jun Guo, Shi-Juan Wang, Cheng-Lin Hou
