# Supplementary material for: ﻿New genera and species of coniferous twig-inhabiting Rhytismatales from China
Source: IMA Fungus. 2025 Feb 17;16:e138790. doi: 10.3897/imafungus.16.138790 (PMC11882027; doi:10.3897/imafungus.16.138790)
Supplement: Supplementary material 1 — Supplementary table and figures [file imafungus-16-e138790-s001.doc]

**Supplementary materials**

**Supplementary Table 1** Taxa of *Rhytismatales* used for the phylogenetic analysis, GenBank/UNITE accession numbers, and voucher information. The newly generated sequences in the context of the present study are indicated in **bold**.

| **Species** | **Voucher** | | **ITS** | **LSU** | | **mtSSU** | | **References** | |  |
| --- | --- | --- | --- | --- | --- | --- | --- | --- | --- | --- |
| ***Abiomyces laojunshanensis*** | **HOU 2064** | | **PQ618623** | **PQ618714** | | **PQ618760** | | **This study** | |  |
| ***Abiomyces laojunshanensis*** | **HOU 2113** | | **PQ618624** | **PQ618715** | | **PQ618761** | | **This study** | |  |
| ***Abiomyces laojunshanensis*** | **HOU 2070** | | **PQ618625** | **PQ618716** | | **PQ618762** | | **This study** | |  |
| ***Abiomyces* sp.** | **HOU 1608ZI** | | **PQ618626** | **PQ618717** | | **PQ618763** | | **This study** | |  |
| *Bifusella camelliae* | HOU 701B | | KF797436 | KF797448 | | KF797459 | | Wang et al. (2014a) | |  |
| *Bifusella linearis* ^T^ | EBJul30-5 | | KT000193 | – | | – | | Kirk et al. (2015) | |  |
| *Bivallum zelandicum* ^T^ | PDD 112248 | | MH921846 | – | | – | | Johnston et al. (2019) | |  |
| *Coccomyces anhuiensis* | HOU 1325 | | MK371316 | MK371317 | | MK371318 | | Lv & Hou (2019) | |  |
| *Coccomyces anhuiensis* ^H^ | HOU 1265 | | MK371313 | MK371314 | | MK371315 | | Lv & Hou (2019) | |  |
| *Coccomyces australis* | HOU 656 | | KF797434 | KF797446 | | KF797457 | | Wang et al. (2014a) | |  |
| *Coccomyces circinatus* | T 72 | | GU138722 | – | | – | | GenBank (2024) | |  |
| *Coccomyces clavatus* | PDD 112233 | | MH578536 | – | | – | | Lv et al. (2019b) | |  |
| *Coccomyces crystalligerus* | ICMP 17375 | | – | HM140501 | | HM143778 | | Lantz et al. (2011) | |  |
| *Coccomyces cunninghamiae* | L4447 | | OR669146 | OR669148 | | OR669150 | | Zheng et al. (2024) | |  |
| *Coccomyces cunninghamiae* | L4551 | | OR669147 | OR669149 | | OR669151 | | Zheng et al. (2024) | |  |
| *Coccomyces cupressini* | ICMP 18318 | | – | HM140502 | | HM143779 | | Lantz et al. (2011) | |  |
| *Coccomyces delta* | R 68 | | GU138731 | – | | – | | Lv et al. (2019b) | |  |
| *Coccomyces dentatus* | HOU 1104 | | KF797433 | KF797445 | | KF797456 | | Wang et al. (2014a) | |  |
| *Coccomyces fagicola* ^H^ | HOU 1366 | | MN385774 | MN372085 | | MN385767 | | Lv et al. (2019b) | |  |
| *Coccomyces fanjingshanensis* | HOU 1493 | | MT123016 | MT122763 | | MT123012 | | Zang et al. (2020) | |  |
| *Coccomyces fodingshanensis* | HOU 1521 | | ON887152 | ON887154 | | ON887153 | | Pu et al. (2022) | |  |
| *Coccomyces globosus* | ICMP 17341 | | – | HM140503 | | HM143780 | | Lantz et al. (2011) | |  |
| *Coccomyces huangshanensis* | R86c | | GU138733 | – | | – | | Lv et al. (2019b) | |  |
| *Coccomyces lauraceus* | ICMP 18319 | | – | HM140504 | | HM143781 | | Lantz et al. (2011) | |  |
| *Coccomyces ledi* | Lantz 386 (UPS) | | – | HM140505 | | HM143782 | | Lantz et al. (2011) | |  |
| *Coccomyces leptideus* | Lantz 393 (UPS) | | – | HM140506 | | HM143783 | | Lantz et al. (2011) | |  |
| *Coccomyces libocedri* | ICMP 18320 | | – | HM140507 | | HM143784 | | Lantz et al. (2011) | |  |
| *Coccomyces mucronatus* | HOU 1497 | | PP488559 | PP488671 | | PP488766 | | Guo et al. (2024) | |  |
| *Coccomyces multangularis* | W9 | | GU138728 | – | | – | | Jia (2011) | |  |
| *Coccomyces libocedri* | ICMP 18320 | | – | HM140507 | | HM143784 | | Lantz et al. (2011) | |  |
| *Coccomyces mucronatus* | HOU 1497 | | PP488559 | PP488671 | | PP488766 | | Guo et al. (2024) | |  |
| *Coccomyces multangularis* | W9 | | GU138728 | – | | – | | Jia (2011) | |  |
| *Coccomyces pycnophyllocladi* | ICMP 17376 | | NR_175748 | NG_081313 | | – | | Crous et al. (2021) | |  |
| *Coccomyces quercicola* | HOU 1367 | | MN385775 | MN372090 | | MN385765 | | Lv et al. (2019b) | |  |
| *Coccomyces shennongjiaensis* | HOU 1365C.1 | | MN385776 | MN385769 | | MN385768 | | Lv et al. (2019b) | |  |
| *Coccomyces sinensis* | Cj01 | | AB787513 | – | | – | | Matsukura et al. (2017) | |  |
| *Coccomyces strobe* | 202.91 | | OQ694432 | – | | – | | GenBank (2024) | |  |
| *Coccomyces strobe* | AFTOL-ID 1250 | | – | DQ470975 | | FJ190622 | | Spatafora et al. (2006) | |  |
| *Coccomyces strobe* | DAOMC251575 | | MH457129 | MH457155 | | – | | McMullin et al. (2019) | |  |
| *Coccomyces strobe* | DAOMC251589 | | MH457130 | MH457157 | | – | | McMullin et al. (2019) | |  |
| *Coccomyces strobe* | DAOMC251937 | | MH457131 | MH457156 | | – | | McMullin et al. (2019) | |  |
| *Coccomyces strobe* | NB-641A | | MH457133 | – | | – | | McMullin et al. (2019) | |  |
| *Coccomyces strobe* | NB-645C | | MH457134 | – | | – | | McMullin et al. (2019) | |  |
| *Coccomyces triangularis* | MUOB 366039 | | OK376742 | – | | – | | GenBank (2024) | |  |
| *Coccomyces tumidus* ^T^ | Lantz 396 | | – | HM140510 | | HM143787 | | Lantz et al. (2011) | |  |
| *Coccomyces yunnanensis* | HOU 1603A | | PP488560 | – | | PP488767 | | Guo et al. (2024) | |  |
| *Colpoma junipericola* | CBS 148247 | | NR_175216 | NG_081325 | | – | | Crous et al. (2021) | |  |
| *Colpoma ledi* | Lantz 379 | | – | HM140512 | | HM143788 | | Lantz et al. (2011) | |  |
| *Colpoma quercinum* ^T^ | Lantz 368 | | – | HM140513 | | HM143789 | | Lantz et al. (2011) | |  |
| ***Cryptococcomyces carbostomaticus*** | **CNUCC 202521** | | **PQ618675** | **–** | | **PQ618804** | | **This study** | |  |
| ***Cryptococcomyces carbostomaticus* ^H^** | **HOU 2025** | | **PQ618627** | **PQ618718** | | **PQ618764** | | **This study** | |  |
| ***Cryptococcomyces crystallinus*** | **HOU 2028** | | **PQ618628** | **PQ618719** | | **PQ618765** | | **This study** | |  |
| ***Cryptococcomyces crystallinus*** | **HOU 2030** | | **PQ618629** | **PQ618720** | | **PQ618766** | | **This study** | |  |
| ***Cryptococcomyces crystallinus*** | **HOU 2062** | | **PQ618630** | **PQ618721** | | **PQ618767** | | **This study** | |  |
| ***Cryptococcomyces crystallinus* ^H^** | **HOU 2082** | | **PQ618631** | **PQ618722** | | **PQ618768** | | **This study** | |  |
| *Cryptococcomyces juniperi* | Lantz 395 (UPS) | | – | HM140511 | | – | | Lantz et al. (2011) | |  |
| ***Cryptococcomyces niger*** | **HOU 2303** | | **PQ618632** | **PQ618723** | | **PQ618769** | | **This study** | |  |
| ***Cryptococcomyces niger* ^H^** | **HOU 1625** | | **PQ618633** | **PQ618724** | | **PQ618770** | | **This study** | |  |
| ***Cryptococcomyces occultus*** | **CNUCC 183822** | | **PQ618676** | **PQ618696** | | **PQ618805** | | **This study** | |  |
| ***Cryptococcomyces occultus*** | **HOU 1613A** | | **PQ618634** | **PQ618725** | | **PQ618771** | | **This study** | |  |
| ***Cryptococcomyces occultus*** | **CNUCC 186111** | | **PQ618677** | **–** | | **–** | | **This study** | |  |
| ***Cryptococcomyces occultus* ^T, H^** | **HOU 1861A** | | **PQ618635** | **PQ618726** | | **PQ618772** | | **This study** | |  |
| ***Cryptococcomyces sp. 1*** | **HOU 1835** | | **PQ618636** | **PQ618727** | | **PQ618773** | | **This study** | |  |
| ***Cryptococcomyces sp. 1*** | **CNUCC 183523** | | **PQ618678** | **PQ618697** | | **PQ618806** | | **This study** | |  |
| ***Cryptococcomyces sp. 2*** | **CNUCC 17821** | | **PQ618679** | **PQ618698** | | **PQ618807** | | **This study** | |  |
| ***Cryptococcomyces sp. 2*** | **CNUCC 2061B31** | | **PQ618680** | **PQ618699** | | **PQ618808** | | **This study** | |  |
| ***Cryptococcomyces sp. 2*** | **HOU 2031A** | | **PQ618637** | **–** | | **PQ618774** | | **This study** | |  |
| *Cryptomyces theae* ^T^ | FU30017 | | KF797432 | KF797444 | | – | | Wang et al. (2014a) | |  |
| *Cudonia circinans* ^T^ | Lantz 402 | | – | HM140515 | | HM143791 | | Lantz et al. (2011) | |  |
| *Cudoniella clavus* | AFTOL-ID 166 | | DQ491502 | DQ470944 | | FJ713604 | | Spatafora et al. (2006) | |  |
| *Davisomycella medusa* | BPI842078 | | AY465525 | – | | – | | Ganley et al. (2004) | |  |
| *Densorhytisma anhuiense* | HOU 1426 | | OQ944136 | OQ944281 | | OQ944322 | | Wang et al. (2023) | |  |
| *Densorhytisma huangshanense* ^T, H^ | HOU 564 | | GQ253101 | FJ495192 | | – | | Hou et al. (2010) | |  |
| *Elytroderma deformans* ^T^ | CBS 181.68 | | AF203469 | – | | – | | Ortiz-García et al. (2003) | |  |
| *Fanglania hubeiensis* ^T, H^ | HOU 1406 | | OQ944273 | OQ944311 | | OQ944352 | | Wang et al. (2023) | |  |
| *Fanglania parasiticum* ^H^ | HOU 1417 | | OQ944274 | OQ944312 | | OQ944353 | | Wang et al. (2023) | |  |
| *Hypoderma aliforme* ^H^ | ICMP 17379 | | NR_175749 | NG_081314 | | – | | Crous et al. (2021) | |  |
| *Hypoderma berberidis* | HOU 892 | | JX232414 | JX232420 | | KF813010 | | Wang et al. (2013) | |  |
| *Hypoderma campanulatum* | ICMP 17383 | | – | HM140517 | | HM143792 | | Lantz et al. (2011) | |  |
| *Hypoderma caricis* | R 17 | | GU138752 | – | | – | | Wang et al. (2013) | |  |
| *Hypoderma carinatum* | ICMP 18322 | | – | HM140518 | | HM143793 | | Lantz et al. (2011) | |  |
| *Hypoderma commune* | Hanson 2006-451(UPS) | | – | HM140519 | | HM143794 | | Lantz et al. (2011) | |  |
| *Hypoderma cordylines* | ICMP 17344 | | – | HM140521 | | HM143796 | | Lantz et al. (2011) | |  |
| *Hypoderma hederae* | Lantz & Minter 421 (UPS) | | – | HM140522 | | HM143797 | | Lantz et al. (2011) | |  |
| *Hypoderma junipericola* | R104a | | GU138754 | – | | – | | GenBank (2024) | |  |
| *Hypoderma liliense* | ICMP 18323 | | – | HM140523 | | HM143798 | | Lantz et al. (2011) | |  |
| *Hypoderma minteri* ^H^ | BJTC 201203 | | NR_120173 | NG_068735 | | – | | Wang et al. (2013) | |  |
| *Hypoderma obtectum* | ICMP 17365 | | – | HM140525 | | HM143800 | | Lantz et al. (2011) | |  |
| *Hypoderma paralinderae* | GZAAS 19-0104 | | MN638873 | MN638878 | | — | | Zhang et al. (2020) | |  |
| *Hypoderma rubi* ^T^ | ICMP 18325 | | – | HM140527 | | HM143802 | | Lantz et al. (2011) | |  |
| *Hypoderma siculum* | PDD 99894 | | JF683424 | – | | – | | Lantieri et al. (2012) | |  |
| ***Hypoderma cunninghamiicola*** | **L4647** | | **PQ618638** | **PQ618728** | | **PQ618775** | | **This study** | |  |
| ***Hypoderma cunninghamiicola*** | **L4648** | | **PQ618639** | **PQ618729** | | **–** | | **This study** | |  |
| *Hypoderma stephanandrae* | R63 | | GU138753 | – | | – | | Wang et al. (2014a) | |  |
| *Hypoderma sticheri* | ICMP 17353 | | – | HM140529 | | HM143804 | | Lantz et al. (2011) | |  |
| *Hypohelion anhuiense* | HOU1089 | | KF797430 | KF797442 | | KF797454 | | Wang et al. (2014a) | |  |
| *Hypohelion anhuiense* ^H^ | HOU1078 | | KF797431 | KF797443 | | KF797455 | | Wang et al. (2014a) | |  |
| *Hypohelion durum* | HOU 524 | | KF797429 | KF797441 | | KF797453 | | Wang et al. (2014a) | |  |
| *Hypohelion scirpinum* ^T^ | Lantz 394 | | – | HM140531 | | HM143806 | | Lantz et al. (2011) | |  |
| ***Hypohelion shennongjianum*** | **HOU 1342A** | | **PQ618640** | **PQ618730** | | **PQ618776** | | **This study** | |  |
| ***Hypohelion shennongjianum*** | **CNUCC 1342A31** | | **PQ618681** | **PQ618700** | | **PQ618809** | | **This study** | |  |
| *Johnstoniella yunnanensis* ^T, H^ | HOU 943 | | OQ944275 | OQ944313 | | OQ944354 | | Wang et al. (2023) | |  |
| ***Labivalidus cunninghamiae*** | **HOU 2166** | | **PQ618641** | **PQ618731** | | **PQ618777** | | **This study** | |  |
| ***Labivalidus cunninghamiae*** | **CNUCC 216621** | | **PQ618682** | **PQ618701** | | **PQ618810** | | **This study** | |  |
| ***Labivalidus cunninghamiae*** | **CNUCC 216611** | | **PQ618683** | **PQ618702** | | **PQ618811** | | **This study** | |  |
| ***Labivalidus cunninghamiae*** | **CNUCC 2173A21** | | **PQ618684** | **PQ618703** | | **PQ618812** | | **This study** | |  |
| ***Labivalidus cunninghamiae*** | **CNUCC 2173A22** | | **PQ618685** | **PQ618704** | | **PQ618813** | | **This study** | |  |
| ***Labivalidus cunninghamiae* ^H^** | **HOU 2173A** | | **PQ618642** | **PQ618732** | | **PQ618778** | | **This study** | |  |
| ***Labivalidus jianchuanensis*** | **CNUCC 1781B11** | | **PQ618686** | **–** | | **PQ618814** | | **This study** | |  |
| ***Labivalidus jianchuanensis*** | **HOU 2024** | | **PQ618643** | **–** | | **–** | | **This study** | |  |
| ***Labivalidus jianchuanensis*** | **HOU 1781** | | **PQ618644** | **PQ618733** | | **PQ618779** | | **This study** | |  |
| ***Labivalidus jianchuanensis*** | **HOU 2023** | | **PQ618645** | **–** | | **–** | | **This study** | |  |
| ***Labivalidus jianchuanensis*** | **CNUCC 1815A22** | | **PQ618687** | **PQ618705** | | **PQ618815** | | **This study** | |  |
| ***Labivalidus jianchuanensis*** | **HOU 1815A** | | **PQ618646** | **PQ618734** | | **–** | | **This study** | |  |
| *Lirula exigua* | HOU 475A | | HQ902157 | HQ902150 | | – | | Fan et al. (2012) | |  |
| *Lirula yunnanensis* | HOU 464A | | HQ902156 | HQ902149 | | – | | Fan et al. (2012) | |  |
| *Lophodermella arcuata* | RMNP LU1 | | MN937644 | MN937585 | | – | | Ata et al. (2021) | |  |
| *Lophodermella concolor* | LP7C | | MN937621 | MN937588 | | – | | Ata et al. (2021) | |  |
| *Lophodermella conjuncta* | PHP19-0987 | | MN937608 | MN937603 | | – | | Ata et al. (2021) | |  |
| *Lophodermella montivaga* | TC9M | | MN937629 | MN937599 | | – | | Ata et al. (2021) | |  |
| *Lophodermella sulcigena* ^T^ | PH18-0656 | | MN937624 | MN937604 | | – | | Ata et al. (2021) | |  |
| *Lophodermiopsis rhododendri* ^H^ | HOU 1588A | | PP488564 | PP488675 | | – | | Guo et al. (2024) | |  |
| *Lophodermiopsis splendida* ^H^ | HOU 936B | | PP488570 | PP488680 | | PP488773 | | Guo et al. (2024) | |  |
| *Lophodermiopsis tumida* ^H^ | HOU 1768 | | PP488572 | PP488682 | | PP488775 | | Guo et al. (2024) | |  |
| *Lophodermiopsis yunnanensis* ^H^ | HOU 438B | | PP488577 | PP488687 | | PP488780 | | Guo et al. (2024) | |  |
| *Lophodermium actinothyrium* | losa139 | | AY100663 | – | | – | | Ortiz-García et al. (2003) | |  |
| *Lophodermium agathidis* | ICMP:18327 | | – | HM140534 | | HM143810 | | Lantz et al. (2011) | |  |
| *Lophodermium agathidis* | ICMP:14598 | | – | HM140532 | | HM143808 | | Lantz et al. (2011) | |  |
| *Lophodermium arundinaceum* ^T^ | Lantz 323 | | – | HM140535 | | HM143811 | | Lantz et al. (2011) | |  |
| *Lophodermium australe* | isolate 24 | | EU696778 | – | | – | | Toju et al. (2013) | |  |
| *Lophodermium autumnale* | HOU 475C | | HQ902158 | HQ902151 | | – | | Fan et al. (2012) | |  |
| *Lophodermium baculiferum* | mon2zem Nuevo Leon | | AY100656 | – | | – | | Ortiz-García et al. (2003) | |  |
| *Lophodermium brunneolum* | ICMP 17373 | | – | HM140536 | | HM143812 | | Lantz et al. (2011) | |  |
| *Lophodermium cathayae* | Hou 831M | | HQ992812 | – | | – | | Gao et al. (2013) | |  |
| *Lophodermium cedrinum* | BCAB212 | | MG779472 | – | | – | | GenBank (2024) | |  |
| *Lophodermium cephalotaxi* | T15 | | GU138701 | – | | – | | GenBank (2024) | |  |
| *Lophodermium* cf. *pinastri* | Lantz & Williams 417 (UPS) | | – | HM140553 | | – | | Lantz et al. (2011) | |  |
| *Lophodermium conigenum* | Lantz 410 (UPS) | | – | HM140537 | | HM143813 | | Lantz et al. (2011) | |  |
| *Lophodermium corconticum* | SY370 | | OK493168 | – | | – | | Lantz et al. (2011) | |  |
| *Lophodermium culmigenum* | Lantz 430 (UPS) | | – | HM140539 | | HM143815 | | Lantz et al. (2011) | |  |
| *Lophodermium eucalypti* | ICMP 16796 | | – | HM140541 | | HM143817 | | Lantz et al. (2011) | |  |
| *Lophodermium fissuratum* | CA2PM1-19-1 | | KY576859 | – | | – | | Salas‐Lizana & Oono (2018) | |  |
| *Lophodermium gamundiae* ^H^ | ICMP 16802 | | NR_119628 | – | | – | | GenBank (2024) | |  |
| *Lophodermium gramineum* | Lantz 441 (UPS) | | – | HM140542 | | – | | Lantz et al. (2011) | |  |
| *Lophodermium hauturuanum* | PDD 112227 | | MH578524 | – | | – | | Johnston et al. (2019) | |  |
| *Lophodermium herbarum* | Lantz 439 (UPS) | | – | HM140543 | | HM143818 | | Lantz et al. (2011) | |  |
| *Lophodermium implicatum* | T9 | | GU138700 | – | | – | | GenBank (2024) | |  |
| *Lophodermium indianum* | CMW 39125 | | KF636510 | – | | – | | Tanney & Seifert (2017) | |  |
| *Lophodermium jiangnanense* | Cj12 | | AB787524 | – | | – | | Matsukura et al. (2017) | |  |
| *Lophodermium johnstonii* | isolate 310 | | JX232417 | – | | – | | Wang et al. (2013) | |  |
| *Lophodermium juniperinum* | Lantz 306 (UPS) | | – | HM140544 | | HM143819 | | Lantz et al. (2011) | |  |
| *Lophodermium macci* | wxm130 | | HM037981 | – | | – | | GenBank (2024) | |  |
| *Lophodermium medium* | ICMP 17360 | | – | HM140545 | | HM143820 | | Lantz et al. (2011) | |  |
| *Lophodermium melaleucum* | Lantz 378 (UPS) | | – | HM140546 | | HM143821 | | Lantz et al. (2011) | |  |
| *Lophodermium microsporum* | MFLU 15-3100 | | MK584994 | – | | – | | Ekanayaka et al. (2019) | |  |
| *Lophodermium molitoris* | CBS 597.84 | | AY100659 | – | | – | | Ortiz-García et al. (2003) | |  |
| *Lophodermium nitens* | Sie1PL2-14-1 | | MG877536 | – | | – | | Salas‐Lizana & Oono (2018) | |  |
| *Lophodermium nitidum* | Lantz 435 (UPS) | | – | HM140547 | | HM143822 | | Lantz et al. (2011) | |  |
| *Lophodermium oxycocci* | Lantz 397 (UPS) | | – | – | | HM143823 | | Lantz et al. (2011) | |  |
| *Lophodermium paeoniae* | Lundqvist 21693 | | – | HM140549 | | – | | Lantz et al. (2011) | |  |
| *Lophodermium petiolicola* | Lantz 385 (UPS) | | – | HM140550 | | HM143824 | | Lantz et al. (2011) | |  |
| *Lophodermium petrakii* | R60 | | FJ861984 | – | | – | | Tanney & Seifert (2017) | |  |
| *Lophodermium piceae* | Lantz 317 (UPS) | | – | HM140551 | | HM143825 | | Lantz et al. (2011) | |  |
| *Lophodermium pinastri* | Lantz 437 | | – | HM140552 | | HM143826 | | Lantz et al. (2011) | |  |
| *Lophodermium pini-bungeanae* | R112 | | FJ861989 | – | | – | | Tanney & Seifert (2017) | |  |
| *Lophodermium pini-excelsae* | R85 | | FJ861987 | – | | – | | Tanney & Seifert (2017) | |  |
| *Lophodermium pini-mugonis* | 568M | | JF332165 | – | | – | | Hou et al. (2009) | |  |
| *Lophodermium pini-taiwanensis* | HOU 1222B | | KX443656 | – | | – | | Li et al. (2016) | |  |
| *Lophodermium platyplacum* | Lantz & Minter 419 (UPS) | | – | HM140554 | | HM143827 | | Lantz et al. (2011) | |  |
| *Lophodermium rectangulare* | PDD 112235 | | MH578540 | – | | – | | Johnston et al. (2019) | |  |
| *Lophodermium resinosum* ^H^ | DAOMC 251482 | | NR_172257 | NG_060349 | | – | | Tanney & Seifert (2017) | |  |
| *Lophodermium seditiosum* | SY366 | | OK493165 | – | | – | | GenBank (2024) | |  |
| *Lophodermium* sp. | Lantz 443 (UPS) | | – | HM140555 | | HM143828 | | Lantz et al. (2011) | |  |
| *Lophodermium sphaerioides* | Lantz 382 (UPS) | | – | HM140556 | | HM143829 | | Lantz et al. (2011) | |  |
| *Lophodermium thailandicum* | MFLU 17-0673 | | MG821634 | – | | – | | Hyde et al. (2018) | |  |
| *Lophodermium tindalii* | PDD 92044 | | MH921867 | – | | – | | Johnston et al. (2019) | |  |
| *Meloderma desmazieri* ^T^ | MD3 (ATCCc) | | AF426056 | – | | – | | Deckert et al. (2002) | |  |
| *Meloderma dracophylli* | ICMP 17343 | | – | HM140561 | | HM143833 | | Lantz et al. (2011) | |  |
| *Nematococcomyces brunneus* ^H^ | HOU 1610A | | PP488578 | PP488688 | | – | | Guo et al. (2024) | |  |
| *Nematococcomyces mirabilis* ^H^ | HOU 1564 | | PP488583 | – | | PP488782 | | Guo et al. (2024) | |  |
| *Nematococcomyces oberwinkleri* ^H^ | HOU 482B | | – | KC312686 | | KC312689 | | Tian et al. (2013) | |  |
| *Nematococcomyces rhododendri* ^T^ | HOU 879A | | – | KC312685 | | KC312690 | | Tian et al. (2013) | |  |
| *Nematococcomyces vermiformis* ^H^ | HOU 1840B | | – | PP488695 | | PP488787 | | Guo et al. (2024) | |  |
| *Nematococcomyces yunnanensis* ^H^ | HOU 1592 | | PP488589 | PP488697 | | PP488788 | | Guo et al. (2024) | |  |
| *Neococcomyces rhododendri* | HOU 1921A | | PP488592 | PP488699 | | PP488791 | | Guo et al. (2024) | |  |
| *Neorhytisma panamense* ^T^ | UCH 5284 | | OQ944277 | – | | OQ944356 | | Wang et al. (2023) | |  |
| *Neotherrya abieticola* | HOU 447A | | KP322574 | KP322580 | | KP322587 | | Zhang et al. (2020) | |  |
| *Neotherrya abieticola* | CNU 447A | | KP322573 | KP322579 | | KP322586 | | Zhang et al. (2020) | |  |
| ***Neotherrya catilliformis*** | **HOU 1608A** | | **PQ618647** | **PQ618735** | | **PQ618780** | | **This study** | |  |
| ***Neotherrya catilliformis*** | **HOU 2304** | | **PQ618648** | **PQ618736** | | **–** | | **This study** | |  |
| ***Neotherrya catilliformis*** | **HOU 2097** | | **PQ618649** | **PQ618737** | | **PQ618781** | | **This study** | |  |
| ***Neotherrya circinata*** | **HOU 1587** | | **PQ618650** | **PQ618738** | | **PQ618782** | | **This study** | |  |
| ***Neotherrya circinata*** | **CNUCC 18241** | | **PQ618688** | **PQ618706** | | **PQ618816** | | **This study** | |  |
| ***Neotherrya circinata*** | **CNUCC 182433** | | **PQ618689** | **PQ618707** | | **PQ618817** | | **This study** | |  |
| ***Neotherrya circinata*** | **CNUCC 182431** | | **PQ618690** | **PQ618708** | | **PQ618818** | | **This study** | |  |
| ***Neotherrya circinata* ^T, H^** | **HOU 883** | | **PQ618651** | **PQ618739** | | **PQ618783** | | **This study** | |  |
| ***Neotherrya nematoidea*** | **HOU 1598B** | | **PQ618652** | **PQ618740** | | **PQ618784** | | **This study** | |  |
| ***Neotherrya nematoidea*** | **HOU 2305** | | **PQ618653** | **PQ618741** | | **PQ618785** | | **This study** | |  |
| ***Neotherrya pinicola*** | **HOU 919** | | **PQ618654** | **PQ618742** | | **PQ618786** | | **This study** | |  |
| *Neotryblidiopsis polygonalis* ^T, H^ | HOU 1823 | | PP488594 | PP488701 | | PP488793 | | Guo et al. (2024) | |  |
| *Neotryblidiopsis yunnanensis* ^H^ | HOU 366 | | PP488601 | PP488706 | | PP488800 | | Guo et al. (2024) | |  |
| *Occultimyces fusiformis* ^T, H^ | HOU 458A | | PP488603 | – | | – | | Guo et al. (2024) | |  |
| *Pezicula carpinea* | KUS-F51029 | | JN033388 | JN086691 | | JN086765 | | Han et al. (2014) | |  |
| *Placuntium andromedae* ^T^ | Lantz 307 (UPS) | | – | HM140564 | | – | | Lantz et al. (2011) | |  |
| *Ploioderma destruens* | T33 | | GU138756 | – | | – | | GenBank (2024) | |  |
| ***Pseudococcomyces yunnanensis*** | **HOU 2306** | | **PQ618655** | **–** | | **PQ618787** | | **This study** | |  |
| ***Pseudococcomyces yunnanensis* ^T, H^** | | **HOU 1574** | **PQ618656** | **PQ618743** | | **PQ618788** | | **This study** | |  |
| *Pseudographis elatina* ^T^ | | GJO 0090016 | MK751794 | MK751803 | | MK751717 | | Karakehian et al. (2019) | |  |
| *Pseudographis pinicola* | | FH NB842 | MK751796 | MK751805 | | MK751719 | | Karakehian et al. (2019) | |  |
| *Rhodohypoderma clavatum* ^H^ | | HOU 927B | PP488605 | – | | PP488803 | | Guo et al. (2024) | |  |
| *Rhodohypoderma cuspidatum* | | HOU 484A | PP488606 | PP488710 | | PP488804 | | Guo et al. (2024) | |  |
| *Rhodohypoderma rhododendri* ^T, H^ | | HOU 1840A | PP488608 | PP488712 | | PP488806 | | Guo et al. (2024) | |  |
| *Rhodohypoderma urniforme* | | HOU 879B | PP488612 | PP488715 | | PP488810 | | Guo et al. (2024) | |  |
| *Rhytisma acerinum* ^T^ | | HOU 1058 | OQ944148 | OQ944295 | | OQ944332 | | Guo et al. (2024) | |  |
| *Septofusella triseptate* ^T, H^ | | HOU 292 | PP488615 | PP488718 | | PP488813 | | Guo et al. (2024) | |  |
| *Septomyces magnus* ^T^ | | HOU 368 | PP488618 | PP488720 | | – | | Guo et al. (2024) | |  |
| *Shiqia yuexiense* ^T^ | | HOU 521 | OQ944155 | OQ944303 | | OQ944340 | | Wang et al. (2023) | |  |
| *Shuqunia clavata* ^H^ | | HOU 1812 | PP488619 | – | | – | | Guo et al. (2024) | |  |
| *Shuqunia longa* ^T, H^ | | HOU 368B | PP488620 | PP488721 | | PP488815 | | Guo et al. (2024) | |  |
| *Shuqunia nitens* ^H^ | | HOU 1758 | PP488622 | PP488723 | | PP488817 | | Guo et al. (2024) | |  |
| *Shuqunia rhododendri* ^H^ | | HOU 1848D | PP488626 | PP488727 | | PP488821 | | Guo et al. (2024) | |  |
| *Shuqunia yunnanensis* ^H^ | | HOU 1567 | PP488628 | PP488729 | | PP488823 | | Guo et al. (2024) | |  |
| *Spathularia flavida* ^T^ | | CBS 399.52 | – | AY541496 | | AY575101 | | Lumbsch et al. (2005) | |  |
| *Sporomega degenerans* ^T^ | | Lantz 367 | – | HM140567 | | HM143839 | | Lantz et al. (2011) | |  |
| *Sporomega nodulifera* ^H^ | | HOU 1629A | PP488631 | PP488731 | | PP488826 | | Guo et al. (2024) | |  |
| ***Stipamyces massonianae* ^T, H^** | | **HOU 1215** | **PQ618657** | **PQ618744** | | **PQ618789** | | **This study** | |  |
| ***Stipamyces pinicola*** | | **HOU 1618** | **PQ618658** | **PQ618745** | | **PQ618790** | | **This study** | |  |
| ***Stipamyces pinicola*** | | **HOU 538** | **PQ618659** | **PQ618746** | | **–** | | **This study** | |  |
| *Stipamyces pinicola* | | HOU 486A | JX317676 | KP322585 | | KP322592 | | Lei et al. (2013) | |  |
| *Terriera camelliicola* | | L0447 | – | KP878552 | | KP878553 | | GenBank (2024) | |  |
| *Terriera cladophila* ^T^ | | Lantz 423 | – | HM140568 | | HM143840 | | Lantz et al. (2011) | |  |
| *Terriera elliptica* ^H^ | | HOU 327 | KP878549 | KP878550 | | KP878551 | | Zhang et al. (2015) | |  |
| *Terriera guizhouensis* | | HOU 1528B | MT534528 | MT549872 | | MT549865 | | Cai et al. (2020) | |  |
| *Terriera houjiazhuangensis* | | HOU 1275B | MT549879 | MT549864 | | MT549884 | | Cai et al. (2020) | |  |
| *Terriera ilicis* | | HOU 1360B | MT549861 | MT549873 | | MT549886 | | Cai et al. (2020) | |  |
| *Terriera illiciicola* | | R89 | FJ861980 | – | | – | | GenBank (2024) | |  |
| *Terriera karsti* ^H^ | | MFLU 18-2288 | MN638876 | MN638881 | | MN638871 | | Zhang et al. (2020) | |  |
| *Terriera meitanensis* ^H^ | | MFLU 18-2299 | MN638874 | MN638879 | | MN638869 | | Zhang et al. (2020) | |  |
| *Terriera minor* | | ICMP 13973 | – | HM140570 | | HM143842 | | Lantz et al. (2011) | |  |
| *Terriera pandanicola* ^H^ | | MFLU 16-1931 | – | MH260320 | | MW334971 | | Zhang et al. (2020) | |  |
| *Terriera rhododendri* ^H^ | HOU 1122 | | PP488634 | | – | | PP488829 | | Guo et al. (2024) | |
| *Terriera sigmoideospora* ^H^ | MFLU 18-2297 | | MN638877 | | MN638882 | | MN638872 | | Zhang et al. (2020) | |
| *Terriera thailandica* ^H^ | MFLUCC 14-0818 | | – | | NG_059726 | | – | | Hyde et al. (2016) | |
| *Therrya eucalypti* | PRJ AU09-82 | | KM880188 | | – | | – | | GenBank (2024) | |
| *Therrya fuckelii* | CBS 377.58 | | JF793672 | | – | | – | | Solheim et al. (2013) | |
| *Therrya fuckelii* | NWFVA4768 | | MT821241 | | – | | – | | GenBank (2024) | |
| *Therrya guizhouensis* | HOU 439A | | JX317677 | | – | | – | | Lei et al. (2013) | |
| *Therrya guizhouensis* | HOU 439B | | JX317678 | | – | | – | | Lei et al. (2013) | |
| *Therrya pini* | CBS 177.56 | | MH857568 | | MH869111 | | KC312688 | | Tian et al. (2013) | |
| *Therrya pini* | 2004-9/13 | | JF793676 | | – | | – | | Solheim et al. (2013) | |
| *Therrya pinicola* | TRY | | MT707244 | | – | | – | | Haelewaters et al. (2020) | |
| *Therrya pinicola* | ATCC:66185 | | UDB035391(from UNITE) | | – | | – | | Haelewaters et al. (2020) | |
| ***Therrya pinicola*** | **HOU 2237** | | **PQ618660** | | **PQ618747** | | **PQ618791** | | **This study** | |
| ***Therrya pinicola*** | **HOU 2238B** | | **PQ618661** | | **PQ618748** | | **PQ618792** | | **This study** | |
| *Triblidium caliciiforme* ^T^ | – | | MK751798 | | MK751807 | | MK751721 | | Karakehian et al. (2019) | |
| *Triblidium hubeiense* ^H^ | HOU 1350A | | MN541813 | | MN541811 | | MN541828 | | Lv et al. (2019a) | |
| *Triblidium laojunshanense* ^H^ | HOU 1620 | | PP488635 | | PP488734 | | PP488830 | | Guo et al. (2024) | |
| *Triblidium rhododendri* ^H^ | HOU 326A | | PP488637 | | PP488736 | | PP488832 | | Guo et al. (2024) | |
| *Triblidium rostriforme* | HOU 1603B | | PP488638 | | PP488737 | | PP488833 | | Guo et al. (2024) | |
| *Triblidium sichuanense* ^H^ | HOU 295 | | PP488639 | | PP488738 | | PP488834 | | Guo et al. (2024) | |
| *Triblidium verrucosum* | UME-29336a | | MK751793 | | MK751802 | | MK751716 | | Lv et al. (2019a) | |
| *Triblidium yunnanense* | HOU 1179 | | MN541814 | | MN541809 | | MN541816 | | Lv et al. (2019a) | |
| ***Tryblidiopsis changbaishanensis*** | **HOU 2211** | | **PQ618662** | | **–** | | **PQ618793** | | **This study** | |
| ***Tryblidiopsis changbaishanensis*** | **CNUCC 221121** | | **PQ618691** | | **PQ618709** | | **PQ618819** | | **This study** | |
| *Tryblidiopsis changbaishanensis* | HOU 662 | | KC312675 | | KC312682 | | KC312695 | | This study | |
| ***Tryblidiopsis changbaishanensis*** | **CNUCC 221011** | | **PQ618692** | | **PQ618710** | | **PQ618820** | | **This study** | |
| ***Tryblidiopsis changbaishanensis* ^H^** | **HOU 2210** | | **PQ618664** | | **PQ618750** | | **PQ618795** | | **This study** | |
| *Tryblidiopsis magnesii* | NB 630 | | MK748209 | | MK748168 | | – | | Tanney & Seifert (2019) | |
| *Tryblidiopsis magnesii* ^H^ | DAOMC 252096 | | NR_164611 | | NG_066463 | | – | | Tanney & Seifert (2019) | |
| ***Tryblidiopsis melanostroma*** | **HOU 2307** | | **PQ618665** | | **PQ618751** | | **–** | | **This study** | |
| ***Tryblidiopsis melanostroma* ^H^** | **HOU 2105** | | **PQ618666** | | **PQ618752** | | **PQ618796** | | **This study** | |
| ***Tryblidiopsis multiseptata*** | **HOU 2107** | | **PQ618667** | | **PQ618753** | | **PQ618797** | | **This study** | |
| ***Tryblidiopsis multiseptata*** | **HOU 2104** | | **PQ618668** | | **PQ618754** | | **PQ618798** | | **This study** | |
| ***Tryblidiopsis multiseptata*** | **HOU 1750** | | **PQ618669** | **PQ618755** | | **PQ618799** | | **This study** | |  |
| ***Tryblidiopsis multiseptata*** | **CNUCC 17502** | | **PQ618693** | **PQ618711** | | **PQ618821** | | **This study** | |  |
| ***Tryblidiopsis multiseptata* ^H^** | **CNUCC 17501** | | **PQ618694** | **PQ618712** | | **PQ618822** | | **This study** | |  |
| *Tryblidiopsis pinastri* ^T^ | Lantz 412 | | – | HM140573 | | – | | Lantz et al. (2011) | |  |
| *Tryblidiopsis sichuanensis* | HOU 300 | | KC312677 | KC312679 | | KC312693 | | Wang et al. (2014b) | |  |
| *Tryblidiopsis sichuanensis* ^H^ | HOU 306 | | KC312676 | KC312683 | | KC312692 | | Wang et al. (2014b) | |  |
| *Tryblidiopsis sinensis* ^H^ | HOU 814 | | KC312674 | KC312681 | | KC312694 | | Wang et al. (2014b) | |  |
| ***Tryblidiopsis sp. 1*** | **HOU 288A** | | **PQ618670** | **PQ618756** | | **–** | | **This study** | |  |
| ***Tryblidiopsis sp. 2*** | **HOU 956** | | **PQ618671** | **–** | | **PQ618800** | | **This study** | |  |
| *Tryblidiopsis yunnanensis* | HOU 1116B | | KU213974 | KU213977 | | KU213979 | | GenBank (2024) | |  |
| ***Tryblidiopsis yunnanensis*** | **HOU 1746** | | **PQ618673** | **PQ618758** | | **PQ618802** | | **This study** | |  |
| ***Tryblidiopsis yunnanensis*** | **CNUCC 174621** | | **PQ618695** | **PQ618713** | | **PQ618823** | | **This study** | |  |
| *Tryblidiopsis yunnanensis* ^H^ | HOU 487 | | KU213973 | KU213976 | | KU213978 | | GenBank (2024) | |  |
| *Xyloma filamentosum* ^H^ | HOU 1584 | | NR154388 | NG_060060 | | – | | Masumoto et al. (2015) | |  |
| *Xyloma golobosum* | HOU 898 | | OQ944279 | OQ944316 | | OQ944358 | | Wang et al. (2023) | |  |
| *Xyloma maximus* | Lantz 424 | | – | HM140514 | | HM143790 | | Lantz et al. (2011) | |  |
| *Yingrenia cannonii* ^H^ | HOU 1797 | | PP488642 | PP488740 | | – | | Guo et al. (2024) | |  |
| *Yingrenia debililabiata* ^H^ | HOU 1604B | | PP488643 | – | | PP488836 | | Guo et al. (2024) | |  |
| *Yingrenia debililabioides* ^H^ | HOU 444 | | PP488644 | PP488741 | | PP488837 | | Guo et al. (2024) | |  |
| *Yingrenia erumpens* ^T^ | HOU 438A | | PP488648 | PP488744 | | PP488840 | | Guo et al. (2024) | |  |
| *Yingrenia laojunshanensis* ^H^ | HOU 1593B | | PP488649 | – | | – | | Guo et al. (2024) | |  |
| *Yingrenia papillata* ^H^ | HOU 845A | | PP488654 | PP488748 | | PP488844 | | Guo et al. (2024) | |  |
| *Yingrenia rhododendri* ^H^ | HOU 1847A | | PP488655 | PP488749 | | PP488845 | | Guo et al. (2024) | |  |
| *Yingrenia yunnanensis* ^H^ | HOU 1570 | | PP488658 | PP488752 | | – | | Guo et al. (2024) | |  |

Notes：HOU: Collecting number; CNUCC: Isolate number; “—” sequences are not available; Species name ^T^ – Type species; Species name ^H^ – Holotype.

**
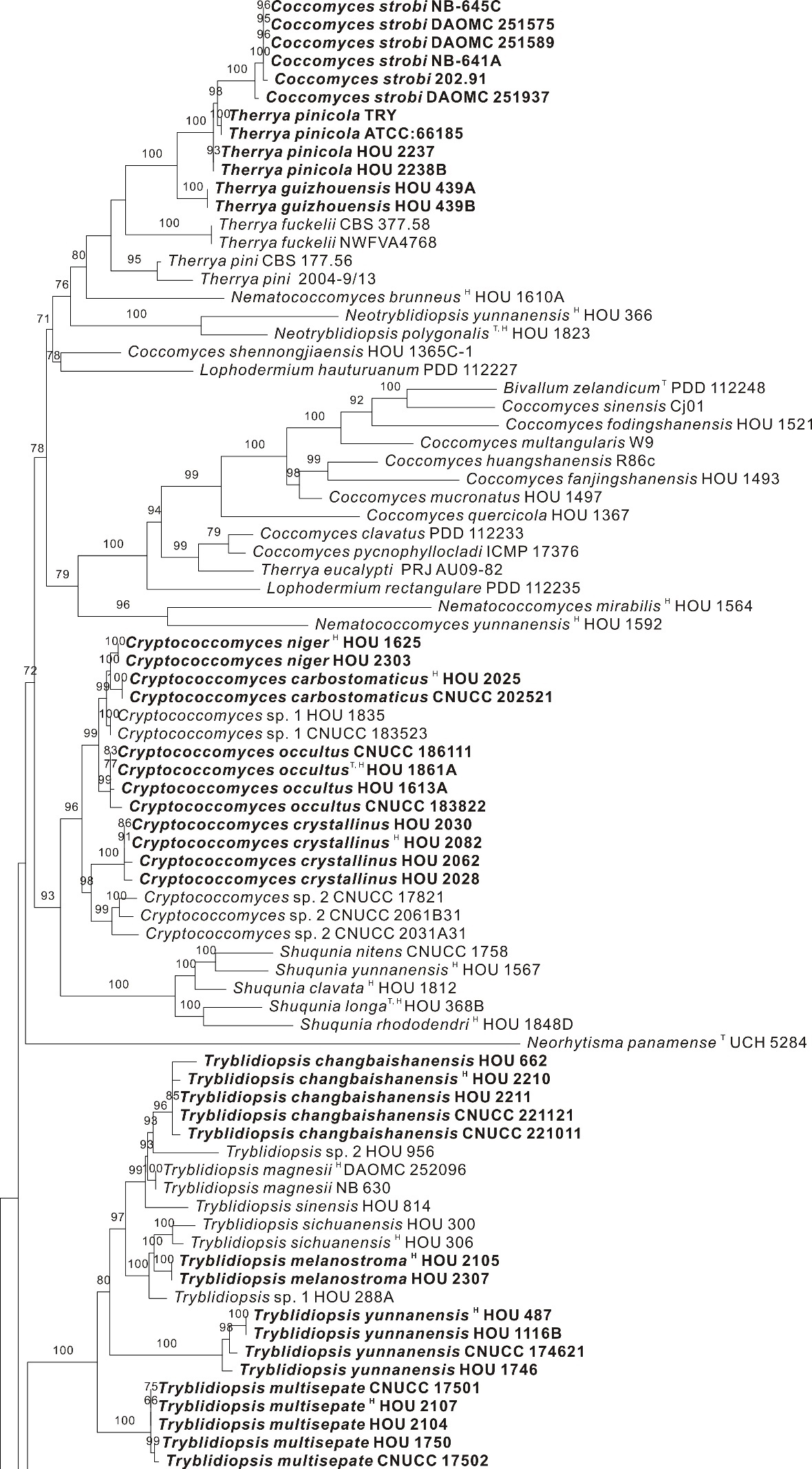
**


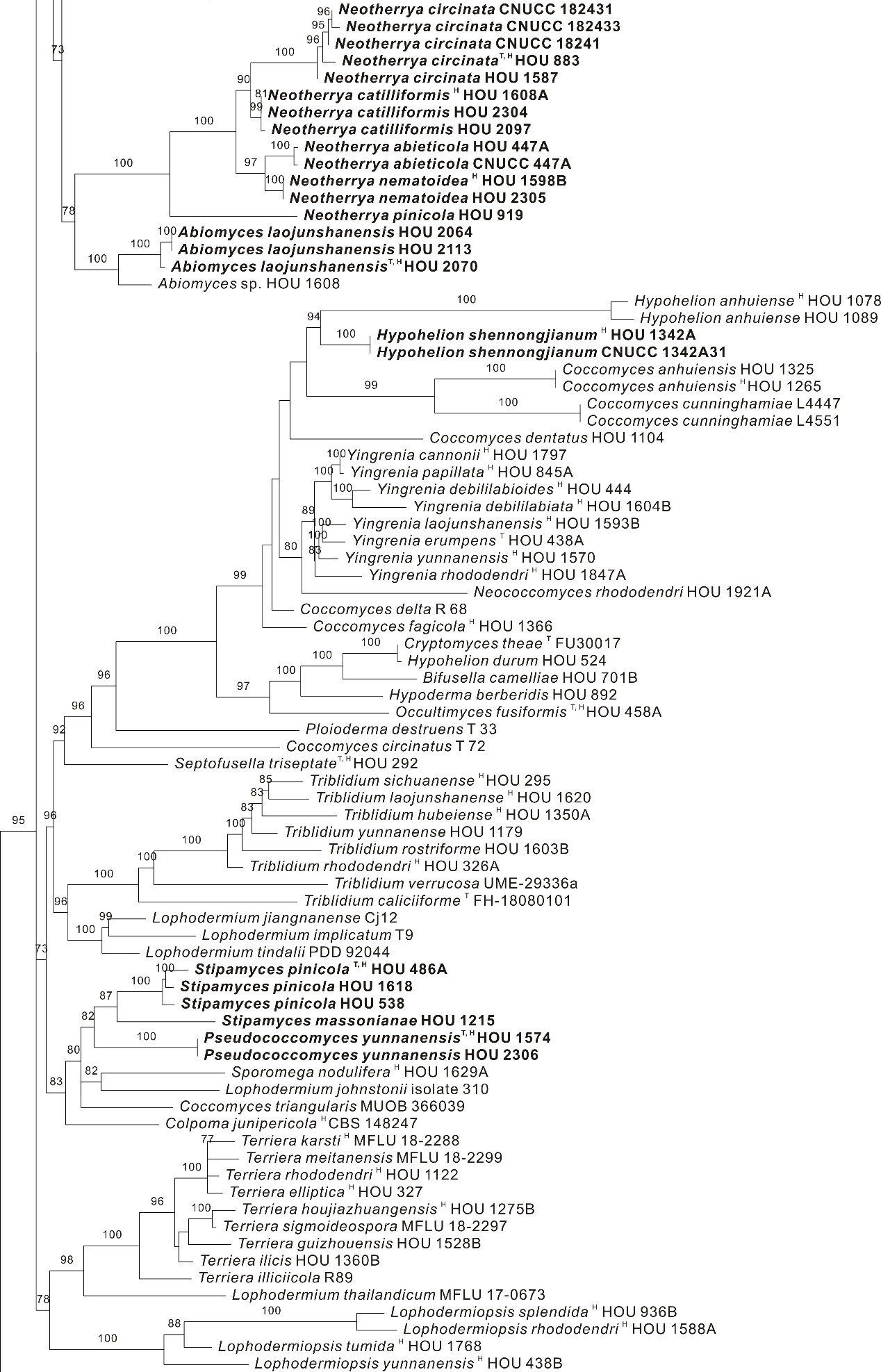


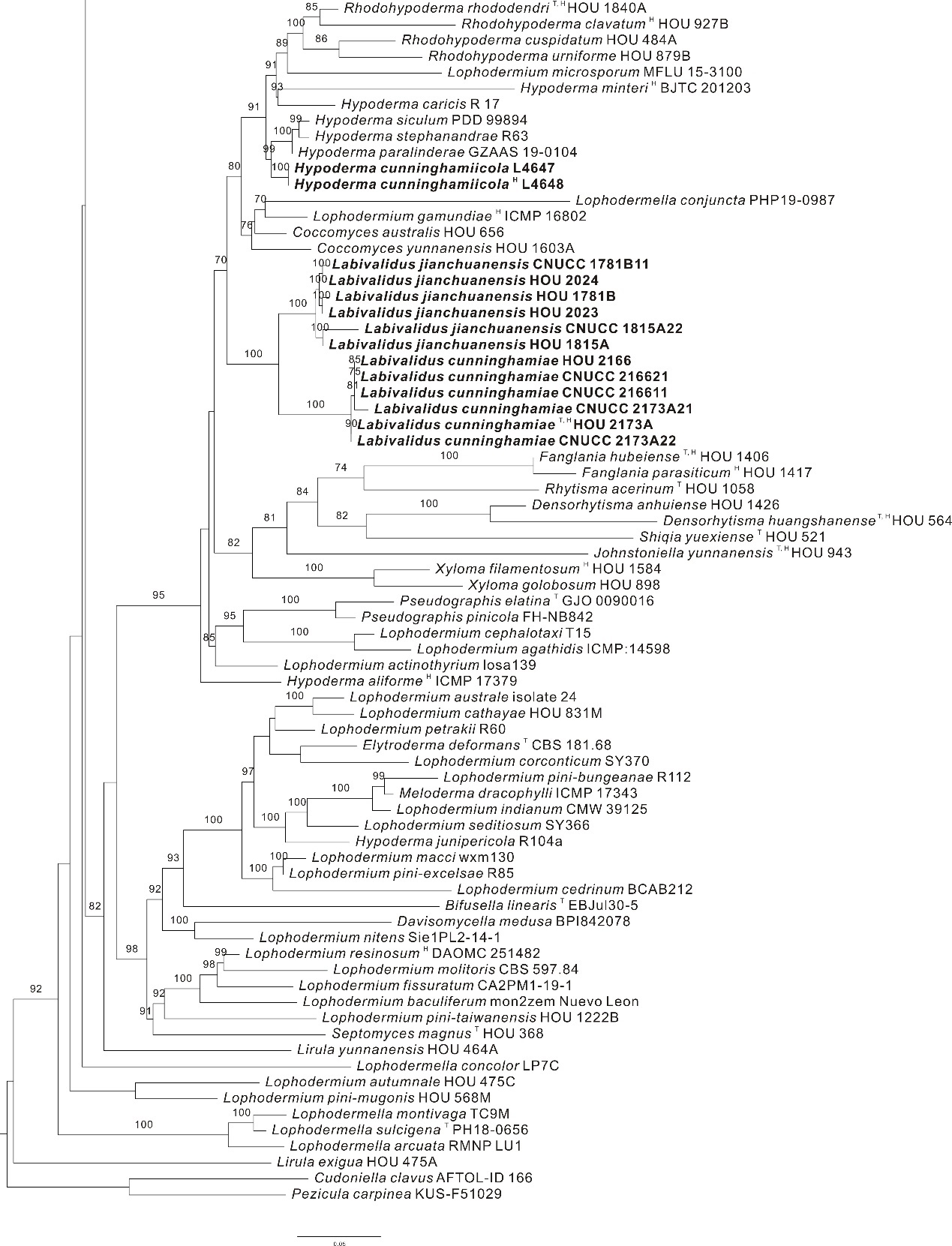


**Supplementary Fig. 1** – Phylogenetic tree generated by ML analysis based on sequences of the ITS. *Cudoniella clavus* (AFTOL-ID 166) and *Pezicula carpinea* (KUS-F51029) were selected as outgroups. Maximum likelihood bootstrap values (MLB ≥ 70%) were shown at the nodes.

**
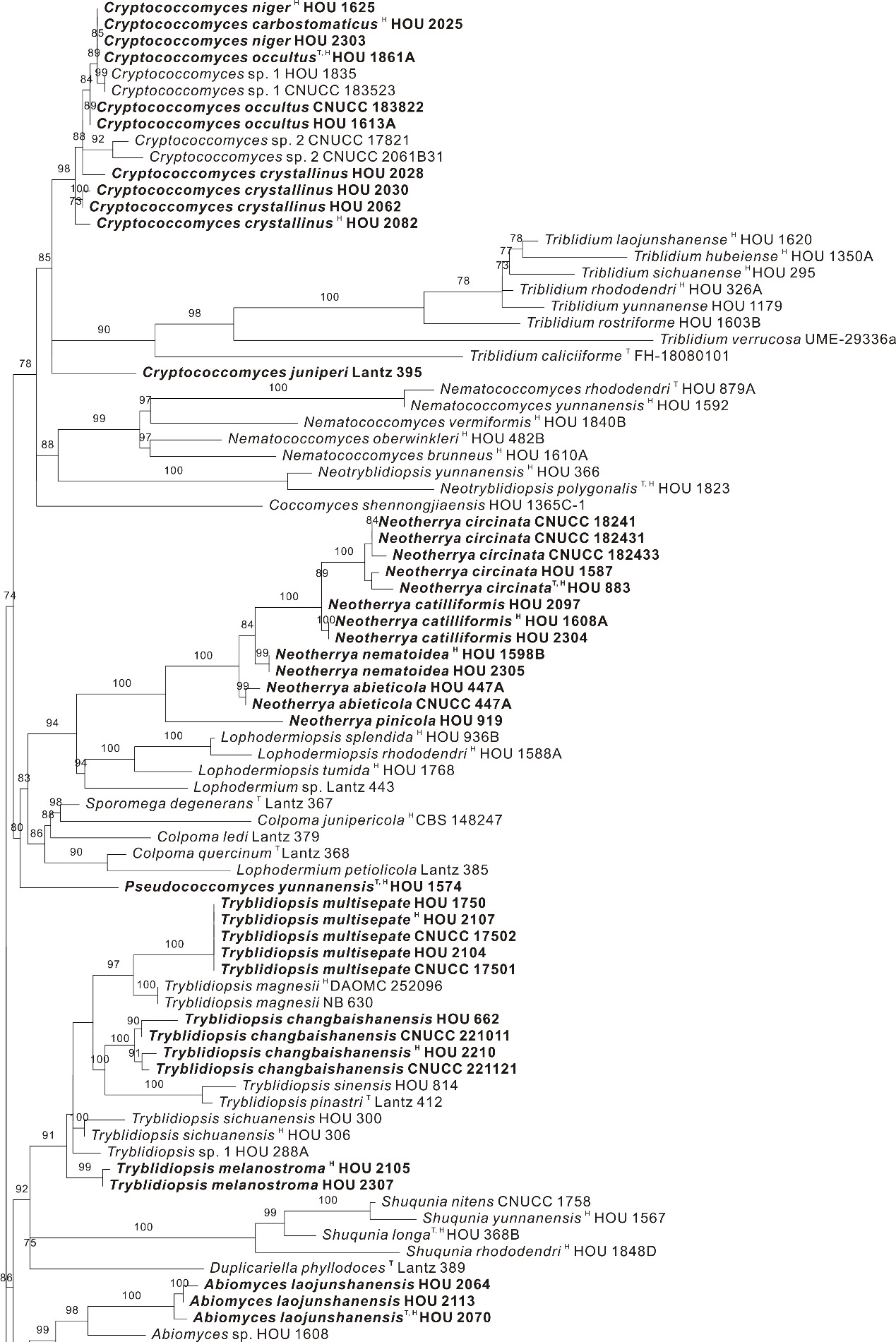
**


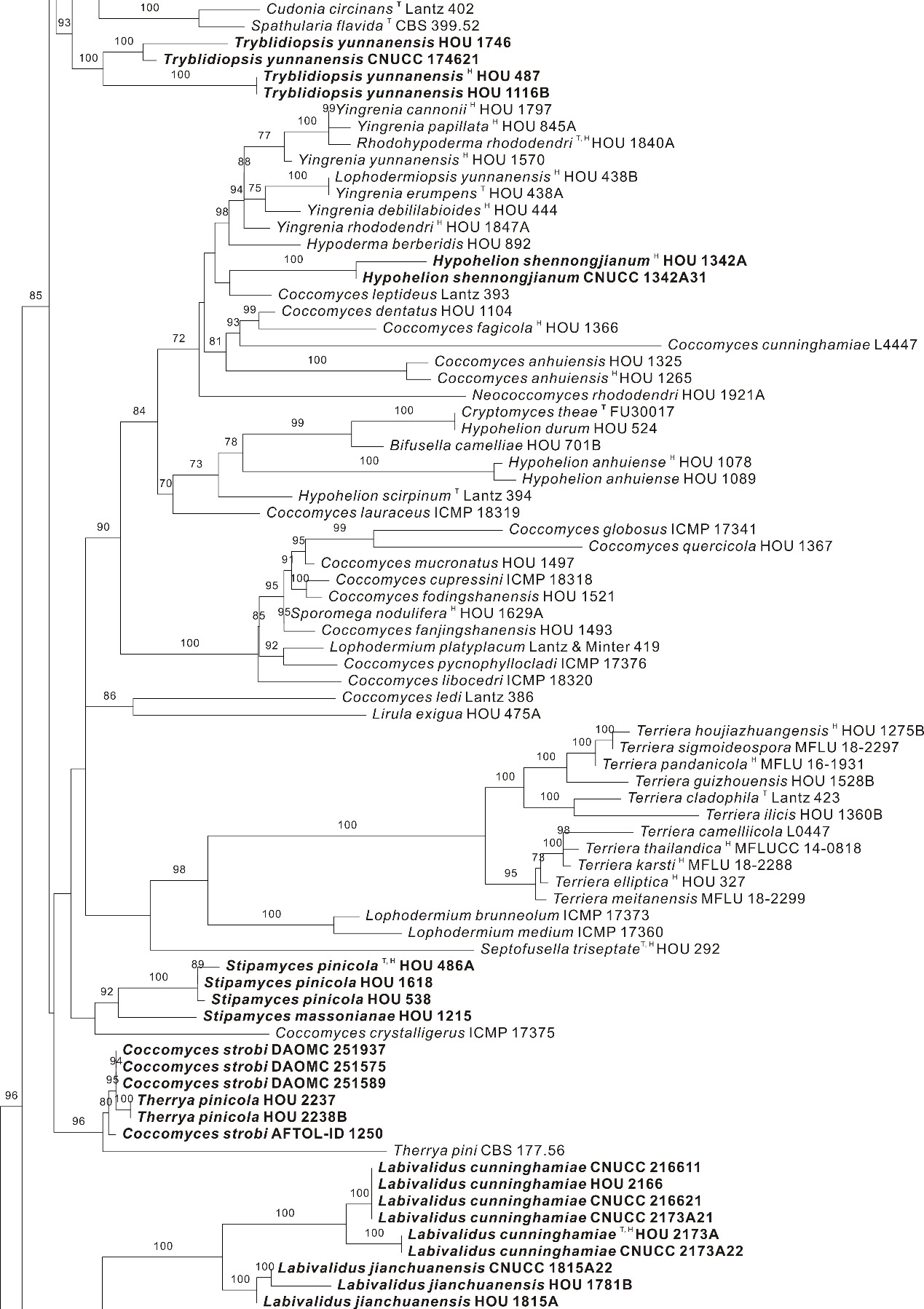


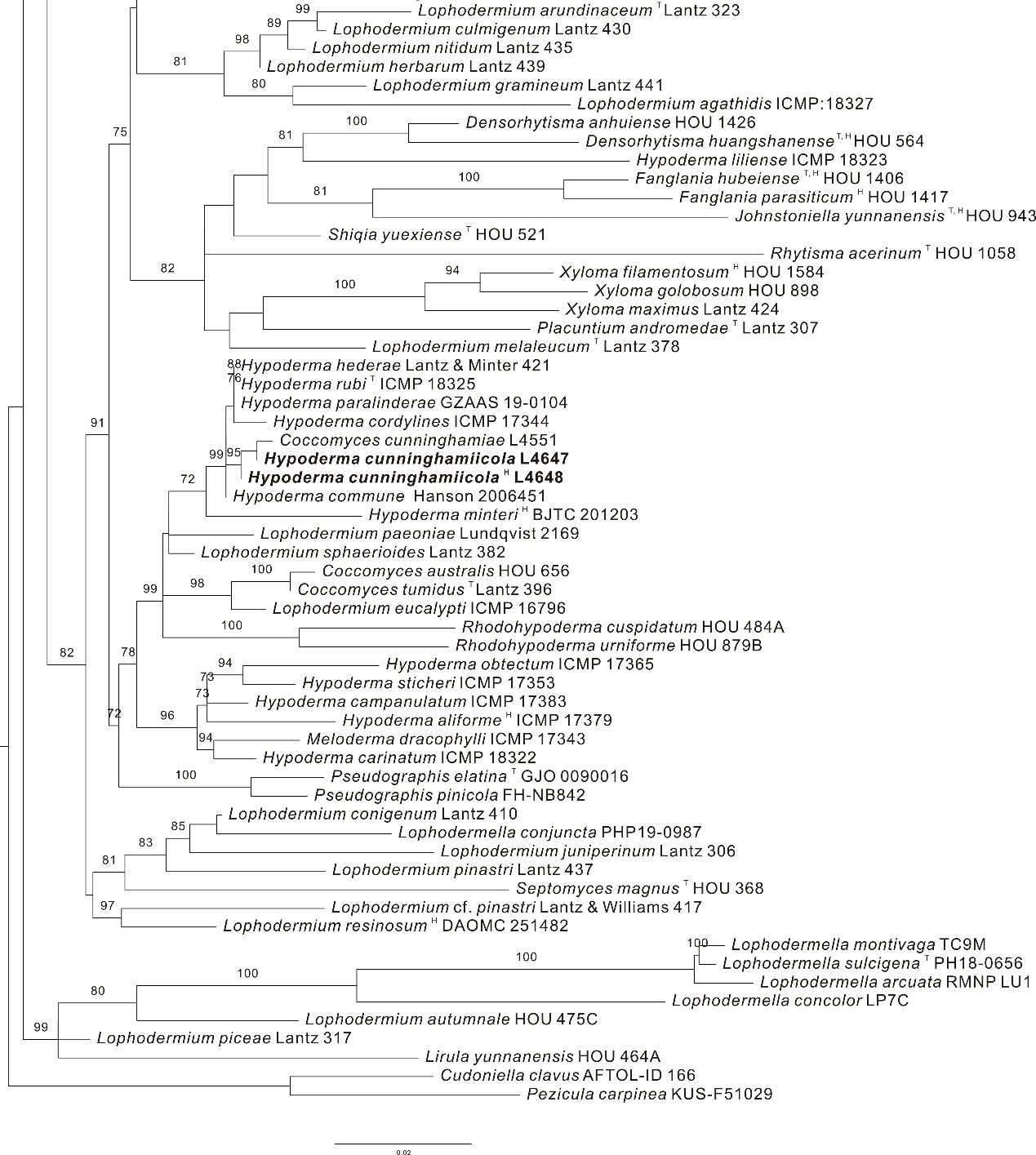


**Supplementary Fig. 2** – Phylogenetic tree generated by ML analysis based on sequences of the LSU. *Cudoniella clavus* (AFTOL-ID 166) and *Pezicula carpinea* (KUS-F51029) were selected as outgroups. Maximum likelihood bootstrap values (MLB ≥ 70%) were shown at the nodes.

**
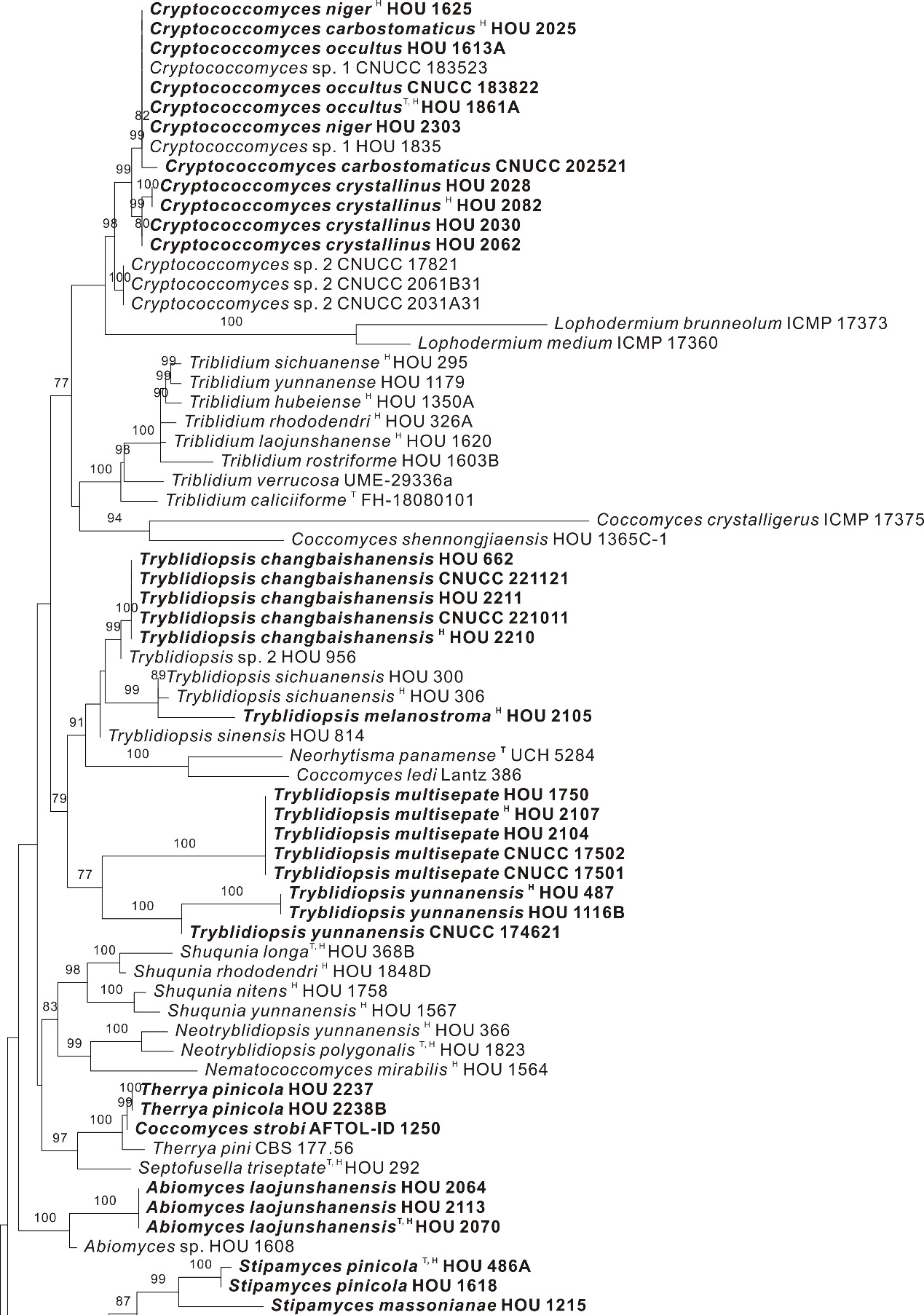
**


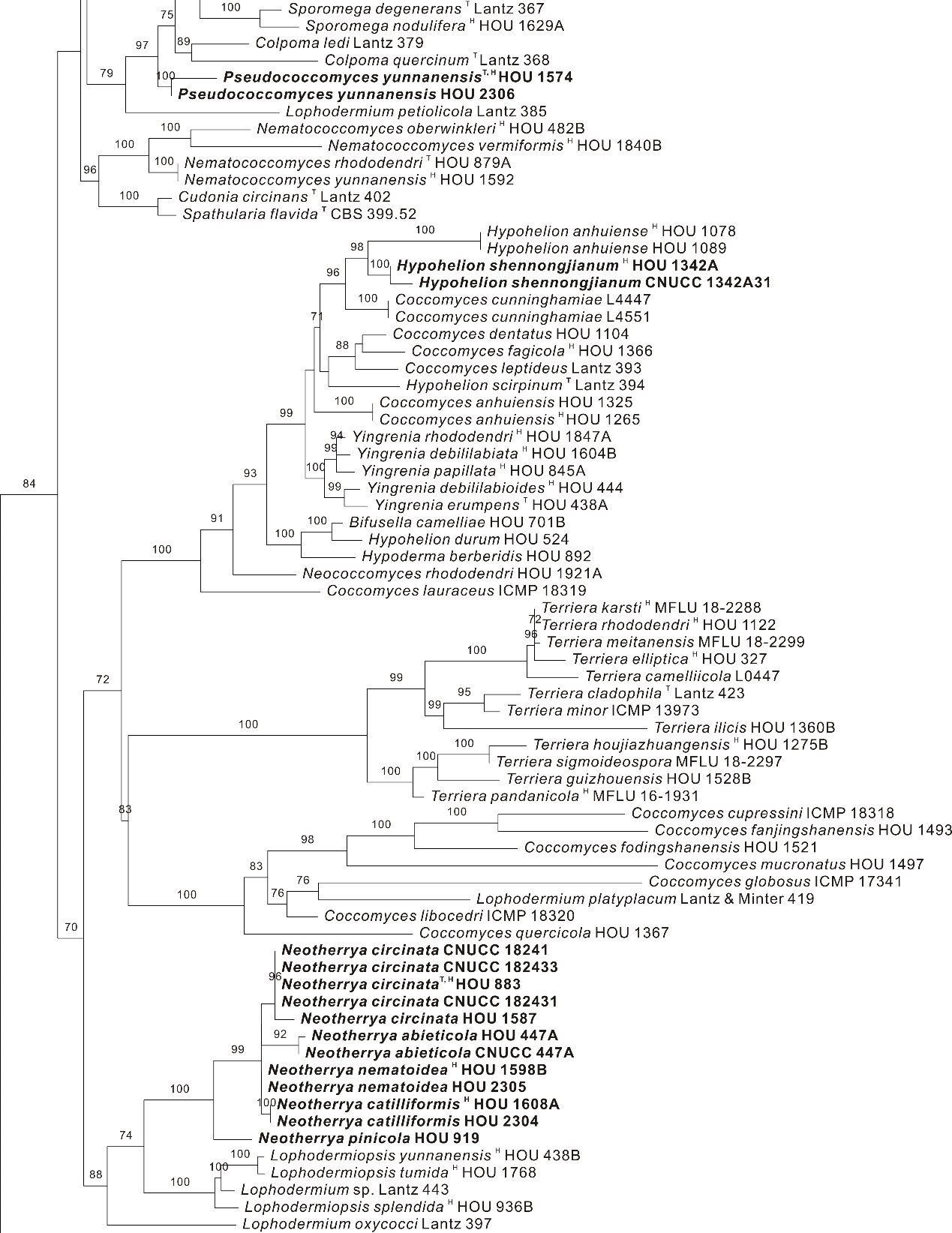


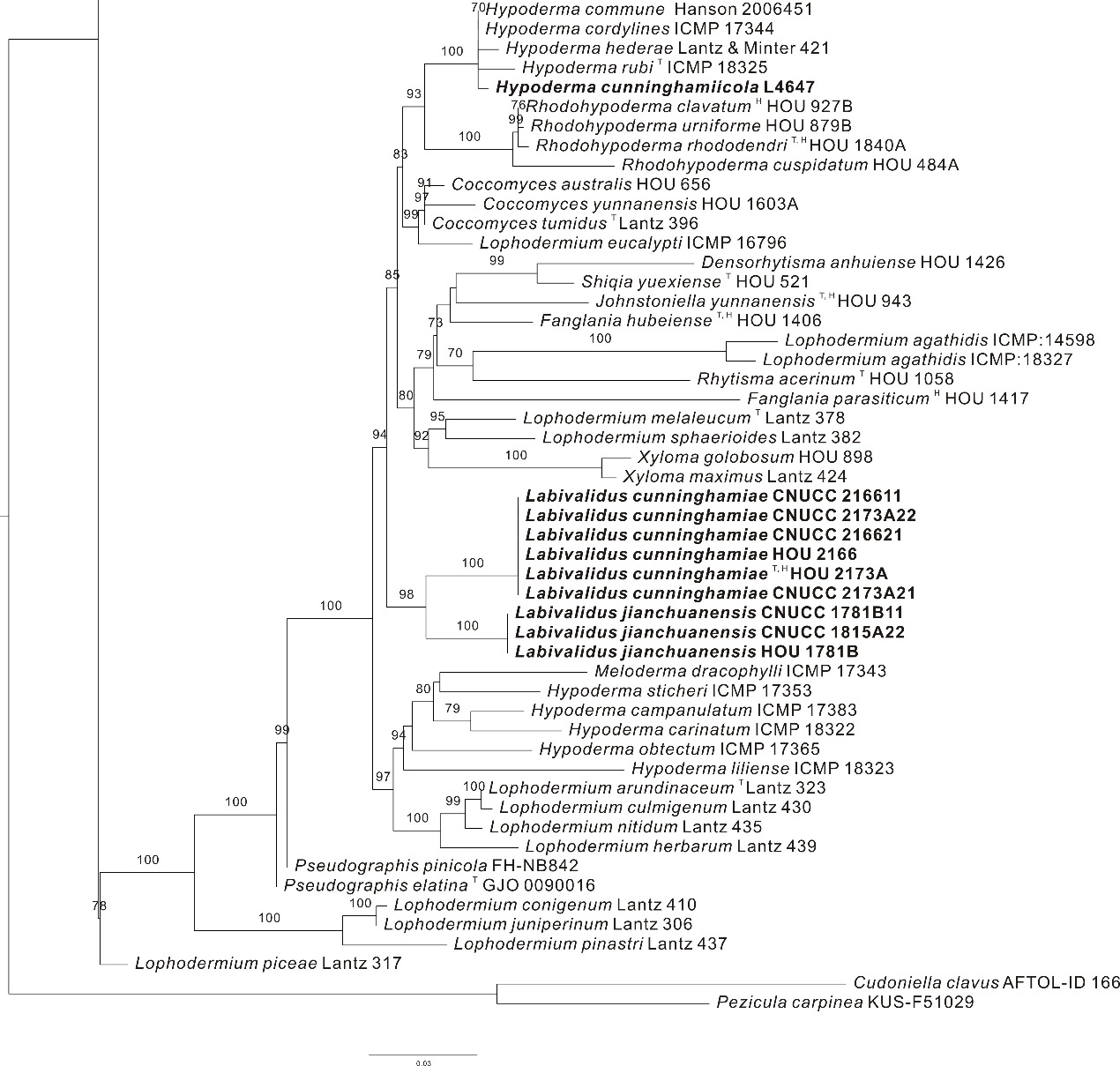


**Supplementary Fig. 3** – Phylogenetic tree generated by ML analysis based on sequences of the mtSSU. *Cudoniella clavus* (AFTOL-ID 166) and *Pezicula carpinea* (KUS-F51029) were selected as outgroups. Maximum likelihood bootstrap values (MLB ≥ 70%) were shown at the nodes.


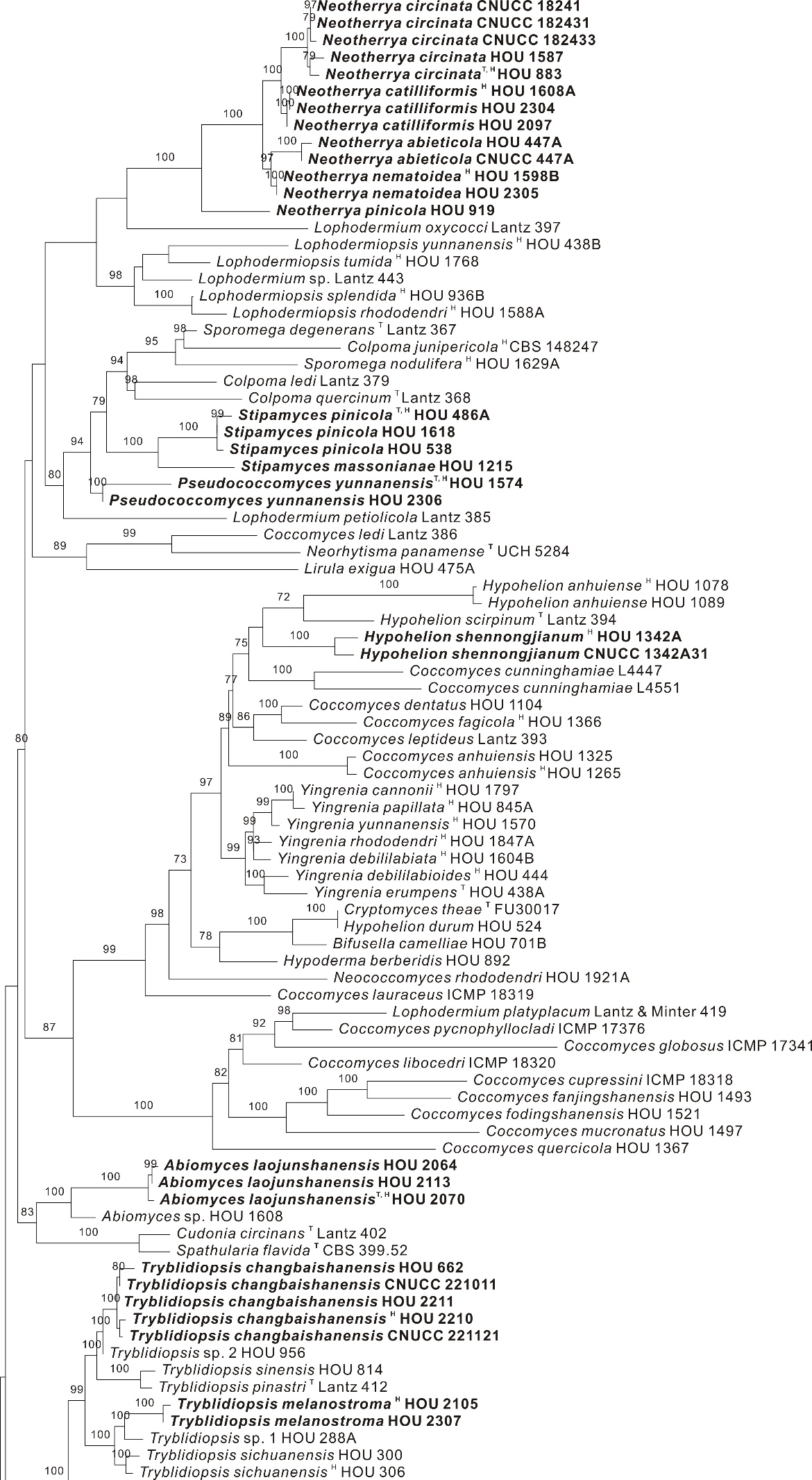


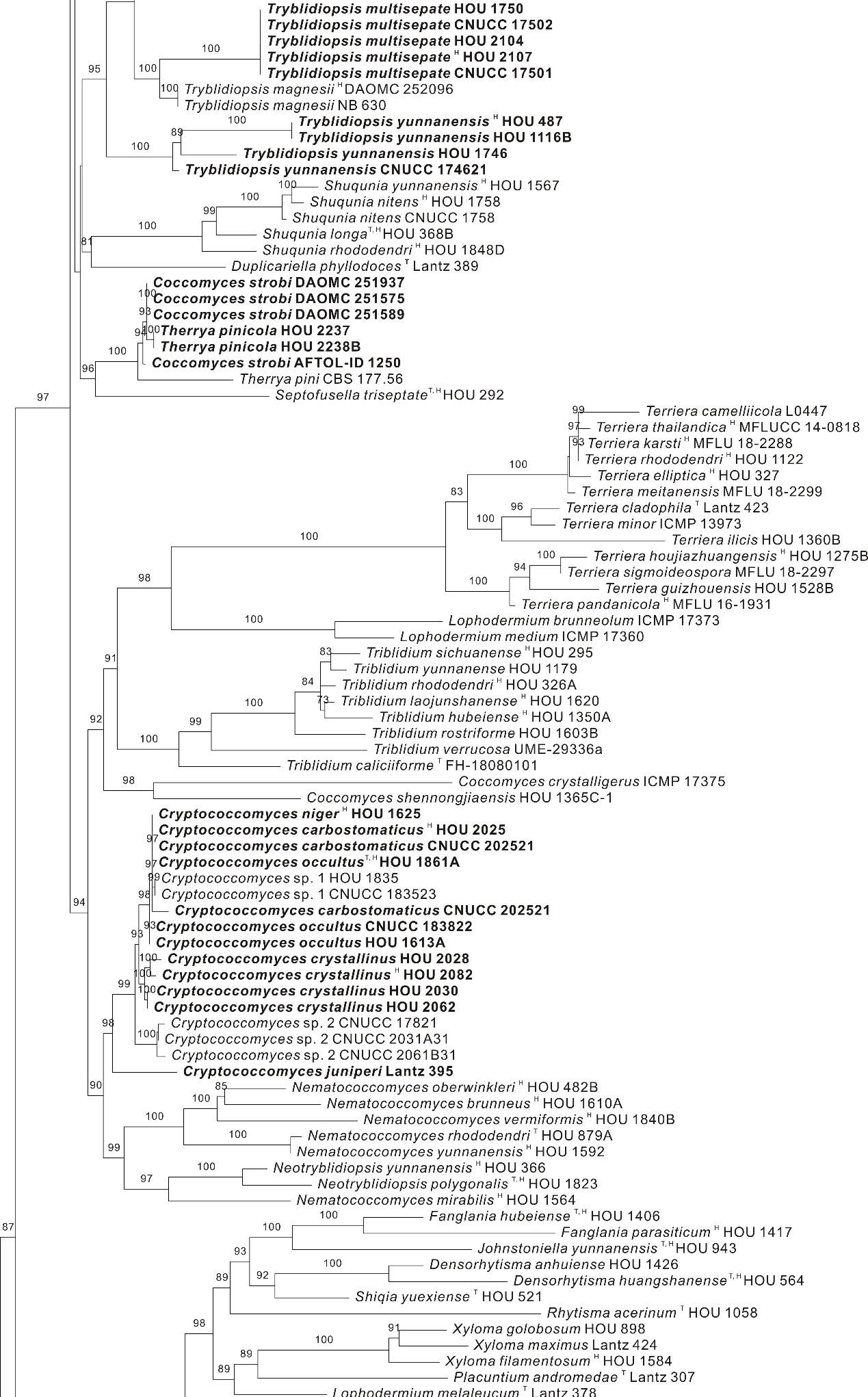


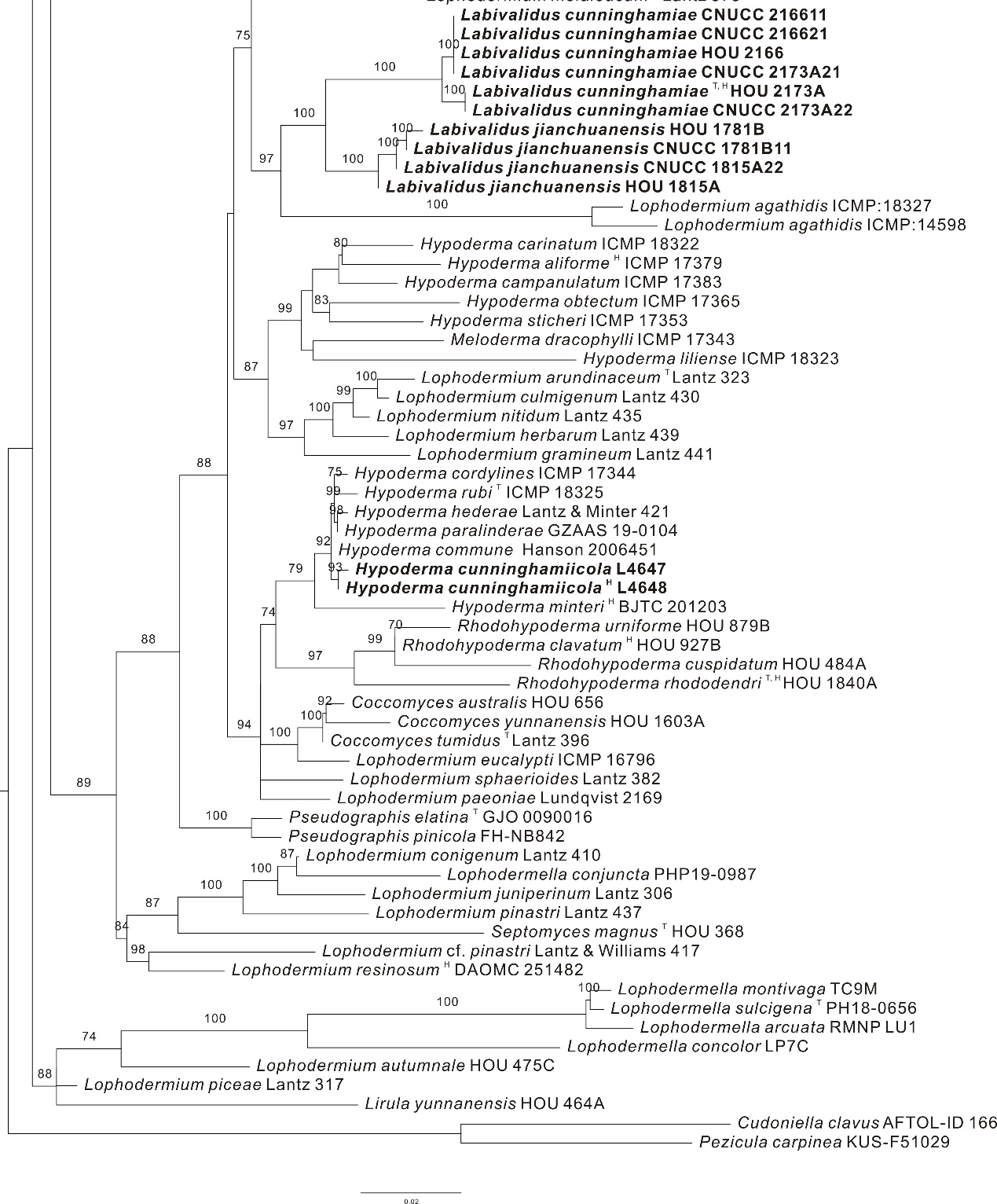


**Supplementary Fig. 4** – Phylogenetic tree generated by ML analysis based on sequences of the LSU and mtSSU. *Cudoniella clavus* (AFTOL-ID 166) and *Pezicula carpinea* (KUS-F51029) were selected as outgroups. Maximum likelihood bootstrap values (MLB ≥ 70%) were shown at the nodes.
